# Honey as a Natural Flavorful Product: A Comprehensive Review of Its Potential Biological Activities and Recent Studies

**DOI:** 10.1002/fsn3.71645

**Published:** 2026-04-17

**Authors:** Ecem Bolat, Ahmet Alperen Canbolat, Sümeyye Sarıtaş, Mauro Lombardo, Anna Maria Witkowska, Hesham El‐Seedi, Mikhael Bechelany, Sercan Karav

**Affiliations:** ^1^ Department of Molecular Biology and Genetics Çanakkale Onsekiz Mart University Canakkale Turkiye; ^2^ Department for the Promotion of Human Science and Quality of Life San Raffaele Open University Rome Italy; ^3^ Department of Food Biotechnology Medical University of Bialystok Bialystok Poland; ^4^ Chemistry Department, Faculty of Science Islamic University of Madinah Madinah Saudi Arabia; ^5^ Institut Européen des Membranes (IEM), UMR 5635 University of Montpellier, Place Eugène Bataillon ENSCM, CNRS, CEDEX Montpellier France

**Keywords:** antimicrobial agents, antioxidant, bioactive phytochemicals, health foods, honey

## Abstract

Honey is a natural and nutritious product of the honey bee ( *Apis mellifera* ) with low water content. Its color ranges from white to amber, with taste varying accordingly. The chemical form and viscosity of honey depend on its composition. It mainly contains sugars and water, along with proteins, enzymes, polyphenols, vitamins, and minerals. Used for centuries as both food and medicine, honey also serves in alternative treatments. Its bioactive molecules such as phenolics, flavonoids, and enzymes exhibit antimicrobial, antioxidant, anti‐inflammatory, anticancer, and antidiabetic properties. Moreover, it offers cardio‐ and neuroprotective benefits, reinforcing its medicinal value. Honey promotes wound healing, infection prevention, and tissue regeneration. Its antimicrobial activity, primarily driven by hydrogen peroxide and phytochemicals, is effective against wound and burn infections. It may also relieve asthma, support digestion, and help prevent cancer. Recent studies have highlighted honey's involvement in key bioactive mechanisms, including modulation of oxidative stress, regulation of inflammatory pathways, and enhancement of cellular repair processes. Varieties such as Manuka, Tualang, and Sidr show distinct therapeutic effects depending on their botanical and geographical origins. Understanding their composition is key for developing targeted therapeutic applications. This review explores honey's composition and therapeutic potential, emphasizing its rich bioactive profile, which continues to attract scientific interest for treating various health conditions and promoting overall well‐being.

## Graphical abstract

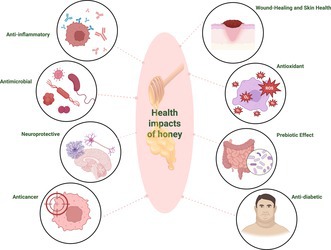

## 1 Introduction

**FIGURE 1:**
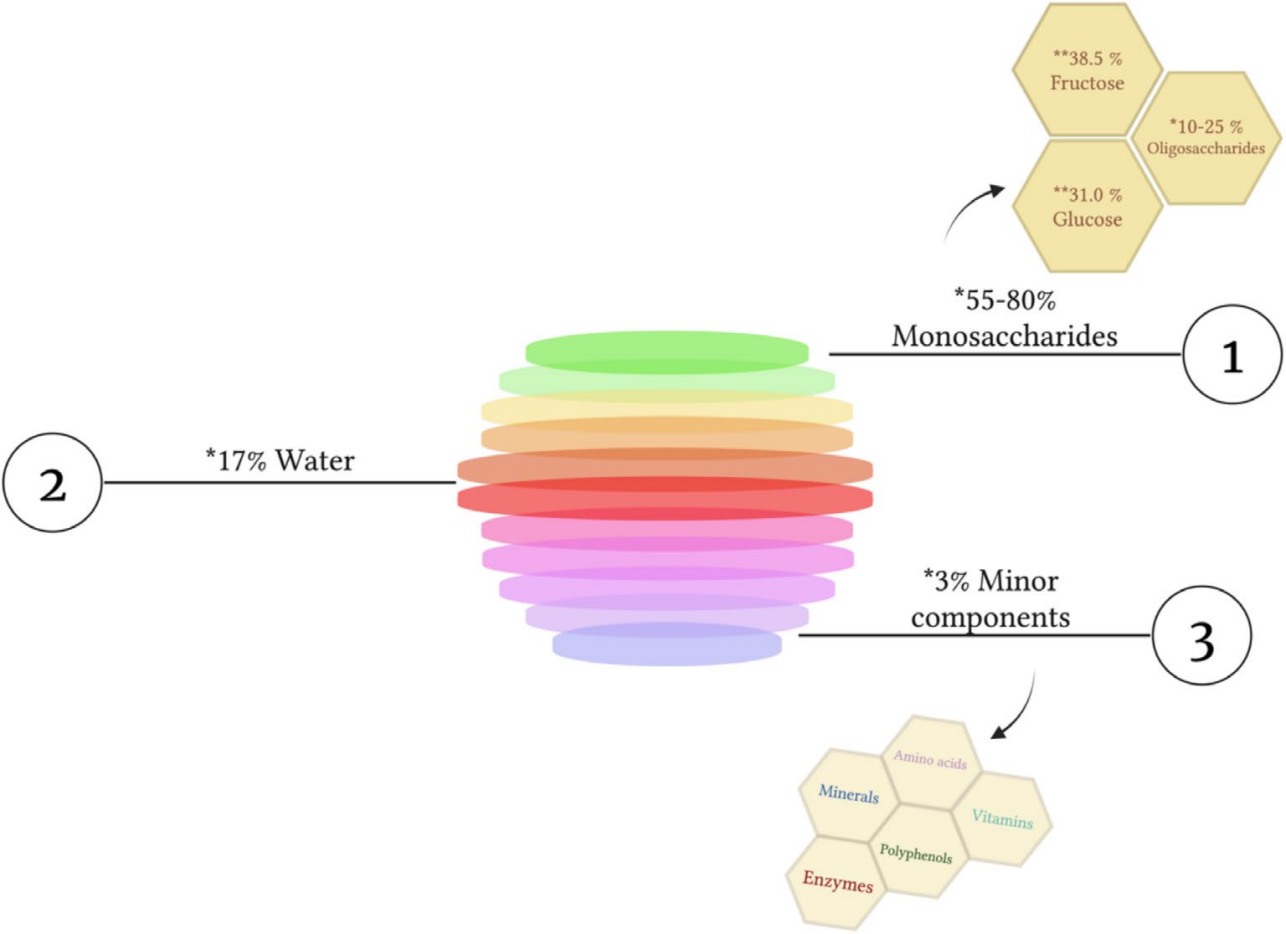
Schematic illustration of honey composition. * (Sanz et al. 2005; Hossain et al. 2022), ** (All About Honey 2024).

Honey is a natural flavorful and nutritionally healthy product of bees and contains reduced water composition (Alvarez‐Suarez et al. 2013). Honey can be produced when generally two types of bees including *Apis mellifera* (honey bee) and stingless bees ( *Meliponula ferruginea* ) chemically convert plants into nectar, secretions, and insect secretions on the plant (Becerril‐Sánchez et al. 2021). Bees can collect nectar from different flowers and store honey as a nutrition source to use during winter (Jaganathan and Mandal 2009). They gather nectars from various plant flowers by flying around approximately 88.513 km to make 0.45 kg of honey and by using their wings, they can fan honey leading to water to evaporate and providing protection against fermentation (Jaganathan and Mandal 2009). Additionally, the color of honey is evaluated with the Pfund scale, which varies from 0 to 140 mm (PFund Color Grading 2024), and higher Pfund values mean the darker color of honey (Durmishi et al. 2023). The honey color is one of the parameters evaluated for classification. Although this color scale ranges from white to black, researchers have linked the color of honey to its phenolic and flavonoid content, with the darkest honey having the highest phenolic content values (Hernanz et al. 2023). The diversity of the content of honey and its physical characteristics can differ according to the bee species, floral or other manufacturing source, temperature, and geographical area (Ranneh, Akim, et al. 2021). Due to these varieties, the color, taste, viscosity, content, and potential health impacts of honey also vary drastically (Rao et al. 2016). However, in general, honey contains many compounds such as carbohydrates, proteins, enzymes, vitamins‐minerals, amino acids and different types of polyphenols, and the ratio of these compounds varies depending on the factors affecting the variety of honey (Ranneh, Akim, et al. 2021). Monofloral and polyfloral are the primary botanical classifications of honey. The type of monofloral honey is derived from a single plant species, while the polyfloral type comes from diverse floral sources (Al‐Awadhi and Deshmukh 2021). Additionally, honey can be classified according to its origin such as floral (nectar) or honeydew, which is manufactured from insect secretions (Tarapatskyy et al. 2021). According to honey's geographical origin with a specific type from a special area, honey can be categorized, including Manuka honey (New Zealand), Sidr Honey (Middle East), and Himalayan honey (Soares et al. 2017). Moreover, the processing steps of honey can impact the classification of honey, such as pasteurization (pasteurized honey), filtration (filtered honey), extraction (raw honey), harvesting (organic honey), and consumption in its natural form is comb honey (Tian et al. 2024). In all these classifications, honey is composed of similar main components, although the content composition ratio and physical form may differ. In general, honey is rich in natural bioactive compounds mainly carbohydrates and water, also including proteins, enzymes, vitamins and minerals, amino acids, and polyphenols (phenolic compounds) (Baloš et al. 2020). Honey is mainly composed of 55%–80% glucose and fructose, known as monosaccharides, while 10%–25% is a complex mixture of carbohydrates such as sucrose, maltose, turanose, and other oligosaccharides (Chen et al. 2022; Sanz et al. 2005). The remaining 3% of honey is a mixture of enzymes, proteins, amino acids, various polyphenols, minerals, vitamins and about 17% water (Figure 1) (Hossain et al. 2022).

The bioactive constituents depend on particular factors, which can affect the diversity of honey and are considered crucial due to having biological health impacts (Jaganathan and Mandal 2009; Masri et al. 2024). Therefore, these bioactive constituents are known to have potential therapeutic healing effects from the past to the present (Sanz et al. 2005). Throughout history, honey has not only been used as a food or sweetener (Wang, Li, Lin, et al. 2023), but also as a remedy for various diseases and health issues, thanks to its bioactive components (Sanz et al. 2005). The use of honey as a medicine has been realized in the presence of bioactive properties such as anti‐inflammatory, antimicrobial, anticancer, anti‐diabetic, cardio, neurological, gut protection, antioxidant, and wound repair.

In line with the research in the literature, the bioactive properties of honey are attributed to the presence of various molecules, including compounds such as flavonoids‐phenolic acids, proteins, enzymes, oligosaccharides, vitamins, and minerals. Various in vitro and in vivo studies to date have shown that honey has biological activities and has been proven to have potential health‐protective effects (Cianciosi et al. 2018). Owing to these health effects, it is reported to be useful in the treatment of various diseases, especially gastrointestinal, cardiovascular, neurodegenerative issues and diabetes mellitus, wound‐healing, anti‐aging and some types of cancer (Cárdenas‐Escudero et al. 2023). Although these bioactive molecules are still being investigated, researchers have performed various studies to evaluate the therapeutic effects of honey (Masri et al. 2024). According to a recent study, phenolic acids and oligosaccharides found in Buckwheat honey can regulate the human gut microbiome (Jiang et al. 2020). It has been shown that they synergistically affect human gut flora to stimulate the population of probiotics and moreover, polyphenols act as the main character of modulation in the gut microbiota. In addition, in another study, the antimicrobial activity of honey phenolic components on *Escherichia coli* was researched. Also, chronic wound patients were included in the study and *p*‐coumaric acid (PCA), one of the phenolic components, was observed to increase antimicrobial activity, showing its potential as a new antimicrobial molecule of honey content in the treatment of skin infections or lesions (Kassym et al. 2024). Furthermore, a global health issue, which is diabetes mellitus and specificized by hyperglycemia with numerous damaged tissues also may be treated with honey due to its rich antioxidant content (El‐Aarag et al. 2024). It can play a role as defense compound like an intracellular antioxidant against reactive oxygen species (ROS) in diabetes mellitus. Additionally, its antioxidant contents also have potentially advantageous effects on blood glucose due to their improving characteristics on hyperglycemia (Ofor et al. 2022). For instance, a recent study aimed to show that modulation of ROS and caspase‐mediated cell death in the diabetic rats with the treatment of Egyptian sidr honey. Researchers illustrate the antidiabetic affinity of Egyptian type honey, which regulates the glucose levels and ameliorates the oxidative stress markers. Moreover, researchers showed that this type of honey can regulate the activity of antioxidant enzymes and to improve the protein generation levels, it enhances immunohistochemical alterations in liver and pancreatic tissues (El‐Aarag et al. 2024). In a different aspect, a popular gastrointestinal disorder, which is gastritis, generally occurs in consideration of *Helicobacter pylori* infection (Akanda and Park 2017). The consequence of a recent study showed that the chestnut honey possesses an anti‐inflammatory role on gastritis by alleviating gastric mucosal damage and upregulating the expression of antioxidant enzymes. Therefore, researchers suggested that this type of honey can be potentially functional health food candidate (Kim et al. 2024). Also differently, in a recent preclinical model study, researchers have shown that a different type of honey, manuka honey, prevents human breast cancer, which is widely known to cause the death of many women worldwide (Márquez‐Garbán et al. 2024). Researchers demonstrated that phenolic compounds of manuka honey possess antioxidant, antiseptic, and anticancer roles. Therefore, oral supplementation of manuka honey inhibited the growth of tumors without side effects, and they suggested that this type of honey possesses the potential to be a cytotoxic anticancer drug. Another recent study demonstrated the neurodegenerative blocking impact of honey combinations on mice models with Parkinson's diseases. This honey combination provided protection of dopaminergic neurons against induced Parkinson's disease and improved neurological abnormalities (Adeyemo Emmanuel Ayobami 2020). As a consequence of these all recent studies, different types of honey have the potential to be a treatment agent for health issues. The fact that all the honey samples in these studies were of different types, with different content ratios and differences in potential health benefits, as well as the wide variety of honey, diversifies the potential for therapeutic effects.

**FIGURE 2:**
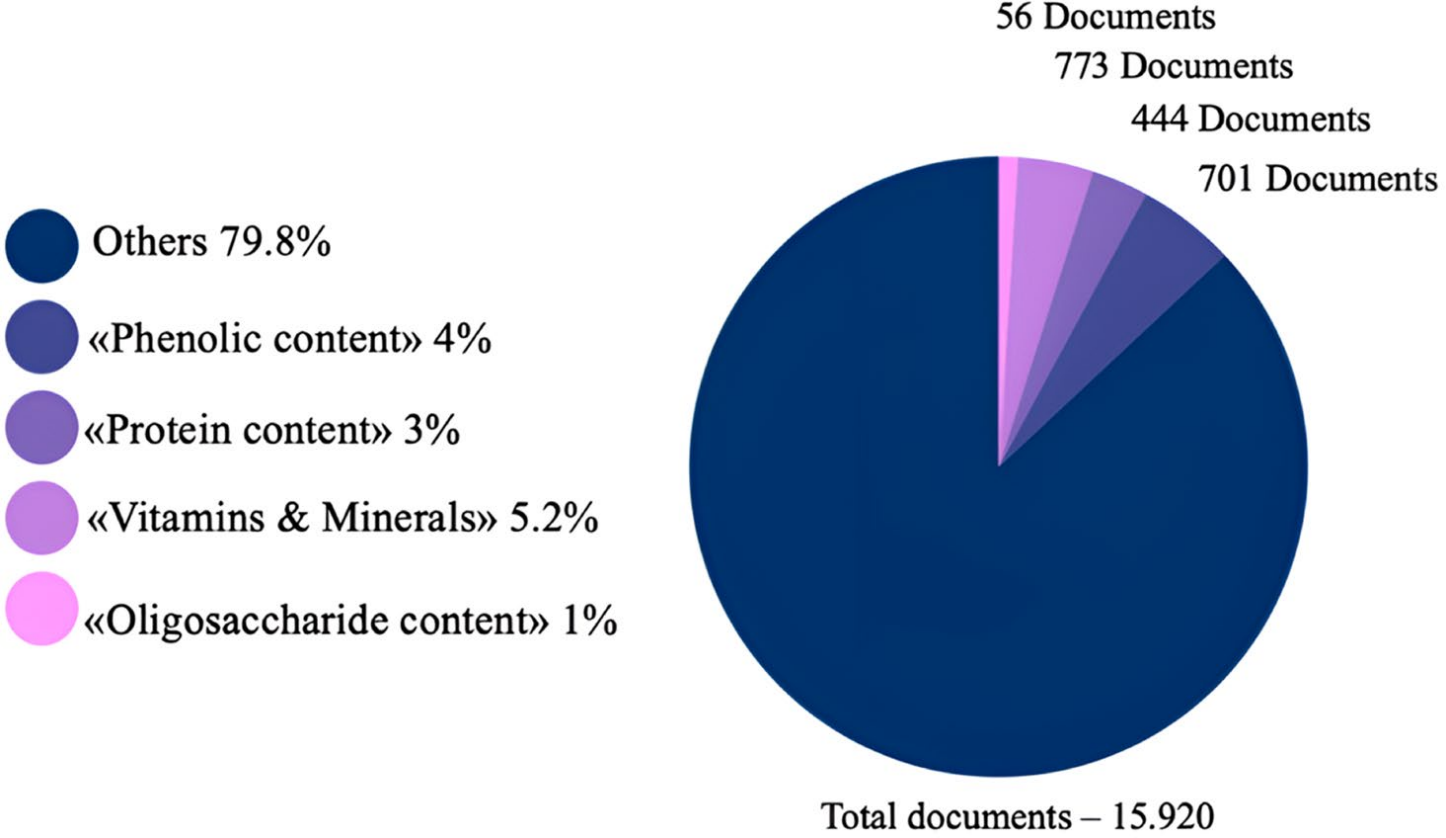
According to data from the Web of Science platform, a graphical chart of bioactive content of honey studies entitled “which (protein, phenolic, etc.) bioactive content” for the last 5 years (Clarivate 2024).

Moreover, although honey studies in the literature have recently attracted the attention of researchers, studies evaluating honey bioactive components are very limited and it is important to bring new studies to the literature (Figure 2). The introduction of new studies may pave the way for further studies on the therapeutic effects of honey.

Therefore, this review article aims to compile the important components of honey, a natural mixture, and how these components have health benefits with current recent in vivo, in vitro, and possibly clinical studies investigating the biological properties of honey to highlight the potential different therapeutic effects of different honey varieties, to gather new data on the health benefits of various kinds of honey in the literature and to highlight gaps in the literature. In addition, there is a lack of clinical and animal model studies evaluating honey and its bioactive content. Furthermore, this review also emphasizes the underlying mechanisms by which honey exerts its beneficial effects, such as antioxidant, anti‐inflammatory, antimicrobial, and immunomodulatory activities mediated by its bioactive compounds. In this review, it is also aimed to show that different honey types have various therapeutic effects by including studies on different honey types in honey research in the literature.

## 2 Therapeutic Mechanisms of Honey

Honey's diverse bioactive compounds grant it regulatory properties over multiple biological functions, which are linked to a wide range of health benefits. Its phenolic compounds, flavonoids, and enzymatic components play a central role in alleviating oxidative stress, controlling inflammatory pathways, and modulating intracellular (Ahmed et al. 2018; Wilczyńska and Żak 2024). By influencing these mechanisms, honey may interfere with disease‐related pathogenic processes, making it a promising natural therapeutic or supportive (Saad 2025; Hashim et al. 2021).

**FIGURE 3:**
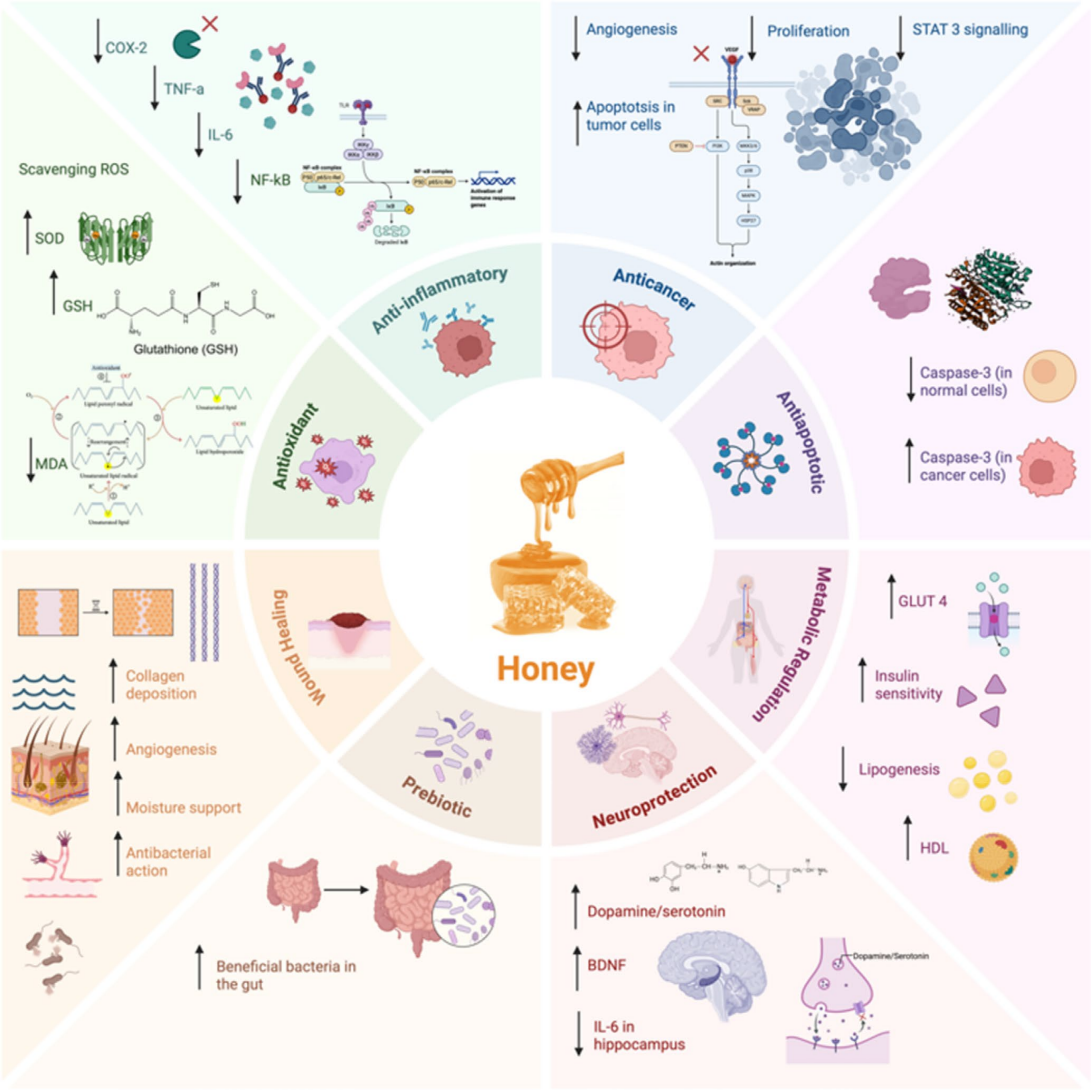
Biochemical and molecular mechanisms of honey.

Beyond its antioxidant and anti‐inflammatory activities, honey also contributes to health promotion through its immunomodulatory effects, antimicrobial action, and capacity to enhance tissue (Tashkandi 2021). Taken together, these multifactorial properties highlight honey as a valuable natural resource for both the prevention and management of various diseases (Naqvi et al. 2023). Honey is more than just a foodstuff; it also has many therapeutic effects thanks to the bioactive compounds it contains. Honey's capacity to suppress pathogenesis and exert positive effects on health through various molecular mechanisms (Figure 3) makes it an important complementary agent in modern medicine. Honey's role in disease management can be explained through its mechanisms of action, particularly the reduction of oxidative stress, the balancing of the inflammatory response, and the modulation of cellular signaling pathways (Ahmed et al. 2018).

### 2.1 Modulation of Oxidative Stress and Apoptosis

Honey exhibits dual regulatory effects on oxidative stress and apoptosis due to its phenolic compounds, flavonoids, and enzymes. Oxidative stress arises from the production of ROS (reactive oxygen species)—such as superoxide (O_2_^−^), hydroxyl radical (•OH), and hydrogen peroxide (H_2_O_2_)—and insufficient detoxification of these compounds (Hayes et al. 2020). This imbalance can trigger apoptosis, or programmed cell death, by disrupting cell functions through lipid peroxidation and DNA and protein damage (Kannan and Jain 2000). In particular, disruption of the mitochondrial membrane potential, release of cytochrome c, and activation of caspase‐9/3 constitute the main execution mechanisms of apoptosis. This process can occur via both intrinsic (mitochondrial) and extrinsic (death receptor‐mediated) pathways (Kannan and Jain 2000). The bioactive polyphenols in honey (e.g., quercetin, kaempferol, gallic acid, pinocembrin) activate the Nrf2 transcription factor, thereby enhancing endogenous (intracellular) antioxidant defense (Wilczyńska and Żak 2024; Gheldof et al. 2002; Ahmed and Othman 2013). Intracellular antioxidant enzymes such as superoxide dismutase (SOD), catalase (CAT), and glutathione peroxidase (GPx) play a critical role as an intracellular defense mechanism (Khalil et al. 2011). SOD converts superoxide into the less reactive form hydrogen peroxide; catalase converts this peroxide into water and oxygen, while GPx inactivates lipid hydroperoxides and H_2_O_2_ (Jeeva et al. 2015). These enzymes not only reduce oxidative damage but also significantly contribute to maintaining cellular redox balance (Chaudhary et al. 2023).

Apoptosis is a genetically programmed, orderly cell death process that serves to limit cellular damage. Polyphenols or phenolic compounds found in honey not only exhibit antioxidant effects but can also demonstrate pro‐apoptotic effects (Jaganathan and Mandal 2009). For example, while quercetin plays an antioxidant role in cells at low doses, it can trigger apoptosis by increasing ROS levels at high doses (Tlak Gajger et al. 2025). Pinocembrin has been shown to regulate intrinsic apoptotic pathways activated by mitochondrial dysfunction or DNA damage, helping stabilize mitochondrial membrane potential and modulate p53 and caspase signaling. In this way, it can promote caspase‐3 activation in certain tumor cells while preventing excessive apoptosis in normal cells (Ahmed et al. 2018; Ahmed and Othman 2013; Tlak Gajger et al. 2025; Al‐Hatamleh et al. 2020). Overall, honey functions as a versatile biological agent by limiting ROS formation, enhancing antioxidant defenses, and modulating apoptosis‐related mechanisms, thereby supporting cellular integrity (Jaganathan and Mandal 2009).

Supporting this, a 2025 study (Aati et al. 2025), reported that Saudi Tamarix honey (STH) provided protection against cisplatin (CIS)‐induced renal damage in rats, highlighting its role in counteracting the oxidative stress, inflammation, and apoptosis associated with this chemotherapeutic drug. In the experiment, rats were pre‐treated with STH (50 or 100 mg/kg) for 10 days and given a single CIS dose (7 mg/kg) on Day 7. CIS exposure triggered severe glomerular and tubular damage, elevated lipid peroxidation, pro‐inflammatory cytokines (TNF‐α, IL‐6), cleaved caspase‐3, and reduced antioxidant levels. STH supplementation significantly recovered these side effects by preventing tissue damage, reducing oxidative stress and inflammatory markers, suppressing caspase‐3 and STAT3 protein expression, and enhancing Nrf2 signaling. Metabolomic profiling also revealed 15 bioactive metabolites in STH, mainly phenolic acids, flavonoids, and sterols, with phenolic acids being most abundant. In general, STH demonstrated strong nephroprotective effects by modulating oxidative stress, inflammation, and apoptosis pathways in CIS‐treated rats. This recent study highlighted the nephroprotective potential of Saudi Tamarix honey (STH) against cisplatin‐induced kidney injury. Briefly, STH reduced oxidative stress by enhancing antioxidant enzymes (e.g., SOD) and lowering lipid peroxidation (MDA), while also suppressing pro‐inflammatory cytokines (TNF‐α, IL‐6) and inhibiting caspase‐3 activity, indicating anti‐apoptotic effects. Mechanistically, STH upregulated Nrf2, which promotes antioxidant defense, and downregulated STAT3, a pro‐inflammatory regulator, demonstrating a multi‐target protective action. Metabolomic analysis revealed abundant phenolic acids and other bioactive compounds, supporting the link between phenolic metabolites and antioxidant activity. The findings provide a strong basis for future research aimed at identifying specific active compounds, elucidating their roles in nephroprotection, and exploring the precise molecular mechanisms through which STH modulates Nrf2 and STAT3 signaling pathways. Another study, which investigated the antioxidant‐rich bee product mixture (ARPM) composed pf honey, pollen, propolis, and royal jelly both in vivo and in vitro models, revealed the doxorubicin‐induced ovarian injury in female Sprague–Dawley rats (Malkoc et al. 2025). The chemotherapeutic agent, doxorubicin, is a well‐known oxidative agent and also triggers endoplasmic reticulum (ER) stress, leading to reproductive tissue damage. The introduction of ARPM significantly improved systemic ovarian function. Bioactive molecules, such as chrysin, pinocembrin, caffeic acid, ferulic acid, and coumaric acid, increased the activity of superoxide dismutase (SOD) and glutathione (GDH) while reducing malondialdehyde (MDA) activity, as well as relieved ER stress markers such as GRP78, IRE1, CHOP, TNF‐α, and caspase‐3. The active components of ARPM were found to restore estradiol and progesterone levels as well as the histological structure of the ovaries. Its protective effects appear to involve modulation of the ER stress pathway and maintenance of hormonal balance. As a functional food supplement, ARPM shows promise in supporting reproductive health during chemotherapy, though further research is needed to clarify its mechanisms and therapeutic value. Similaryl, Ruslee et al. (2020) demonstrated that Tualang honey protected against cadmium‐induced ovarian toxicity in rats. While cadmium increased oxidative stress, disrupted hormone levels, and impaired ovarian morphology, Tualang honey treatment reduced oxidative damage, normalized hormones, and improved tissue structure. These findings suggest that natural antioxidants like Tualang honey may help preserve ovarian health under toxic conditions. This study demonstrates the protective effects of Tualang honey against ovarian toxicity, supporting the use of natural products in reducing toxic effects.

From ancient times, honey has been a valuable source as both a food and a medicinal application (Chari 2008). With many of the phenolic compounds, honey is considered a powerful biological food source in tumors and several cancer types (Ahmed et al. 2018; Can et al. 2015; Erejuwa et al. 2014). The anticancer activity of honey has been investigated against distinct cancer types and cell lines, namely colorectal, breast, endometrial, prostate, renal, oral, and cervical cancers (Afrin et al. 2017; Othman 2012; Fauzi et al. 2011; Afshari et al. 2011). In fact, raw honey has been proven to enhance the efficiency of chemotherapeutic agents such as cyclophosphamide and 5‐fluorouracil. In the mechanism, it was observed that the polyphenols present in honey are the main bioactive ingredients responsible for these anticancer effects (Moniruzzaman et al. 2011). The findings of in vitro and in vivo studies have supported the anticancer effects (Waheed et al. 2019). Some remarkable studies have been discussed in this section. In a study conducted by Afrin et al. (2018), it was demonstrated that Manuka honey (MH) exhibited a synergistic effect when used in combination with 5‐fluorouracil (5‐FU) on human colon cancer cell lines (HCT‐116 and LoVo). This combination reduced cell proliferation by suppressing EGFR, HER2, p‐Akt, and p‐mTOR expression; activated pro‐apoptotic pathways (p53, Bax, cytochrome c, FasL, caspase‐3, ‐8, ‐9, and cleaved‐PARP); and decreased anti‐apoptotic Bcl‐2 levels. Additionally, reactive oxygen species (ROS) production increased, while NF‐κB, Nrf2, and antioxidant enzyme (SOD, catalase, glutathione peroxidase) activities decreased. Parameters related to metastasis were also negatively affected; cell migration decreased while MMP‐2/9 expression was suppressed, and increases in N‐cadherin and E‐cadherin were observed. Energy metabolism was also suppressed, causing cells to enter a quiescent phase. All these findings indicate that MH enhances the effect of 5‐FU, leading to death, oxidative stress, and reduced metastatic capacity in colon cancer cells, and that MH may be a potential adjuvant therapeutic agent when used in combination with 5‐FU. Derya Andeden et al. (2024) investigated the effects of honey obtained from Pervari district in Siirt province, Turkey (Pervari Honey, PH) on proliferation, oxidative stress, and apoptosis in human breast cancer cells. The study evaluated the total phenolic content and antioxidant capacity of PH. Treatment of MCF‐7 and MDA‐MB‐231 breast cancer cells with PH (50 mg/mL) resulted in reduced cell survival, accompanied by elevated ROS levels, oxidative DNA damage, and apoptosis in a dose‐dependent fashion, suggesting its value as a potential nutraceutical or therapeutic agent. In parallel, Egyptian honey extracts were tested against Hep‐2 laryngeal cancer cells, where fennel honey exhibited the strongest antiproliferative effect (IC50 = 70 μg/mL), attributed to its high phenolic and flavonoid content. Multiflower honey also showed considerable cytotoxicity (IC50 = 87 μg/mL), whereas Sidr and clover honeys had weaker effects (IC50 = 100 and 130 μg/mL), consistent with their comparatively lower bioactive compound levels. The inverse correlation between phenolic/flavonoid content and IC50 suggests that the cytotoxicity of honey mainly stems from its antioxidant components (Das et al. 2022). Phenolic and flavonoid compounds are well‐established antioxidants with anticancer properties, acting through mechanisms such as induction of apoptosis, inhibition of proliferation, and generation of ROS leading to cancer cell death (Aumeeruddy et al. 2019; Arung et al. 2021). The higher phenolic and flavonoid concentrations in fennel and multiflower honey likely explain their stronger antiproliferative effects, whereas the lower levels in clover honey correspond to its weaker activity against Hep‐2 cells. This association highlights the role of bioactive compounds in honey's therapeutic potential and supports its function as a natural anticancer agent (Fauzi et al. 2011; Abel and Baird 2018).

### 2.2 Formation of Hydrogen Peroxide by Means of the Enzyme Glucose Oxidase, Low pH, Osmotic Pressure

Honey has played an active role against important pathogens due to its low water content, high sugar content, acidity, phytochemical components, non‐peroxide substances, peptides and hydrogen peroxide (H_2_O_2_). Honey has been involved in preventing microbial pathogenesis through pathways such as disrupting bacterial cell membrane integrity, inactivation through the oxidation process, osmotic pressure, and low pH (Almasaudi 2021). Yu et al. (2025) comprehensively examined the physicochemical properties, chemical composition, and antimicrobial activity of Scrophularia ningpoensis honey obtained from the Scrophularia ningpoensis plant used in traditional Chinese medicine. In addition, they examined the physicochemical properties, chemical composition, and antimicrobial activity of 8 different monofloral honey types ( *Brassica napus* honey [BNH], Euryahoney [EH], Triadica cochinchinensis honey [TCH], *Rhus chinensis* honey [RCH], Lithocarpus litseifolius honey [LLH], Castanopsis honey [CH], Manuka honey [MH] are also evaluated and compared for their antimicrobial effects). The antibacterial potential of SNH was assessed by determining its minimum inhibitory (MIC) and maximum bactericidal concentrations (MBC) through broth microdilution in 96‐well plates. A growth curve for *Klebsiella pneumoniae* exposed to SNH was generated, and its inhibitory effect was further examined using agar disk diffusion, where 50 μL of SNH at MIC, 0.5 MIC, and 0.25 MIC concentrations were applied to disks and evaluated after 24 h. The study also tested the biofilm resistance of *K. pneumoniae* against SNH. Results showed that SNH's fructose and glucose profile was consistent with mature honey and indicated low crystallization tendency. Its pH ranged between 2.59 and 3.83, lower than acacia and other monofloral honeys, highlighting its strong acidity, which is known to suppress microbial growth. Additionally, the dark coloration of SNH reflected a high phenolic content, supporting its potential for enhanced biological activity (Demir Kanbur et al. 2021). While honey's high sugar content contributes to antibacterial effects, the study emphasized that bioactive components also play a critical role (Jiang et al. 2022). Among 8 different honey samples, SNH was shown to have the highest antibacterial activity with the lowest MIC value. Consequently, it was proven to have the strongest inhibitory effect on bacterial spread with an initial ZOI measurement of 18.90 ± 3.62 mm. Furthermore, SNH was found to inhibit biofilm formation at three different concentrations, with the best biofilm inhibition result observed at its MIC concentration. Biofilm inhibition was calculated at a rate of 77.01% ± 3.92%. This study highlights the potential health benefits of Scrophularia ningpoensis honey, supported by modern scientific data, beyond its traditional uses, emphasizing the honey's physical and chemical properties. Its antimicrobial activity, in particular, suggests that this type of honey could be used as a natural antibacterial agent.

In another recent study, the physicochemical properties and antimicrobial activity of stingless bee honey from Malaysia were evaluated (Tiang et al. 2025). The physicochemical properties found (pH, sugar content, hydrogen peroxide presence) were shown to be directly proportional to the strength of SBH's antibacterial properties. Microbial inhibition is highly effective in the presence of low pH, high sugar content, and hydrogen peroxide. Furthermore, findings from some studies indicating that honey can exhibit antibacterial effects through other compounds such as organic acids, flavonoids, phenolic compounds, alkaloids, and terpenoids serve as evidence for the presence of non‐peroxide antibacterial components in stingless bee honey (Saputra et al. 2021; Wu, Han, et al. 2023). Additionally, honey creates high osmotic pressure due to its high sugar content; this prevents bacterial growth by drawing water from the wound surface, while the glucose oxidase enzyme it contains produces low levels of hydrogen peroxide (H_2_O_2_) in the wound environment, exhibiting both bacteriostatic and bactericidal effects against bacteria (Song and Salcido 2011; Combarros‐Fuertes et al. 2020). This provides an antimicrobial effect in the wound environment while also contributing to wound healing (Yupanqui Mieles et al. 2022; Oryan et al. 2016). The oxidative signal produced by H_2_O_2_ at low doses stimulates VEGF release from macrophages, promoting blood vessel development (angiogenesis) in the wound area (Scepankova et al. 2021).

Honey, which can provide a moist environment to the wound surface, prevents scab formation and supports keratinocyte migration and epithelialization (Yupanqui Mieles et al. 2022). With the high osmotic pressure it creates, honey draws lymph fluid to the wound area, facilitating autolytic debridement (removal) of dead tissue and reducing tissue damage during dressing changes (Scepankova et al. 2021). Honey contributes to wound healing by suppressing protease activity, which lowers pH and helps preserve growth factors and extracellular matrix (ECM) integrity—essential for tissue homeostasis and repair (Iosageanu et al. 2024; Naskar et al. 2024). This effect is attributed to honey's low pH, high osmotic pressure, and antioxidant compounds (flavonoids and phenolics), which together support fibroblast activity, collagen synthesis, and new tissue formation (Majtan 2014; Alvarez‐Suarez et al. 2014; Martinotti and Ranzato 2018). The acidic environment also enhances oxygen release from hemoglobin via the Bohr effect, further aiding tissue oxygenation (Minden‐Birkenmaier and Bowlin 2018). In a study by Martinotti et al. (2025) thirteen honey samples from Italy's Piedmont region were evaluated for wound healing properties. Parameters such as color intensity (Pfund scale), total phenolic content (TPC), total flavonoid content (TFC), H_2_O_2_ production, and wound closure rate (scratch assay) were analyzed. A positive correlation was observed between H_2_O_2_ production and wound closure rate, while other parameters showed no significant association. These findings highlight H_2_O_2_ as a key contributor to honey's wound‐healing potential. Another study investigated honeys from Java, Kalimantan (Borneo), and East Nusa Tenggara (NTT) in Indonesia (Eko et al. 2025) on *Staphylococcus aureus* ‐infected wounds in Balb/c mice. Skin wounds were treated with hydrocolloid dressings alone or combined with the regional honeys. Results suggested that honey application improved healing compared to control, underscoring the therapeutic potential of different honey varieties in infected wound management. In daily wound area ratio measurements, the NTT group showed the smallest wound area ratio, while the control group showed the largest ratio. Also, the NTT group had the lowest amount of fluid leakage, while the control group had the highest amount. Histopathological analyses showed that the group treated with NTT honey had higher rates of re‐epithelialization and collagen accumulation, indicating better outcomes. In conclusion, these findings highlight the benefits of honey in wound treatment, demonstrating that honey supports epithelialization and accelerates wound healing.

### 2.3 Modulation (Activation or Suppression) of Proinflammatory Pathways and Signal Transmission Pathways

The systemic biological effects of honey are primarily mediated through cellular signaling and the subtle modulation of pro‐inflammatory pathways. Honey samples rich in phenolic and flavonoid compounds modulate these pathways in a multifaceted manner: primarily by suppressing NF‐κB and p38/JNK‐MAPK activation and reducing pro‐inflammatory cytokine transcription such as TNF‐α, IL‐1β, and IL‐6. As a result of this effect (Megha et al. 2021; Ramírez Miranda et al. 2024; Stavropoulou et al. 2022), they are reported to shorten the inflammation phase by creating a tendency to shift the macrophage phenotype from M1 to reparative M2, which has been reported to accelerate wound closure (Liu et al. 2013; Liu et al. 2019; Rahmani and Babiker 2025; Talebi et al. 2020). The main signaling pathways governing wound healing are as follows: TLR4 (Toll‐like receptor that initiates inflammation by recognizing microbial structures), NF‐κB (the main transcription factor regulating proinflammatory gene transcription), MAPK (a cascade that converts cellular stress/stimuli into growth and cytokine production), PI3K/Akt (a fundamental signal for cell survival, proliferation, and angiogenesis), STAT3 (a cytokine‐derived proliferation and regeneration signal), and TGF‐β/SMAD (a regulator of fibrosis and matrix synthesis) (Bonnici et al. 2023; Guo et al. 2024). These pathways interact with each other to determine the severity of inflammation, macrophage phenotype balance, angiogenesis, and collagen production (Guo et al. 2024; Bezerra et al. 2023). Additionally, honey components stimulate the Keap1/Nrf2 axis, increasing HO‐1 and other antioxidant targets, thereby reducing ROS load (Süntar et al. 2021; Pleeging et al. 2022); this antioxidant effect reduces oxidative stress‐induced cellular damage and improves collagen synthesis and cell survival. This facilitates indirect support of the PI3K/Akt pathway, accelerates mechanical wound closure by promoting keratinocyte and fibroblast proliferation and epithelialization (Ranneh, Akim, et al. 2021; Alvarez‐Suarez et al. 2016; Yang et al. 2023). These mechanistic findings have correlated with increased keratinocyte migration, fibroblast collagen synthesis, and accelerated epithelialization in laboratory models (Chepulis and Francis 2012; Xin et al. 2015; Ebadi and Fazeli 2021). Furthermore, honey components have been shown to suppress TLR4‐mediated excessive LPS‐induced signaling, thereby limiting early‐stage excessive inflammation (Iosageanu et al. 2024; Kassim et al. 2010). On the other hand, some studies have reported that honey exhibits antiproliferative and proapoptotic effects by suppressing STAT3 phosphorylation and the AKT/mTOR axis in cancer cells; this points to honey's potential for both regenerative and selective anticancer effects (Márquez‐Garbán et al. 2024; Martinotti et al. 2024; Bose et al. 2024). Furthermore, the polyphenols and antioxidant properties of honey may help reduce oxidative stress and neuroinflammation in nervous tissue (Fadzil et al. 2023). Overproduction of ROS in neurons and activation of NF‐κB in microglia are key contributors to the development of neurodegenerative disorders, including Alzheimer's and Parkinson's diseases (Navarro‐Hortal et al. 2025). Honey extracts have been shown to increase Nrf2 activation and elevate glutathione levels, thereby making neurons more resistant to oxidative damage (Asari et al. 2019). Furthermore, in experimental models, honey has been reported to reduce pro‐inflammatory cytokines (IL‐6, TNF‐α) by suppressing the MAPK and NF‐κB pathways, thereby limiting microglial activation. Some researchers have suggested that honey application improves learning and memory performance (Azman et al. 2018); this effect may occur through both antioxidant protection and PI3K/Akt and BDNF signaling, which regulate synaptic plasticity (Zamri et al. 2023). In conclusion, the literature shows that phenol‐rich fractions of honey can produce wound‐healing, anti‐inflammatory, antioxidant, anticancer, and neuroprotective effects through NF‐κB/MAPK inhibition, enhanced Nrf2‐mediated antioxidant response, and a combination of PI3K/Akt‐controlled regenerative support. However, it is emphasized that the effects show heterogeneity depending on the type of honey, dose, and model system, and that more randomized, mechanistic studies are needed for translational validation (Bose et al. 2024; McLoone et al. 2024).

Honey exerts a protective effect on neural tissue, particularly by limiting oxidative stress and excessive inflammatory responses. It has been shown that honey reduces proinflammatory cytokine production, thereby balancing microglial activation. In addition, honey strengthens antioxidant defense mechanisms, reducing free radical‐induced neuronal damage and increasing the resilience of nerve cells. Furthermore, the support of neurotrophic factors contributes to the preservation of synaptic integrity and the maintenance of learning and memory functions.

These diverse mechanisms position honey as a promising candidate for neuroprotection (Fadzil et al. 2023; Navarro‐Hortal et al. 2025). Honey exhibits neuroprotective potential through multiple mechanisms, with stingless bee honey (SBH) showing particular promise. Rich in trehalulose, probiotics, organic acids, and bioactive compounds, SBH can modulate neurobiological factors such as brain‐derived neurotrophic factor (BDNF) and neurotransmitter release, while reducing oxidative stress and inflammation. In a chronic restraint stress (CRS) rat model, forty‐two mice treated with SBH or paroxetine demonstrated improved behavioral, hormonal, and histological outcomes, indicating that SBH may alleviate stress‐induced neuronal dysfunction and support brain health (Shaheran et al. 2025). Evaluation parameters were body weight, behavioral outcomes, neurotransmitter levels, hippocampal neuronal integrity, and BDNF expression. Physicochemical analysis verified the compliance of SBH with Malaysian Standard MS2683:2017, and field electron microscopy revealed the presence of bacteria and yeast in cerumen pots. The findings showed that SBH reduced anxiety‐like behavior and immobility, decreased corticosterone, maintained serotonin, and increased dopamine availability, partly via elevated phenylalanine as a dopamine precursor. Furthermore, SBH upregulated BDNF and downregulated pro‐inflammatory cytokines, indicating enhanced neurotrophic support by upregulating brain‐derived neurotrophic factor (BDNF), which promotes synaptic plasticity, neurogenesis, and neuronal survival and regulation of monoamine neurotransmission. In addition, SBH reduces neuroinflammation by downregulating pro‐inflammatory cytokines such as IL‐6 in the hippocampal CA3 and dentate gyrus regions, thereby preserving neuronal integrity. Histological analyses further confirmed its role in protecting hippocampal neurons from stress‐induced degeneration. All these results suggest that SBH provides neuroprotection through monoamine modulation, neurogenesis, and anti‐inflammatory pathways, contributes to overall brain health, and mechanistically supports its potential as a functional food with antidepressant effects that point to the necessity of further clinical validation. Nayan et al. (2020) investigated the impact of stingless bee honey (KH) on oxidative stress markers using an autism‐derived lymphoblastoid cell line (ALCL) as a model for autism spectrum disorder (ASD), alongside a control cell line (NALCL) derived from a healthy sibling. KH application (400 μg/mL, 24 h) significantly increased the activities of superoxide dismutase (SOD; an enzyme that neutralizes superoxide radicals) and glutathione peroxidase (GPx; an enzyme that detoxifies peroxides), which were low in ALCL; and DNA breaks, when assessed by the comet assay, showed a marked decrease in moderate‐to‐high damage (Grade 2–3) levels. In contrast, no significant changes were observed in catalase (CAT; an enzyme that breaks down H_2_O_2_) and malondialdehyde (MDA; an indicator of lipid peroxidation) levels. The findings suggest that KH alleviates DNA damage by enhancing antioxidant defense in ALCL cells and may be a potential nutraceutical agent in modulating oxidative stress associated with ASD. In the metabolic field, preclinical data (Chen et al. 2022) suggest that honey may support insulin signaling associated with IRS‐1/PI3K/Akt and positively affect glucose homeostasis; clinical studies report that balanced consumption does not worsen insulin resistance and may positively contribute to the lipid profile (McLoone et al. 2024; Ramli et al. 2018). Metabolic syndrome is defined as a complete set of five risk factors that increase susceptibility to type 2 diabetes (T2DM) and cardiovascular disease (CVD) (Alberti et al. 2009). It is characterized by the concurrent presence of glucose intolerance, hyperinsulinemia, hypertension, and dyslipidemia, indicating that these conditions co‐occur due to an underlying link rather than chance (Reaven 1988). Honey is a powerful and promising natural agent in the counteract of metabolic syndromes with its anti‐obesity, hypoglycemic, hypolipidemic, and antihypertensive effects (Erejuwa et al. 2012; Samat et al. 2017; Abdulrhman et al. 2011; Pai et al. 2018). These benefits are largely attributed to its polyphenol content, which can lead to the inhibition of lipogenic enzymes and, through synergistic activity, reduce weight gain and adipose tissue accumulation (Khitan and Kim 2013; Samat et al. 2019). Additionally, the antioxidant and anti‐inflammatory properties of polyphenols protect against endothelial dysfunction, thereby lowering the risk of hypertension (Sánchez et al. 2006). Despite being rich in carbohydrates, honey has been found to enhance insulin sensitivity and regulate glucose metabolism (Hashim et al. 2021). Ultimately, honey may serve as a valuable adjunct in preventing and managing metabolic syndrome, primarily by alleviating oxidative stress and inflammation. In a study, the objective is to evaluate chronic stress, a widespread health concern that contributes to metabolic disturbances (Ghasemi et al. 2025). Since natural honey has shown beneficial effects against stress‐related disorders, this study investigated the protective role of thyme honey in regulating blood glucose by assessing muscle GLUT4 protein expression in male rats subjected to chronic unpredictable mild stress (CUMS). For methodology, adult male Wistar rats were divided into six groups as a control group given only water, two non‐stressed groups administered honey (0.2 or 2 g/kg/day) for 38 days, a stressed group exposed to CUMS for 4 weeks; and two stressed groups treated with honey (0.2 or 2 g/kg/day) from 10 days before stress induction until the end of the stress period. On day 39, in a non‐fasting state, animals were sacrificed to evaluate serum glucose, insulin, irisin, lipid profile, and muscle GLUT4 protein using western blotting. After the experiment, CUMS elevated blood glucose, reduced serum irisin and HDL‐c, and downregulated GLUT4 expression. Honey treatment alleviated stress‐induced hyperglycemia, significantly increased serum irisin, and slightly raised HDL‐c, while not affecting other lipids or insulin. Importantly, high‐dose honey (2 g/kg) restored GLUT4 protein levels in stressed rats to near‐normal values. The findings of this study suggest that thyme honey helps preserve glycemic control under chronic stress by enhancing irisin production and stabilizing GLUT4 protein expression, thereby counteracting stress‐induced metabolic dysregulation. Song et al. (2024), reported that a polysaccharide fraction (AHPN80) isolated from Alhagi honey exerted protective effects against alcohol‐induced liver inflammation. Alcohol is known to trigger the TLR4/MAPK pathway, causing oxidative stress, proinflammatory cytokine release, and tissue injury; however, AHPN80 treatment suppressed TLR4 overactivation, reduced MAPK phosphorylation (ERK, JNK, p38), lowered oxidative stress markers, enhanced antioxidant defenses, and preserved liver structure. These findings indicate that AHPN80 may serve as a natural anti‐inflammatory agent with potential applications in functional foods or therapy. Similarly, Kim et al. (2024) investigated chestnut honey (CH) combined with cabbage (CB) for gastroprotection, highlighting the role of kynurenic acid, abundant in CH, as a potent anti‐inflammatory metabolite. This study examined whether the combination of CH with KA‐containing CH (KACH) and CB (CH + CB or KACH + CB) exhibited a synergistic effect in both LPS (lipopolysaccharide)‐stimulated RAW 264.7 macrophage cells and in an indomethacin (NSAID)‐induced gastritis model. The regulation of proinflammatory markers such as NO, iNOS, IL‐6, and TNF‐α and the NF‐κB (nuclear factor kappa B) signaling pathway in LPS‐stimulated macrophages was evaluated, as well as the expression of Nrf2, the main regulator of the antioxidant defense mechanism, and its targets, such as the enzymes HO‐1, SOD, GPx, and GST. Following the in vitro studies, Sprague–Dawley rats were administered different ratios (1:9, 1:1, 9:1) of CH + CB or KACH + CB combinations, followed by gastric injury induced with indomethacin and tissue damage was investigated. The findings demonstrated that proinflammatory cytokines (IL‐6, TNF‐α) were suppressed, NF‐κB activation was reduced, and antioxidant enzymes (HO‐1, SOD, GPx) were increased through the activation of the Nrf2 pathway. In the animal model, CH + CB and especially KA‐rich CH + CB mixtures significantly reduced gastric lesions. Consequently, these combinations were found to possess synergistic gastroprotective potential against NSAID‐induced gastric damage.

Phenolic and flavonoid compounds in honey reduce oxidative stress by limiting free radical formation and regulate cell cycle signaling, which can suppress cancer cell proliferation, activate apoptosis, and modulate inflammation in the tumor microenvironment. Altin‐Celik et al. (2025), demonstrated that Pervari honey (PH) decreased viability of SH‐SY5Y neuroblastoma cells in a dose‐ and time‐dependent manner, increased BAX and Caspase‐3, suppressed BCL‐2, and modulated NF‐κB and proinflammatory cytokines (IL‐1β, IL‐6, TNF‐α). Elevated MMP‐2 and MMP‐9 levels suggested a role in extracellular matrix remodeling, highlighting PH's antitumor and immunomodulatory potential. Similarly, Kim et al. (2025) investigated *Hovenia dulcis* honey (HH) on benign prostatic hyperplasia (BPH) in mice and DHT‐stimulated RWPE‐1 cells. HH reduced prostate enlargement, epithelial thickening, and AR target proteins (PSA, PCNA, DHT), while suppressing inflammation via inhibition of iNOS, COX‐2, TNF‐α, and IL‐6. These results suggest HH helps maintain prostate tissue homeostasis by limiting proliferative and fibrotic processes. Honey also protects against oxidative stress by inhibiting proinflammatory and stress pathways such as NF‐κB and MAPK, while enhancing antioxidant defenses via PI3K/Akt and Nrf2 pathways. Ranneh et al. (2019), reported that stingless bee honey (SBH) exerted anti‐inflammatory and antioxidant effects in a rat model of chronic subclinical inflammation induced by LPS. SBH significantly reduced serum levels of CRP, TNF‐α, IL‐1β, IL‐6, IL‐8, MCP‐1, and oxidative stress markers MDA and 8‐OHdG; it also enhanced antioxidant defense mechanisms such as GSH, GPx, and GST. Furthermore, it reduced NF‐κB p65 and p38 MAPK expression while increasing Nrf2 levels, thereby reversing histological and functional damage in liver, kidney, heart, and lung tissues; thus demonstrating that SBH has a protective potential in modulating CSSI and oxidative stress‐related damage. Similar researchers, the aim of Ranneh, Mahmoud, et al. (2021) evaluate the effect of stingless bee honey (SBH) in a Sprague–Dawley rat model of LPS‐induced acute systemic inflammation. Animals received 4.6 or 9.2 g/kg SBH for 7 days, followed by a single dose of LPS, and were assessed at 6 h. LPS increased multi‐organ damage, pro‐inflammatory cytokine elevation (e.g., TNF‐α, IL‐1β, IL‐6), ROS, lipid peroxidation, and oxidative DNA damage via NF‐κB p65/p38 MAPK/HMGB‐1 activation. SBH pretreatment significantly improved these parameters; it suppressed NF‐κB p65, p38 MAPK, and HMGB‐1 while increasing Nrf2 and glutathione, thereby reducing oxidative stress and tissue damage. The findings suggest that SBH is a promising nutraceutical candidate for modulating acute inflammation and oxidative stress.

A recently published study (Browne et al. 2025) investigated the antioxidant and immunomodulatory properties of Irish monofloral ivy ( *Hedera helix* ) and heather ( *Calluna vulgaris* ) honey, using PMA‐differentiated THP‐1 macrophages as a model. The main point of this study is the comparison of these honeys with manuka honey, a well‐studied medicinal honey, to understand their therapeutic potential. They assessed antioxidant activity via total phenolic content, radical scavenging assays (DPPH and ORAC), cytotoxicity, and the expression of key inflammatory and antioxidant genes (NF‐κB, TNF‐α, IL‐1β, IL‐10, Nrf2, and SOD). After experiments, results revealed that heather honey displayed the highest total phenolic content, surpassing even manuka honey, while ivy honey showed moderate levels. Both Irish honeys demonstrated antioxidant activity, although manuka honey exhibited superior radical scavenging capacity. Most importantly, ivy and heather honeys strongly induced Nrf2 activation and upregulated superoxide dismutase (SOD), indicating that their antioxidant effects were beyond direct radical scavenging and involved stimulation of intracellular defense pathways. In the immunological pathways, ivy honey primarily promoted pro‐inflammatory responses through NF‐κB and TNF‐α induction, while heather honey elicited a balanced response by upregulating both pro‐inflammatory cytokines (NF‐κB, TNF‐α, IL‐1β) and the anti‐inflammatory cytokine IL‐10. These activities suggest that heather honey may help fine‐tune immune responses, whereas ivy honey acts more as an immune activator. The therapeutic mechanisms behind these effects were largely attributed to the phenolic compounds of honey, particularly flavonoids, and other bioactive constituents. As a result, the findings of the study highlight ivy and heather honey as potential natural agents for oxidative stress regulation and immune modulation, with possible applications in inflammation‐related conditions, wound healing, and the equilibrium between antioxidants. Through these mechanisms, honey can reduce oxidative damage in cells and prevent the harm caused by inflammation. These effects provide important biological foundations that support honey's therapeutic potential, such as neuroprotective and anticancer properties.

Wound healing is a functional regenerative outcome involving the migration and proliferation of epithelial cells to re‐cover the damaged area of the epidermis, thereby restoring mechanical integrity; concurrently, it involves the regression of inflammatory cells, the maturation of granulation tissue, and the reorganization of the extracellular matrix (Oryan et al. 2016; Bonnici et al. 2023; Tang et al. 2024). Wound healing involves a complex interaction between many cell types, cytokines, mediators, and the vascular system. The process begins with the inflammatory phase, which involves the construction of blood vessels and the aggregation of platelets to stop bleeding (Beck et al. 2009). This is followed by the proliferative phase (Singh et al. 2017). Various inflammatory cells, primarily neutrophils, arrive at the site. These cells release mediators and cytokines that support angiogenesis (blood vessel formation), clotting, and re‐epithelialization (Ebadi and Fazeli 2021). Epithelial tissue reforms, a process that can take weeks. Finally, the maturation and remodeling phase: The wound reaches its maximum strength and matures over time with fibroblasts (Zhu et al. 2023; Eming et al. 2007). Clinical‐level meta‐analyses show that applying honey to superficial and partial‐thickness wounds significantly reduces treatment time, increases the rate of complete healing, and improves the sterilization of infected wounds; for example, statistically robust effects have been reported, such as wound healing time being on average ~4.6 days faster, complete healing rate RR ≈2.13, and rate of sterilizing infected wounds RR ≈9.08 (Michelle and Lorna 2018). A recent meta‐analysis on chronic wounds indicated that honey dressings shortened the average healing time by ~17 days, which is also a statistically significant effect (Tang et al. 2024). However, it reported that heterogeneity between studies (honey type, procedure, quality) altered the results; therefore, while the current evidence is promising, higher‐quality randomized controlled trials (RCTs) are needed. The study conducted by Alvarez‐Suarez et al. (2016) examined the effects of Manuka honey ( *Leptospermum scoparium* ) on human skin fibroblasts at the molecular level. The main objective of the study was to prevent damage caused by oxidative stress in fibroblasts by activating the AMPK (AMP‐activated protein kinase) and Nrf2 (nuclear factor erythroid 2‐related factor 2) signaling pathways, thereby preventing oxidative stress‐induced damage in fibroblasts and strengthening cellular defense mechanisms via antioxidant response elements (ARE), and thus supporting wound healing. In the study, intracellular reactive oxygen species (ROS) levels, mitochondrial function parameters, and antioxidant enzyme expressions were measured in human dermal fibroblast cells treated with Manuka honey. Study results demonstrated that Manuka honey enhances wound healing by activating the AMPK/Nrf2/ARE signaling pathway, decreasing ROS levels, improving mitochondrial function, and promoting fibroblast proliferation and migration. These effects suggest that Manuka honey may serve as a food‐derived therapeutic agent supporting tissue repair, especially in skin exposed to oxidative stress. Overall, the findings highlight its molecular mechanisms in wound healing and underscore the role of antioxidant activity and mitochondrial regulation. Additionally, a recent investigation on various Romanian honeys systematically assessed how their compositional characteristics relate to anti‐inflammatory and wound‐healing properties (Iosageanu et al. 2024). The study showed that honey types rich in phenolic compounds, in particular, significantly reduced the production of proinflammatory cytokines such as TNF‐α and IL‐8 in lipopolysaccharide‐stimulated macrophages in vitro models. This finding was interpreted as indicating honey's potential effect in regulating the inflammatory phase. At the same time, honey application in fibroblast cells increased collagen synthesis and cellular proliferation, while in the “scratch assay” test performed on keratinocytes, accelerated cell migration and re‐epithelialization were observed. Although signaling pathways were not directly analyzed in the study, the suppression of proinflammatory cytokine release indicates inhibition of NF‐κB activity, the increase in fibroblast functions indicates support of the PI3K/Akt axis, and the increase in collagen synthesis indicates modulation of the TGF‐β/SMAD pathway. In conclusion, this study demonstrates that honey plays a role in controlling inflammation and supporting regenerative cellular processes, particularly through phenolic compounds, thereby contributing to wound closure and repair processes at the biochemical level. Moreover, Gad et al. (2025) study evaluated the protective effects of garlic extract (AGE) and royal jelly (RH) against bisphenol A (BPA)‐induced gastric toxicity using in vitro and in vivo methods. In the study, BPA exhibited toxic effects, including marked cellular degeneration, erosion, inflammation, and increased apoptosis in the gastric mucosa. These effects were observed in histopathological examinations as disruption of mucosal integrity, structural losses in epithelial cells, and inflammatory infiltration. AGE and RH treatments significantly reduced this damage in the gastric mucosa; the morphology of epithelial cells was partially preserved, and the mucosal layer thickness and glandular structure approached normal levels. Immunohistochemical analyses showed a marked decrease in the expression of inflammatory and apoptosis markers such as TNF‐α and caspase‐3. These findings demonstrate that garlic and royal jelly support gastric tissue repair by modulating inflammation and cellular death processes. SEM analyses indicated a reduction in cell surface damage and microvillus loss in the treatment groups, suggesting structural improvement at the cellular level. The study authors interpreted that AGE and RH play a protective role against the toxic damage caused by BPA through both anti‐inflammatory and anti‐apoptotic mechanisms, and that this effect contributes to the preservation of gastric mucosal integrity and the repair of ulcer‐like lesions.

### 2.4 Hydrogels Combined With Honey

Hydrogel consists of three‐dimensional, cross‐linked polymer networks with high water content (Ahmed 2015; Lei et al. 2025). It is a fundamental component of modern wound healing due to its advantages such as moist wound healing, management of wound exudate, pain reduction, and biocompatibility (Ribeiro et al. 2024; Liang et al. 2024; Francesko et al. 2019) Honey‐hydrogel systems combine the osmotic effect of honey, its low pH, and its antimicrobial/antioxidant properties derived from hydrogen peroxide and phenolic compounds with the moist healing microenvironment provided by hydrogel. The honey‐hydrogel system is essentially created by mixing the honey to be used with natural or synthetic polymers (chitosan, gelatin, pectin, alginate, PVA/PVP) (Ahmed 2015) resulting in the formation of a three‐dimensional network through freeze–thaw (physical), ionic (alginate with Ca^2+^), chemical (e.g., genipin), or photochemical cross‐linking (Chin et al. 2024). Hydrogels designed in this way incorporate antimicrobial and antioxidant functions into the wound healing hydrogel system. Recent research has shown that hydrogel dressings accelerate epithelialization, particularly in chronic wounds, and that evidence of their clinical efficacy has strengthened (Lei et al. 2025; Narayanan and Bhaskar 2025) furthermore, it has been proven that honey‐containing hydrogels exhibit greater biological efficacy compared to other wound dressing systems due to their effects on water retention, reduction of bacterial infection, and support for cell proliferation and re‐epithelialization (Zainuddin et al. 2025). Currently, honey‐hydrogel‐based studies show positive results in chronic wounds, but long‐term and more specific research is still lacking (Hosseini et al. 2025). In summary, honey‐loaded hydrogel dressings represent a valuable and rapidly evolving wound repair design that accelerates the closure of acute and chronic skin wounds by providing a biocompatible, moist, and antimicrobial healing microenvironment, as described in the current literature (Zainuddin et al. 2025).

In a study conducted by Mahmod et al. (2025), the feasibility of using hydrogel films based on gellan gum (GG) enriched with acacia honey (SBH) for wound healing was investigated. Formulations containing 10%, 15%, and 20% (v/v) SBH (GGSBH10, GGSBH15, GGSBH20) were prepared. In physical evaluations, the water vapor transmission rate and swelling ratio of the GGSBH20 formulation were found to be optimal for improving the wound environment. Additionally, antibacterial tests revealed that the formulations were sensitive to *Escherichia coli* , which was considered an important finding in reducing the risk of infection. In cell culture tests conducted with 3T3‐L1 cells, analyses using the MTT and scratch assays revealed that none of the formulations exhibited cytotoxic effects up to 72 h, and they supported cell viability and proliferation. Notably, the GGSBH20 formulation demonstrated the highest cell migration and wound closure activity among the formulations. Therefore, it was interpreted as a promising candidate for a biocompatible and effective wound dressing. The findings support the argument that GG‐SBH hydrogel systems hold strong innovative potential for the development of innovative, biocompatible, and infection‐preventing dressing materials in wound treatment applications. In another study, Suhaimi et al. (2025) developed and characterized honey‐pectin hydrogel formulations with the aim of creating biocompatible and moisture‐managing wound dressing materials that facilitate wound healing. Hydrogel samples with varying honey concentrations prepared by the freeze–thaw method were imaged using scanning electron microscopy (SEM) and evaluated for chemical structure stability using Fourier transform infrared spectroscopy (FTIR). According to the results of SEM and FTIR analyses, honey was observed to increase the structural integrity of the hydrogel and that the honey content could have a positive effect. Furthermore, swelling tests performed in the study revealed that the hydrogel samples created could absorb moisture quite well in the wound environment and thus prevent the wound environment from drying out. In addition, it was shown that it could provide moisture management in the most ideal way according to the moisture release time. As a result of the study, the honey‐pectin hydrogel system was evaluated as innovative, customizable, and effective according to clinical wound treatment requirements, and it has the potential to be a highly effective wound dressing material. Additionally, Razif et al. (2025) aimed to develop a new biomaterial formulation specifically for skin lesions that take time to heal, such as diabetic wounds. They developed injectable hydrogel systems by incorporating Kelulut honey (KH) into a gelatin matrix cross‐linked with genipin. The hydrogels were stabilized with genipin by preparing gelatin and KH at different concentrations. Analyses revealed that the addition of KH significantly improved physicochemical parameters of the wound environment, such as swelling ratio, water vapor transmission rate, contact angle, porosity, enzymatic degradation, and surface roughness. The hydrogel system, which was observed to reach a level that supports the ideal moisture balance for the wound microenvironment, also has the potential to be a newly designed tissue‐wound repair material that can be administered by injection, with the biological activity of KH also improving properties such as mechanical strength, biocompatibility, and antimicrobial activity, thereby possessing the efficacy to prevent cutaneous tissue loss.

Thus far, honey exhibits potent antioxidant, antimicrobial, anti‐inflammatory, and immunomodulatory effects due to its phenolic compounds, flavonoids, enzymes, and other bioactive molecules. These multifaceted biological activities enable honey to be considered not only a natural sweetener but also a health‐promoting functional food. Current findings suggest that honey may offer beneficial effects across a wide range of areas, from cardiovascular health to metabolic balance, wound healing, and regulation of the gut microbiota. However, supporting these mechanisms with more comprehensive research at the molecular level is critical to fully understanding honey's potential therapeutic value.

## 3 Bioactive Constituents of Honey and Its Potential Health Outcomes

**FIGURE 4:**
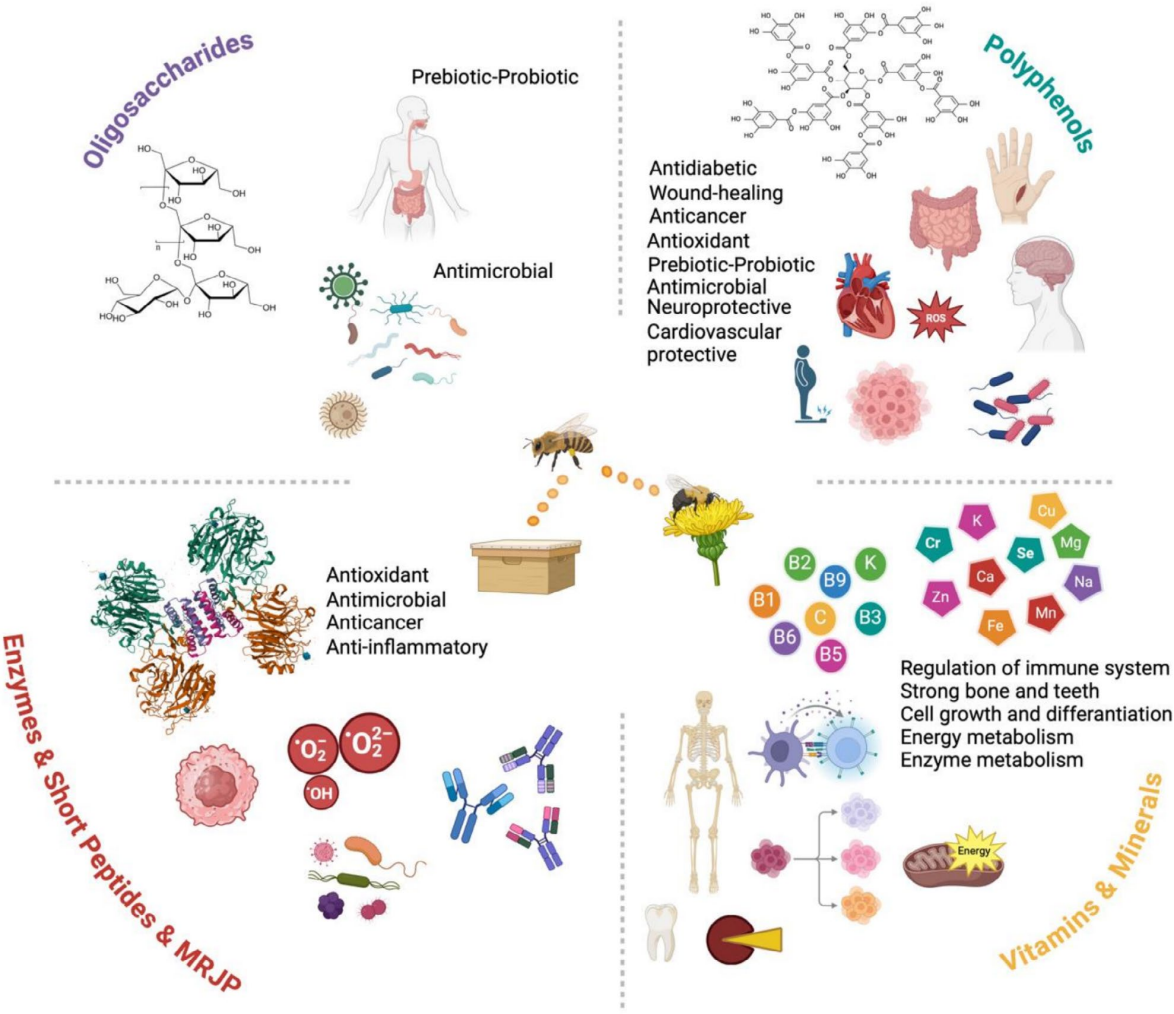
Bioactive constituents of honey and their health confirmation.

Honey, has been the subject of research from the past to the present and has shown health benefits that include antimicrobial, anticancer, neuroprotective, antioxidant, anti‐inflammatory, cardiovascular protective, wound‐healing and antidiabetic. These health benefits have been considered with the existence of bioactive constituents of honey (Al‐Kafaween et al. 2023). In this context, the evaluation of more than one therapeutic effect of the active components of honey has been of interest to researchers. In this direction, it has been investigated that honey is mostly composed of valuable oligosaccharides, enzymes, polyphenols, vitamins and minerals and therapeutic effects related to these bioactive components (Figure 4).

### 3.1 Oligosaccharides

Honey is made up of 55%–75% glucose and fructose, known as monosaccharides, while 10%–25% is a complex mixture of carbohydrates such as sucrose, maltose, turanose, and other oligosaccharides (Chen et al. 2022; Sanz et al. 2005). Oligosaccharides (OSs) are known as short‐chain carbohydrates with low molecular weight (Liu, Shen, et al. 2023), and were originally found in foods with characteristics such as sweetening and physiological effects on human health (De Gregorio et al. 2023; Karav et al. 2016) They are characterized as glycosides including 2–10 sugar moieties that can be different or the same type, joined by a glycosidic bond (Mano et al. 2022), demonstrating diversity from the points of sugar arrangement and polymerization degree (DP) (Duman et al. 2021). OSs can also be in linear or branched form (Kajiwara et al. 2002), and classified into the following 2 categories as digestible and non‐digestible monosaccharide polymers (Pekdemir et al. 2022). Digestible ones can easily be digested and absorbed by the human intestine's enzymes (hydrolytic enzymes in the GI) (Liu, Shen, et al. 2023; Şahutoğlu et al. 2020). Common digestible oligosaccharides are sucrose, maltose, etc. The other concept of non‐digestible oligosaccharides has a carbon (C) atom (C1 or C2) on monosaccharide units of configuration, which makes the glycosidic bonds between monosaccharide units unable to be broken down by the hydrolysis impact of human intestine enzymes and have crucial biological functions (De Gregorio et al. 2023). Non‐digestible oligosaccharides are also known as functional oligosaccharides including mannan oligosaccharides (MOS), chitosan oligosaccharides (COS), xylo‐oligosaccharides (XOS), fructo‐oligosaccharides (FOS), and galacto‐oligosaccharides (GOS) (Liu, Shen, et al. 2023; Pekdemir and Karav 2024). These undigestible oligosaccharides are claimed to be inducers for growing beneficial bacteria and provide prebiotic effects in the human gastrointestinal (GI) tract (Kajiwara et al. 2002). Generally, beneficial bacteria are associated with bifidobacteria and lactobacilli in the human gut, thus promoting these beneficial bacteria resulting in the stimulation of bacteriocins, which are good inhibitors of pathogenic bacteria (Kaplan et al. 2022). Additionally, these oligosaccharides can be fermented by beneficial bacteria, and as a result of fermentation, they form metabolites that include acetic acid and lactic acid, lowering the pH value of the intestine (Shin and Ustunol 2005; Karav 2018; Karav et al. 2019). Also, non‐digestible oligosaccharides undergo fermentation by native gut flora and can produce metabolites like short‐chain fatty acids (SCFA) (Gibson et al. 2010). In this way, it balances the intestinal flora and prevents the proliferation of unwanted bacteria, while helping to heal intestinal disorders such as constipation and diarrhea. It also stimulates the absorption of important minerals, including iron, calcium, and magnesium, and can minimize the risk of intestinal‐colon cancer (Nguyen and Haltrich 2013). It can also prevent colon cancer by inhibiting the transition of bile acids from primary into secondary form (Watanabe et al. 2008). In addition to these health‐promoting effects, they can stimulate immune function, reduce cholesterol, modulate lipid metabolism, and decrease the risk of cardiovascular diseases (Schell et al. 2022). Furthermore, non‐digestible oligosaccharides were demonstrated to decrease the risk of metabolic diseases that include insulin sensitivity and diabetes mellitus (Erejuwa et al. 2011). A research study evaluated gut microbiota's role in the metabolic profile of insulin‐resistant mice (Dumas et al. 2006), and other different studies demonstrated that microbiota have an exacerbated role in the immune system in people with challenged insulin resistance and obesity (Cani et al. 2008; Shi et al. 2006). Additionally, potential prebiotic oligosaccharides and antibacterial components of honey can stimulate and increase the probiotic impacts against pathogens in direct proportion to each other by inhibiting pathogen adhesion to intestinal epithelial cells (Mohan et al. 2017; Bunyatratchata et al. 2023). Owing to this property, researchers performed a study, which aimed to investigate potential honey ingredients managing the population of probiotic and infectious microbes. Manuka honey increased probiotic growth including *Lactobacillus rhamnosus* and *Bifidobacterium lactis* in the gut and decreased pathogens such as *Escherichia coli* and *Salmonella typhylococcus* aureus growth in a dose‐dependent manner (Rosendale et al. 2008). Furthermore, honey oligosaccharides were studied to investigate the impact of intestinal flora using an in vitro fermentation system. Impressions of three honey oligosaccharide fractions and honey samples were analyzed by comparison of FOS to investigate microbial fermentation. For this purpose, 1% (w/v) flora microbes in an in vitro fermentation system including 10 mg of carbohydrate and 1.0 mL of basal medium. After calculation of the prebiotic index (PI), the result showed that PI values of honey oligosaccharides' potential prebiotic activity range between 3.38 and 4.24 with enhancing the lactobacilli and bifidobacteria population; however, the PI value of FOS‐treated is 6.89, which possesses high prebiotic activity (Sanz et al. 2005). Additionally, two different types of honey, such as Malaysian and Tualang, were analyzed for their prebiotic effect and researchers observed that these types of honey can induce the growth of *Bifidobacterium longum* (Jan Mei et al. 2010). In these two different studies, researchers suggested that the activity of FOS provides selective stimulation of the growth of these bacteria and supports balanced gut microbiota formation and enhances the strength of gut microbiota against colonization of pathogens. Thanks to their mucosal carbohydrate‐binding properties, this resistance by healthy bacteria ensures that pathogens that include *Helicobacter pylori* , *Salmonella enterica* , *Campylobacter jejuni* , and *Escherichia coli* are blocked and thus prevent disease. This antiadhesive activity prevents toxins and pathogens from attaching to the intestinal cell surface by exerting an antiadhesive effect. Owing to this purpose, New Zealand Manuka honey was tested for its total oligosaccharide fraction to exhibit antiadhesive impacts for the prevention of the adhesive ability of *Escherichia coli* , *Cronobacter sakazakii* , *Listeria monocytogenes* , *Pseudomonas aeruginosa* , *Salmonella enterica serovar*, and *Typhimurium*. The human colonic HT‐29 cell line was used with the presence and absence of Manuka honey oligosaccharides. According to the results of the process, these oligosaccharides importantly decreased the adhesive binding ability of *Escherichia coli* by 40%, *Pseudomonas aeruginosa* by 52%, and *Staphylococcus aureus* by 30%. Researchers suggested that Manuka honey contains anti‐infective oligosaccharides for the destruction of bacterial populations and exhibited that these oligosaccharides can lower the percentage of the formation of infectious diseases (Lane et al. 2019). Moreover, a recent study aimed to assess the impact of Bifidobacterium‐derived postbiotics and GOS combinations on intestinal flora of patients with hidden sugar (Beteri et al. 2024). After 12 weeks of clinical trials, there was a reduction in diabetic symptoms and stimulation of helpful intestinal flora populations that include *Barnesiella*, *Eubacterium eligens* , and *Anaerostipes*. Additionally, researchers suggested this combination supports healthy intestinal flora and modulates the metabolism of the immune system and glucose. Although honey oligosaccharides have multiple health benefits, there is a need for further research to bring the literature up to date. Although the mentioned studies show that they protect intestinal health against pathogens, there is a deficiency in terms of evaluating the antimicrobial activity of specific oligosaccharides and bringing them to the literature.

### 3.2 Polyphenols

Natural plant‐derived secondary metabolites, polyphenols possess 500–4000 kDa molecular weight and are originally found in plant foods that include grains, fruits, vegetables, and beverages (Bolat et al. 2024). Also, polyphenols are natural phenolic compounds and have structural phenolic rings with multiple hydroxyl substituent groups. Depending on the number of these subcomponents, polyphenols can perform various biological interactions and demonstrate therapeutic impacts that include antibacterial, anti‐inflammatory, anticancer, antioxidant, and anti‐aging (Ganesan and Xu 2017; Sejbuk et al. 2024). The polyphenol composition in honey is influenced by the type of flower it is derived from, the geographical region and the climatic conditions of that region's bee species and can be used as a floral classification and authentication marker (Cianciosi et al. 2018). The most commonly identified phenolic compounds in honey are caffeic acid, gallic acid, p‐coumaric acid, vallinic acid, syringic acid, chlorogenic acid, 4‐(dimethylamino)benzoic acid, kaemferol, pinobanksin, quercetin, galangin, chrysin, luteolin, apigenin, pinocembrin, and pinobanksin (Cianciosi et al. 2018). These phenolic substances have been considered to have antioxidant ability of honey, and this activity is related to their ability to decrease ROS. Phenolic compounds decrease ROS by donating hydrogen from OH^−^ and the degree of reaction energy depends on the amount of OH of these compounds (Rice‐Evans and Miller 1996; Karav and Ekşi 2013). In the presence of these functions, polyphenols alleviate the distribution of chronic diseases and modify the treatment. Researchers have also evaluated that polyphenols are advantageous in the treatment of cardiovascular issues, metabolic health, and epistemic health (Bolat et al. 2024).

The antibacterial impact of honey is based on osmolality, low pH (3.4–6.1) and the fact that honeybees produce hydrogen peroxide by oxidation of glucose. However, the antibacterial effect of honey does not depend only on sugar constituents or the genesis of hydrogen peroxide (Kassym et al. 2024). Accordingly, Manuka honey shows strong antibacterial properties thanks to high amounts of flavonoids, phenolic acids and methylglycol (MGO) (Didaras et al. 2020). Accordingly, the research highlighted the broad‐spectrum antibacterial ability of 3‐phenyllactic acid (PLA) in Manuka honey (Mu et al. 2012). Another important compound of Manuka honey, p‐coumaric acid (PCA), shows antibacterial properties by disrupting cell walls of bacteria and interacting with their DNA (Boo 2019). Another compound, fluorethane, has shown antibiofilm and bacteriostatic effects against various pathogens: *Streptococcus pneumoniae* , *Moraxella catarrhalis* , *Haemophilus influenzae* , and *P. aeruginosa* (Birru et al. 2021). Fluorethane, which shows strong antibacterial activity for the destruction of *Cutibacterium acnes* populations compared to benzoyl peroxide, the standard acne treatment, shows both antibacterial and anti‐inflammatory properties by reducing toll‐like receptor‐2 (TLR‐2) induced, which is an innate immune receptor that has a role in defending microbial infection, bacterial inflammation (Kim et al. 2018). Additionally, a recent study suggested that phenolic constituents of honey may perform an antimicrobial impact thus aiming to demonstrate the sub‐inhibitory concentrations of phloretin, PLA, and PCA for decreasing the *Escherichia coli* ‐induced skin‐wound infection by in vitro assay with broth culture and disc‐well assays (Kassym et al. 2024). Because of the research, the most influential antimicrobial agent found was PLA, continued with PCA and the last one is phloretin, with a 2 mg/mL amount, allowing the proliferation of *Escherichia coli* . This study may provide valuable insight for the investigation of antimicrobial products targeting skin infections. The antibacterial effect of honey is also closely linked to wound healing. Honey is able to sterilize wounds, promote tissue regeneration, reduce edema and prevent scarring. These effects are beneficial for conditions such as burns, diabetic foot ulcers, pressure ulcers and simple wounds (Alvarez‐Suarez et al. 2016; Saha et al. 2012; Gouda et al. 2015). Honey has also been shown to reduce the population of Candida fungus infections by using an agar disk‐diffusion test and Minimum Inhibitory Concentration (MIC) assay (Irish et al. 2006; Moussa et al. 2012).

Currently, natural products are being used more frequently in the prevention of different health issues, including cancer, metabolic syndromes, digestive diseases, cardiovascular diseases, and epistemic (Karav et al. 2015). In addition, they play a crucial role in the treatment of various dermatological health issues. Among these, acne is a common problem in individuals (Koch et al. 2024). Acne vulgaris particularly affects young people and occurs in 35%–90% of adolescents (Melnik 2015). For the treatment of acne, antibiotic applications are often used; however, this has allowed the occurrence of resistance. This resistance is supported by biofilm formation, the spread of resistant strains, and genetic mutations. In contrast, plant extracts containing phenolic compounds show promise against acne‐causing bacteria (Koch et al. 2024). A research study was conducted to demonstrate the antibacterial effects of honey on acne vulgaris induced by *Staphylococcus epidermidis* (Abdulreda et al. 2023). 100 *Staphylococcus epidermidis* isolates from the face, neck, and upper trunk were collected from patients aged 15–30 years with acne infections. The isolates collected were highly resistant to more than one antibiotic. However, honey was shown to be a potential natural product for the treatment of acne, showing a high sensitivity of 100%. Additionally, the ethanolic extract of cinnamon bark with honey was also tested for its antibacterial activity for the destruction of the population of *Propionibacterium acnes* and *Staphylococcus epidermidis* (Julianti et al. 2017). In the study, minimum bactericidal concentration (MBC) and MIC values against *P. acnes* and *S. epidermidis* were determined by the disk diffusion method. As a consequence of the study, the MIC values of cinnamon bark extract were 256 and 1024 μg/mL against *P. acnes* and *S. epidermidis* , respectively, while the MIC value of honey was 50% v/v for both *P. acnes* and *S. epidermidis* . The antibacterial effect of the combination of cinnamon extract and honey was shown as a 0.625 value. Thus, they support the decreasing population of bacteria‐caused acne. Moreover, the total phenolic content of honey prevents UVB damage on human keratinocytes (Karapetsas et al. 2020). In this study, the photoprotective impacts and anti‐aging effects of Greek honey samples from different origins were investigated. As a consequence of the study, the protective role of honey extracts provided cell viability by reducing UVB exposure‐induced DNA damage and the increase of matrix metalloproteinases (MMPs). In conclusion, honey extracts have photoprotective and anti‐aging potential against UVB radiation and can be used in natural cosmetic products.

Furthermore, researchers have long been studying the anticancer and antitumor effects of phenolic compounds (Prieto et al. 2019; Diab et al. 2021). Research shows that polyphenols can inhibit the genesis of tumors and prevent the formation of cancer thanks to their antiproliferative, antioxidant, and anti‐inflammatory ability (Bolat et al. 2024). In an in vitro study, honey extracts obtained from three different sources were tested on breast cancer cells (MCF‐7) and evaluated for their effectiveness in regulating the estrogenic viability and activity of honey samples (Tsiapara et al. 2009). For the purpose of the study, MCF‐7 cells were exposed to honey extract concentrations between 0.2 and 125 μg/mL for 48 h. As a result, they presented in the literature that honey samples have a biphasic effect, that is, they act as an estrogen and an antiestrogenic agent based on the high and low concentrations of it. It was found that thyme and pine honey extracts inhibit the activity of estrogen in the existence of estradiol. The study also observed the impacts of three different honey extracts on cell viability. It has been determined that thyme and pine honey have no impact on the viability of MCF‐7 cells, but fir honey increases cell viability. In line with these observations, researchers have suggested that the impacts of these honey extracts are because of the existence of phenolic contents, which include quercetin and kaempferol. Moreover, a recent study sought to assess the antitumor activity of manuka honey on MCF‐7 cells (Márquez‐Garbán et al. 2024). Researchers demonstrated that both in vitro and in vivo experiments presented the antitumor effect of manuka honey in the literature. Different concentrations of manuka honey allow an important dose‐dependent suppression of MCF‐7 proliferation. Also, researchers found that the administration of manuka honey significantly prevented the genesis of MCF‐7 tumors and tumor volume in mice. Since phenolic compounds of manuka honey are crucial for potential chemoprevention or antitumor efficacy. In a different study, the cytotoxic effects of manuka, buckwheat, and acacia honey varieties containing different amounts of polyphenolic compounds on the A431 cancer cell line were investigated. The study revealed that manuka honey had the most significant effect on intracellular ROS and calcium balance, with disruption of this balance leading to apoptosis and the death of cancer cells. This is the first evidence that manuka honey can cause an increase in ROS and calcium imbalance in cancer cells. In addition, it was interpreted that the high H_2_O_2_ permeability of manuka honey strengthens this mechanism, and the apoptosis caused by H_2_O_2_ by opening the aquaporin‐3 channel provides the anti‐cancer effect (Martinotti et al. 2020).

Additionally, there is research showing that honey possesses positive effects on diabetes mellitus. Measurement of glycosylated hemoglobin, fructosamine, and glucose to control glycemic issues in diabetic patients is commonly done and is a basic practice (Rohlfing et al. 2002). An in vivo research study on the effect of honey on diabetes has been performed in rats. Consumption of honey supplement could alleviate the concentration of glucose; thus, it can reduce hyperglycemia in male Wistar rats (Adesoji and Oluwakemi 2010). The hypoglycemic and antidiabetic impacts of honey have also been associated with its antioxidant capacity, which combats ROS, although the development of diabetes may have multiple causes (Caturano et al. 2023). The increase in ROS concentration is directly proportional to the increase in adipose tissue and glucose absorption by the muscles (Noh and King 2007). This negatively affects glucose utilization and glycogen production metabolism. In addition, it also disrupts insulin signaling metabolism, the process of pathways involved in this metabolism, changes insulin release by beta cells in the pancreas and disrupts the course of cell functions (Han 2016). However, it has been proven that the honey antioxidant impact can reduce the amount of ROS formed in the pancreatic organ and reduce the stress in the environment (Erejuwa et al. 2010). Diabetes leads to hepatic problems and kidney dysfunction by damaging both the liver and kidneys. Honey is helpful for the reduction of glucose levels in the blood and decreasing complications of diabetes; thus, a recent study was performed to investigate the efficiency of administration of stingless bee honey (SBH) on the kidney and liver of streptocin‐induced diabetic rats (Mohd Nasir et al. 2024). Rats were administered 2.0 g/kg SBH or metformin for 12 days. According to the result of the analysis, in SBH treatment group was observed reduced liver damage. Untreated groups exhibited increased glucose levels and liver damage. Moreover, a similar recent study also showed that the antidiabetic effect of Egyptian Sidr against streptozotocin‐induced diabetic rats (El‐Aarag et al. 2024). For the aim of the study, the activity of nitric oxide (NO) and malonaldehyde (MDA) was measured in pancreatic and hepatic tissues. The results showed that Sidr honey was able to lower high blood glucose and fructosamine levels. It also decreased NO and MDA levels and regulated antioxidant enzyme activity and Sidr honey can control the formation of ROS, hyperglycemia, caspased‐mediated cell death and antioxidant enzymes in diabetic rats. The health effects of polyphenols are wide‐ranging. An inflammatory vascular disease, atherosclerosis (AS), is caused by major mechanisms including oxidative stress, unregulated lipid metabolism, and inflammation (Libby 2002). Research suggests that phenolic constituents and probiotic relations have potential healing character for atherosclerosis (AS) health issues by improving inflammation, lipid situation, and ROS and also managing the intestinal flora population (Cruz Neto et al. 2024). Thus, honey was known as modulator for immune response in the development of AS with different blocking pathways of proinflammatory markers that include cyclooxygenase‐2 (COX‐2), C‐reactive protein (CRP), tumor necrosis factor‐alpha (TNF‐α), and ROS generation (Nguyen et al. 2019). Researchers reported that phenolic compounds and other minor components of honey can lead to this anti‐inflammatory effect (Kassim et al. 2010; van den Berg et al. 2008). One study examined the impacts of addition of flavonoid supplements to antihypertensive therapy in hypertensive patients on leptin, blood pressure, lipid profile, CRP levels, and body mass index. Results showed decrease in total cholesterol, systolic and diastolic blood pressure, low‐density lipoprotein (LDL) cholesterol, and triglyceride levels. The reduction in CRP levels may also indicate an alleviation in the occurrence of cardiovascular issues (de Jesús Romero‐Prado et al. 2015). It has been suggested that the occurrence of cardiovascular issues is related to the consumption of foods enriched with certain compounds that include vitamin C and flavonoids, the types of polyphenols found in honey. There are several mechanisms of flavonoids to protect heart health that include (1) alleviating blood platelet activity, (2) prevention of oxidation of LDL, (3) increasing vasodilation of coronary, (4) increase of high‐density lipoprotein (HDL), and (5) improvement of endothelial function (Khalil and Sulaiman 2010). An old study demonstrated the healing effects of natural honey on the lipid profile of patients and according to the study result, serum triacylglycerol, total cholesterol, CRP, and LDL are decreased in the obese participants. In obese individuals, where the risk of cardiovascular problems is high, honey has also reduced the symptoms that can adversely affect heart health (Yaghoobi et al. 2008). Moreover, a recent randomized clinical trial was performed to investigate the impacts of the supplementation of honey and compared it with lipid profile and sucrose. As a consequence of the study, compared with the consumption of sucrose, honey potentially decreased total cholesterol and LDL, while enhancing HDL and triglycerol (Rasad et al. 2018).

Polyphenols are thought to have a potential neuroprotective role against neurodegenerative diseases because of their antioxidant and anti‐inflammatory properties. In diseases such as Alzheimer's and Parkinson's, the potential of polyphenols to reduce oxidative stress and protect neurons by scavenging free radicals is of great interest (Bolat et al. 2024). Many individuals are challenged with Parkinson's disease due to oxidative and inflammatory cascades (Eker et al. 2023). Preventing oxidative and inflammatory causes may therefore be the answer to Parkinson's disease progression. To this end, researchers have investigated the therapeutic side of honey and levodopa against 1‐methyl‐4‐phenyl‐1,2,3,6‐tetrahydropyridine (MPTP) induced oxidative stress in a Parkinson's disease model. According to the results of the study, higher motor activities in the Parkinson's disease model were observed with the honey‐levodopa treatment. Also, the honey‐levodopa treatment protected the midbrain from MPTP‐induced chromatolysis, and the antioxidant markers such as glutathione expression increased (Sulaimon et al. 2024b). Alzheimer's disease, which is also widespread worldwide, such as Parkinson's, has been shown to suppress symptoms and improve the course of the disease thanks to the antioxidant impacts of honey polyphenols. In different in vivo and in vitro studies, chestnut and kelulut honey have been shown to be neuroprotective in the improvement of Alzheimer's. In these studies, it was concluded that kelulut honey can prevent the occurrence of Alzheimer's disease by decreasing amyloid plaque accumulation and p‐tau levels. In addition, according to the study, these effects of kelulut honey have been associated with its high polyphenolic content. This neuroprotective effect presents the literature that kelulut honey has the effect of reducing oxidative stress and neuroinflammation (Shaikh et al. 2024). According to the analyses performed in the chestnut honey study, it has been shown that there is an improvement in learning ability. By examining the histopathological findings, researchers have proven that chestnut honey significantly reduces neuronal loss. The results show that chestnut honey, which can be taken daily, alleviates the symptoms of Alzheimer's disease by preventing memory breakdown and brain changes (Djebli et al. 2024).

Polyphenol‐rich foods have been found to support health through their antioxidant, anti‐inflammatory, and many other properties. Furthermore, these properties are considered to improve the microbial population of the gut (Sejbuk et al. 2024). These benefits support the consumption of polyphenol‐rich foods (Bolat et al. 2024). Accordingly, the consumption of honey polyphenols has the potential to improve gut health. Polyphenols can regulate the activity of gut microbiota and modulate the colonic microbial population; thus, polyphenols possess a healing impact on such inflammatory bowel diseases (IBD) (Cardona et al. 2013). Diseases such as Crohn's disease and ulcerative colitis are multifactorial IBD that occur as a result of a dysfunctional epithelium, innate and adaptive immunity to intestinal microorganisms. In such conditions, the intestinal mucosa is disrupted by the strong autoimmune and inflammatory effects, resulting in the mucosa lacking a protective barrier and absorptive properties (Shapiro et al. 2007). Furthermore, a research study aimed that honey polyphenols on DSS‐induced ulcerative colitis in a rat model (Zhao et al. 2019). The impacts of different components of honey were tested on DSS‐induced colitis in rats. Rats were administered honey polyphenols for 1 week, then colon and blood samples from rats were collected for gene analysis and understanding of gut microbial composition. As a consequence of the study, the colonic apoptosis is reduced, and also the expression of inflammatory cytokines of the gut immune system, including TNFα, IL‐1β, and IL‐6, is downregulated. Additionally, honey polyphenols decrease the population of *Bacteriodes*, *Corynebacterium*, and *Protus* species, which cause inflammation. Also, researchers suggested that the regulation of gene expression was performed with the impact of honey polyphenols and have reported that it is associated with the main types of gut microbiota. Honey polyphenols improve intestinal health by exhibiting antioxidant, anti‐inflammatory, and modulatory roles.

### 3.3 Enzymes and Short Peptides and Major Rolay Jelly Proteins

In honey samples, the content of protein is usually at a low concentration approximately 0.1%–0.5% in the form of free amino acids and enzymes, making protein extraction difficult (Chua et al. 2013). Normally, the number of free amino acids found is approximately 10–200 mg in 100 g of honey, and proline amino acid makes up most of the free amino acids (50%) content (Iglesias et al. 2004). The amount of protein is influenced by the origin of the honey bees (Won et al. 2009) and the knowledge of honey proteins, even in small concentrations, is important for the marker of pharmacological potential and authentications of honey (Soares et al. 2017; Afrox and Tanvir 2016). Proteins of honey may have bioactive markers for pharmacological impacts including antimicrobial, antioxidant, anti‐cancer, and anti‐inflammatory (Bardy et al. 2012; Gomes et al. 2010; Yusof et al. 2007). Honey contains various active enzymes under the class of proteins. There are 4 main possible sources of these enzymes: microorganisms in honey, plant‐sucking insects, plant nectar, and secretions or honeybees (Alvarez‐Suarez et al. 2013). Multiple enzymes such as glucosylceramidase, catalase, α‐glucosidase, α‐amylase, β‐glucosidase, proteases, especially intervase, glucose oxidase, and diastase, are present in honey (Rossano et al. 2012). With these enzymes, enzyme‐catalyzed and non‐enzyme‐catalyzed biochemical reactions take place in honey, and enzyme‐catalyzed reactions influence the quality and biomedical activities of honey.

Enzymatic reactions such as the transition of sucrose and maltose (oligosaccharides and disaccharides) into glucose and fructose are performed by intervase and diastase enzyme activity (Alaerjani et al. 2022). Invertase is involved in the breakdown of sucrose in honey into its subcomponents, while a trace amount of sucrose remains during the final stages of honey ripening (Ball 2007). The presence of glucose oxidase enzyme in honey inhibits potential microbial growth in honey. In addition, the glucose oxidase enzyme breaks down glucose into valuable hydrogen peroxide and gluconic acid (Kwakman and Zaat 2012). Hydrogen peroxide is also disassociated into oxygen (O) and water by catalase enzymes, which suggests that the high catalase activity of honey samples has low concentrations of hydrogen peroxide (Nolan et al. 2019). A specific reduction of the antibacterial property after combined treatment with honey and catalase affirms that hydrogen peroxide is a crucial antibacterial compound (Brudzynski et al. 2012). Furthermore, glucose oxidase enzyme activity is the dominant mechanism for the production of hydrogen peroxide and brings in honey biological activities such as bacteriostatic and bactericidal (Brudzynski et al. 2012, 2011). In this respect, the genesis and reduction of hydrogen peroxide are correlated with honey's antibacterial properties. A research study was conducted to show impacts of antibacterial impacts of hydrogen peroxide within the Canadian honey (Brudzynski 2006). This study determined hydrogen peroxide levels could perform antibacterial activity related to biosign, which allows the evaluating biomedical effect of honey. With broth microdilution assays, researchers analyzed the antibacterial impacts of different Canadian honeys for decreasing the population of *Escherichia coli* and *Bacillus subtilis* . The effect of hydrogen peroxide on antibacterial properties was evaluated by estimating the numbers of hydrogen peroxide before and after interaction with catalase and in line with this, the results were associated with the antibacterial effect. According to the results of the study, whole Canadian honeys exhibited moderate to severe antibacterial effects against both *Escherichia coli* and *Bacillus subtilis* . In this study, the removal of hydrogen peroxide by the enzyme catalase was found to reduce the antibacterial activity of honey, but the enzyme could not completely degrade the endogenous hydrogen peroxide in honey. The numbers of produced hydrogen peroxide in honey are meaningfully related to the MBC and MIC (Brudzynski et al. 2011). Most of the antibacterial impacts of honey have been considered to low ambient pH values because of concentrations of hydrogen peroxide, high sugar value and the presence of organic acids in honey (Alaerjani et al. 2022).

Additionally, honey samples with high numbers of hydrogen peroxide are valuable for the treatment of wounds. A recent research study aimed to demonstrate property‐wound healing relationships of Manuka, Anzer, and Chestnut honey and cell culture applications (Ünlü et al. 2024). In this study, the wound healing parameters of Turkish chestnut and Anzer honey were compared with Manuka honey. Chestnut honey contained as much as 5 μg/mL hydrogen peroxide, whereas Manuka and Anzer honey did not. Chestnut honey also had the highest acidity (24.50 meq/kg) and higher antioxidant activity (IC50: 2.45 mg/mL). Moreover, chestnut honey showed a significant antibacterial effect against the selected pathogens. The scratch test in the wound healing model showed that Anzer honey induced the highest cell proliferation and almost complete wound healing at all tested concentrations. The results suggest that Turkish chestnut and Anzer honey have comparable properties to Manuka honey in terms of wound healing and are promising candidates for clinical applications.

Furthermore, enzymatic reactions in honey also include protease enzyme interactions. Proteases have a degradation function to disassociate proteins into short peptides and amino acids according to the type of protease (Rawlings 2020). According to the substrate they use, proteases are categorized into two groups including endopeptidases (trypsin, chymotrypsin, and elastase) and exopeptidases (Rossano et al. 2012). The short peptides are major products of honey protease, and they have more than one biomedical property including antimicrobials, antioxidants, antitumor, and controller for weight loss (Alaerjani et al. 2022). In a recently completed research study, the existence of short and cyclic peptides in Acacia and Ziziphus honey was investigated, and the peptide identities of the honey were investigated (ALaerjani et al. 2021). Two Acacia honey samples and three Ziziphus honey were evaluated for their content of short and cyclic peptides using LC–MS. Moreover, the total protein concentration was quantified using the Bradford assay. The researchers proposed that the short peptides in honey may vary depending on floral origin and storage conditions. Due to the amino acid chain in short peptides, they have their biomedical properties (Alaerjani et al. 2022).

Different amino acids have distinct functions, such as hydrophobic amino acids glycine, valine, leucine, and alanine, which are active as antimicrobial; other amino acids like cysteine, tyrosine, lysine, tryptophan, histidine, and methionine act as antioxidants (Karami and Akbari‐adergani 2019; Kuroda and Caputo 2013). A recent study determined that the antioxidant activity of short peptides of Saudi honey and the detection of short peptides from different honey samples were performed with the LC/MS technique (Alarjani and Mohammed 2024). Furthermore, in this study, the antioxidant impacts were tested with the 2,2 diphenyl‐1 picrylhydrazyl (DPPH) capturing assay. According to the results of the analysis, the antioxidant capacity and effect of short peptides increased in direct proportion to the amounts of short peptides and proteins; thus, researchers suggested that amino acid chains characterize short peptides as antidiabetic, antioxidant, antimicrobial, and other biomedical properties.

Moreover, generally the origin of honey protein from nectar, flower pollen, but also some honeybee secretions from their cephalic gland can originate the honey proteins (Rossano et al. 2012). Thus, royal jelly proteins are secreted from the honeybee's gland. Additively, royal jelly proteins demonstrated an antimicrobial, antitumor, anti‐inflammatory, and antioxidant role. Most important are nine different major royal jelly proteins (MRJPs) that are degradable by proteases into short peptides. MRJPs are rich in special amino acids that include histidine, arginine, isoleucine, leucine, methionine, lysine, phenylalanine, valine, threonine, and tryptophan (Beltekin 2022). These are crucial for protein synthesis and many other biological processes (Ramanathan et al. 2018). Among MRJPs, classes 1–8 are evaluated as potential antioxidants (Guo et al. 2009), MRPJ1 is promotes liver regeneration (Jozef Simúth Some Properties of the Main Protein of Honeybee (Apis Mellifera) Royal Jelly 2001) and MRJP3 can show a modulatory role on human immune response (Okamoto et al. 2003). MRJP1, also known as royalactin, is reported to be an important protein for feeding queen bees and development through an Egfr‐mediated signaling pathway (Kamakura 2011). Although there are limited studies on protein identification in honey, a recent study evaluating the antibiofilm effects of the bee product honey on colon cancer‐associated *Escherichia coli* biofilm and its anticancer activities on the HCT116 cell line was carried out. This study evaluated the apitherapeutic effects of bee products, including honey, RJ, perga, pollen, propolis, and bee venom (Akkoyunlu and Dülger 2024). The antibiofilm activity of bee products was evaluated with the microplate assay by using crystal violet staining. The analysis result showed that honey and royal jelly inhibited the biofilm formation of *Escherichia coli* by more than 50%. Additionally, with the presence of the HCT116 colon cancer cell line, the antiproliferation effect of honey was determined with the water‐soluble tetrazolium salt‐1 assay. For this assay, 50% concentrated honey treatment was applied during the 48 h, and after treatment application, researchers reported that cell proliferation of colon cancer cells was reduced by 86.51%. In a recent study, MJRPs were observed to improve the immune system in immunocompromised mice by increasing white blood cell counts and enhancing certain immune responses. The effect of these proteins has also been associated with an increase in beneficial bacteria in the gut. These data suggest that MRJPs take a crucial role in the modulation of the immune system and could be used in the development of new immune‐boosting products in the future (Wang, Li, Li, et al. 2023). Moreover, in another study, researchers determined the antimicrobial and antioxidant roles of MRJP2 using recombinant AcMRJP2 derived from infected cells of insects. In this study, AcMRJP2 was shown to bind to bacterial and fungal surfaces and cause structural damage to microbial cell walls. It was also observed that AcMRJP2 directly protects the cell against oxidative stress and causes a decrease in caspase‐3 activity. In addition, AcMRJP2 was found to have DNA protective activity against reactive oxygen species (Park et al. 2019).

### 3.4 Vitamins and Minerals

Honey composition mainly consists of carbohydrates and water, however honey also comprises various vitamins and minerals. Vitamins of honey are thiamine (B1), riboflavin (B2), niacin (B3), panthothenic acid (B5), pyridoxine (B6), follic acid (B9), ascorbic acid (C), and phyllochinon (K) (Ajibola et al. 2012). These are mostly water‐soluble vitamins and although they are structurally different organic compounds, have common beneficial health effects such as sustaining metabolic differantiation, cell growth status and development, and energy metabolism (Hasam et al. 2020). Vitamin contents vary in honey according to floral origin, geographical conditions and bee species (Barreiros et al. 2024). Vitamin C is most found and evaluated in honey as its antioxidant activity (Barreiros et al. 2024; León‐Ruiz et al. 2013). In addition to vitamins, there are some minerals as micro and macro elements. Minerals in honey, like vitamins, vary from 0.1% to 0.2% of the total content of honey, depending on, botanical origin, geographical origin and analytical methods such as extractions (Barreiros et al. 2024). Antibacterial activity, one of the important properties of honey, is very limited against biofilm‐embedded bacteria. In a study conducted in this direction, the potential of vitamin C to potentiate the antibacterial effect of honey was evaluated in planktonic and biofilm‐embedded bacteria (Majtan et al. 2020). Vitamin C was combined with 4 different kinds of honey such as acacia, sunflower, linden, and honeydew and antibacterial activity was calculated by MIC concentration. Antibacterial activity, one of the important properties of honey, is very limited against biofilm‐embedded bacteria. In a study conducted in this direction, the potential of vitamin C to potentiate the antibacterial effect of honey was evaluated in planktonic and biofilm‐embedded bacteria. Vitamin C was combined with 4 different kinds of honey such as acacia, sunflower, linden, and honeydew and antibacterial activity was calculated by MIC concentration. Researchers demonstrated the antibacterial impacts of honeys combined with vitamin C against *Pseudomonas aeruginosa* was greatly increased. It has also been shown that in a wound biofilm model, 100% honey extract and 100 mg/g of vitamin C can completely destroy almost the entire bacterial population, including *S. aureus* . Moreover, honey alone showed no activity against *Enterococcus faecalis* bacteria, while the combination of vitamin C and honey showed some antibacterial activity against *Enterococcus faecalis* bacteria. Lead is a known environmental toxicant and also an element that causes dyslipidemia after oxidative stress injury. Numerous therapeutic potentials of the honey + Vitamin C combination can prevent the formation of ROS and dyslipidemia through exposure to lead acetate in Wistar rats (Adeyomoye et al. 2022). After 28 days of oral supplementation, rats were shown to have a reduced risk of dyslipidaemia, which can lead to metabolic diseases. Moreover a different animal model study was aimed to show the antinociceptive and antioxidative impacts of the Tualang honey + vitamin C combination and their alone form on the inflammatory pain of rat models (Hasim et al. 2020). As a consequence of the study, Tualang honey showed greater antinociceptive and antioxidative impacts when compared to vitamin C. Additionally, researchers also demonstrated honey with vitamin C supplementation exhibited wound repair in male albino mice (Sirhandi et al. 2024).

Minerals, which are sodium (Na), potassium (K), calcium (Ca), magnesium (Mg), phosphorus (P), copper (Cu), selenium (Se), iron (Fe), manganese (Mn), chromium (Cr), and zinc (Zn), possess crucial roles such as growth of strong bones, structure of teeth, structure of many enzymes, regulation of immune system, and healthy‐lengthy life (Gharibzahedi and Jafari 2017). Minerals most commonly found in honey are K, Mg, Ca, and P (Guiné et al. 2022). Also, considering honey color, a study demonstrated that the dark color of honey is because of the higher levels of minerals (Vanhanen et al. 2011). Researchers evaluated the generation of ROS and antibacterial activity of honey because of its phenolic and mineral contents (Faúndez et al. 2023). In this study, it was determined that the mineral, hydrogen peroxide, and phenolic contents of Chilean honey were analogous to those of honey from other regions. While all honey accumulated OH radicals, southern Chilean honey produced four times more OH radicals than northern Chilean honey. This difference was attributed to the higher total flavonoid content in southern‐originated honey. The relationships between phenolic‐mineral composition, ROS production, and antibacterial impacts of honey were presented in the study. Fe and Mn supported ROS production by triggering the auto‐oxidation of flavonoids and a Fenton‐like reaction. It was stated that quercetin, Cu, and Zn showed limited antioxidant effects because of their complex formation. Hydrogen peroxide and total phenolic content (TPC) support antibacterial activity especially against *S. epidermidis* . However, *P. aeruginosa* is less susceptible to ROS action since it is more resistant to oxidative stress. With these results, researchers showed the key role of phenolic composition, Fe, and Mn in ROS production in the antibacterial activity of honey. In addition, mineral concentrations of different honey samples, which originated from Croatia, were evaluated by researchers (Tlak Gajger et al. 2024). This study emphasizes that honey is an important detector of environmental quality due to the intensive feeding activity of bees. Co, Fe, Cr, Cu, Pb, Mn, and Zn levels were analyzed in meadow, acacia, chestnut, and comb honey samples taken from three different regions. Fe and Cu were observed in the highest concentration in the chestnut honey in the Varaždin region, in meadow honey Co, and in comb honey, Pb. The least concentrations of Pb were observed in meadow honey and the least concentration of Co was in comb honey in the Sisak‐Moslavina region. While specific discrepancies were evaluated in the concentrations of Mn, Cr, Fe, and Cu between kinds of honey, no discrepancy was observed in the concentrations of Pb Co, and Zn. With this study, the researchers proved that mineral concentrations in honey samples vary according to the type of flower and geographical location. Apart from this, according to the research carried out on honey mineral profile in soil type, positive correlations were found between significant kinds of soil and some elements: Na and K with phaeozem soil; Mn with ranker; Al with regosol; and Zn and Pb with anthrosol. Also, negative correlations were found between phaeozem and Mg, B, Al, Ca, Mn, Fe, Cr, Ni, Zn; Mg, Cr with regosol; and Al with rendzinas (Schmidlová et al. 2006).

## 4 Therapeutic Effects of Honey

Honey has strong antioxidant, anti‐inflammatory, and anticancer properties thanks to its phenolic acid content. While it shows antimicrobial activity with the enzymes it contains along with its phenolic content, it lowers glucose and glycosylated hemoglobin levels in diabetes treatment. It provides protective effects on the nervous, cardiovascular, respiratory, and gastrointestinal systems. It has been the subject of current research in the literature that the varying content ratios of distinct honey types have variable effects on their role in different health and diseases. Within this context, current studies examining the health benefits of different honey types are included (Table 1).

**TABLE 1:** Recent studies demonstrate the ability of honey to be a natural therapeutic agent.

Effects	Sample	Outcome	References
Antimicrobial	Spanish honeys	– Exhibited antibacterial activity against Staphylococcus epidermidis.	(Núñez‐Gómez et al. 2024)
	Trigona stingless bee honey Stinging bee *Centaurea hyalolepsis* *Citrus* honeys	– Exhibited antimicrobial activity against five different bacterial stains and two fungal strains – Biofilm mass was degraded.	(Aburayyan et al. 2024)
	Chestnut honey	– Antibiotic resistance biofilm was inhibited, and bacterial growth was suppressed.	(Koloh et al. 2024)
	12 Different Honeys	– Exhibited antimicrobial and cytotoxic properties of honeys against various bacterial strains	(Mohammed et al. 2024)
	Western Australian and Manuka honeys	– The antifungal effect was observed against six different clinical yeast – The osmotic activity of honey reduced the fungal strain population.	(Haines et al. 2024)
	Natural honey	– Bacterial strains of urinary tract infection were reduced	(Alain Prudence et al. 2024)
	Stingless bee (Meliponulla baccaerii) honey	– Drug resistance to human pathogenic microbes was reduced	(Jilo 2024)
	Manuka honey	– Exhibited antibacterial impact against antimicrobial‐resistant and extensively drug‐resistant *Salmonella typhi*	(Bashir et al. 2024)
	*Fallopia japonica* plants and honey	– Reduced biofilm including seven bacterial strains.	(Cucu et al. 2024)
	Brazilian organic honey	– Exhibited antibacterial effect against oral microorganisms	(Romário‐Silva et al. 2024)
	*Apis mellifera* bees honey	– Exhibited antibacterial activity against Salmonella in Southern Ethiopia	(Wolde and Mahamed 2024)
	Clover honey	– Exhibited antibiofilm activity against *Candida* species	(Masfufatun et al. 2024)
Anti‐cancer	Apis dorsata honey	– Exhibit cytotoxic effect	(Dalet et al. 2023)
	Monofloral honey samples	– Stimulate programmed cell death and regulates CK production in Caco‐2 cells	(Eisa et al. 2024)
	Manuka honey	– Exert inhibitory impact on various cancer cell types	(Idriss et al. 2024)
	Manuka honey	– Reduce in vitro growth of cell MCF7 cells in a concentration‐dependent manner and promotes programmed cell death through PARP activation	(Márquez‐Garbán et al. 2024)
	Palestinian honey samples	– Show notable cytostatic activity against MDA breast cancer cells and – Decrease cell viability	(Abu‐Farich et al. 2024)
	Yemeni Sidr honey	– Restricts the growth, survival, and motility of B16‐BL6, MDA‐MB‐231, MCF‐7, and HeLacancer cells	(Almnayan and Lafrenie 2024)
	Bee products (honey, pollen, perga, propolis, RJ, and bee venom)	– Inhibit biofilm formation and reduce colon cancer cell proliferation	(Akkoyunlu and Dülger 2024)
	Citrus bee honey	– Show strong antagonistic effects against clinical pathogens and reduce cancer cell viability	(Elsayed et al. 2024)
	Ziziphus jujube honey and commercial honey	– Effectively suppress tumor development	(Karbasi et al. 2024)
	Manuka honey and Saudi's honey	– Prevent the growth of cancer cells	(Alhawiti et al. 2024)
	Stingless bee honey	– Show strong antimicrobial, antioxidant, and antiproliferative activities	(Damayanti et al. 2024)
	Honey	– Reduce the prevalence of severe mucositis, relieve pains efficiently, and contribute to reducing weight loss in patients due to radiotherapy.	(Li et al. 2024)
	Coniferous honeydew honey	– Effectively inhibit breast cancer cell migration while sparing normal fibroblasts.	(Dżugan et al. 2024)
	Apis dorsata honey	– Upregulate CYP2J2 and CYP1B1, increase CASP8 expression, and decrease BCL2.	(Narag et al. 2023)
	Saudi Sidr honey	– Suppressess expansion, triggers apoptosis, and ceases cell cycle progression in cancer cells.	(Qanash et al. 2023)
	Longan honey	– Inhibit HEp‐2 cells.	(Ode Sumarlin et al. 2023)
	Honey	– Show anticancer effects.	(Ode Sumarlin et al. 2023)
	Honey samples (nigella sativa, moringa, sidr, pumpkin)	– Show anticancer activity against breast and colon cancer cells.	(Al‐Eisa et al. 2023)
	Monofloral honey with bee products	– Show apoptotic effects.	(Sánchez‐Martín et al. 2023)
	Forest honey	– Exhibit significant anticancer activity, against hepatocellular carcinoma and colon cancer cells.	(Ghramh et al. 2023)
	Acacia honey‐derived bioactive compounds	– Exert cytotoxic effect against breast cancer.	(Hamadou et al. 2023)
Anti‐inflammatory	Hovenia dulcis Thunb. monofloral honey	– Suppress inflammatory mediators, and reduce mitochondrial stress and proinflammatory gene expression.	(Hasitha Maduranga Karunarathne et al. 2024)
	Centauri honey	– Reduce inflammation and enhance immune responses.	(Santos Filipe et al. 2024)
	Manuka honey	– Reduce NET formation.	(Main et al. 2024)
	Alhagi honey oligosaccharide	– Improve antioxidant enzyme activity, reduce oxidative stress markers, and lower liver enzyme levels	(Lv et al. 2024)
	Thym honey	– Demonstrate superior effects	(Assaggaf et al. 2024)
	Honey	– Reduce edema	(Ramírez Miranda et al. 2024)
	Korean chestnut honey	– Reduce inflammatory cytokine levels and downregulate NLRP3 inflammasome expression	(Kwon et al. 2023)
	Polyphenols from Lespedeza bicolor Turcz. honey	– Downregulate pro‐inflammatory mediators like COX‐2, IL‐10, TNF‐α, and iNOS while enhancing HO‐1 expression.	(Ren et al. 2023)
	Apis mellifera honey	– Exhibit strong anti‐inflammatory activity	(Shahid et al. 2023)
	Medical grade honey	– Exhibit anti‐inflammatory properties	(Holubová et al. 2023)
	Citrus honeys	– Exhibit anti‐inflammatory properties	(Tel‐Çayan et al. 2023)
	Turkish sunflower honeys	– Show anti‐inflammatory effects targeting cyclooxygenase enzyme‐1 and cyclooxygenase enzyme‐2.	(Emin Duru et al. 2023)
Anti‐oxidant	Saudi honey	– Exhibit antioxidant activity – Correlates protein and short peptide content with antioxidant activity	(Alarjani and Mohammed 2024)
	Jara honey	– Exhibit significant levels of antioxidant activity	(Abashidze et al. 2024)
	Manuka honey	– Exhibit radical scavenging activity	(Kaźmierczak‐Barańska and Karwowski 2024)
	Unifloral honey	– Exhibit antioxidant activity	(Saftić Martinović et al. 2024)
	Honey	– Concludes the key factors determining antioxidant levels of plants	(Shakoori et al. 2024)
	Stingless bee honey	– Exhibit significant levels of antioxidant activity	(Mello dos Santos et al. 2024)
	Monofloral honey samples	– Reveal a notable positive association between antioxidant activity and electrical conductivity.	(Tananaki et al. 2024)
	Alhagi honey oligosaccharide	– Produce a therapetuic impact on mice with liver fibrosis induced by CCI4.	(Lv et al. 2024)
	Iranian‐originated honey	– Exhibit antioxidant activity	(Hajian‐Tilaki et al. 2024)
	Clover honeys	– Exhibit versatile activities including antioxidant	(Sultana et al. 2024)
	Algerian honeys	– Exhibit antioxidant activity correlated with phenolic compounds	(Harbane et al. 2024)
	Rapeseed honey	– Exhibit higher antioxidant activity when enriched with honey products	(Derewiaka et al. 2024)
	Moroccan honeys	– Exhibit strong antioxidant activity	(Lakhmili et al. 2024)
	South African Fynbos honey	– Exhibit variable antioxidant activities – Possess anti‐inflammatory effects	(Magoshi et al. 2023)
	Herbal honey	– Enhance the antioxidant capacity of chosen premium herbal honey – Expand storage duration – Reduce total bacterial load – Maintain the integrity of selected premium herbal honey	(Wahyono et al. 2024)
	Saudi Sidr honey	– Demonstrate antibacterial activity – Show activity against *Candida auris* and *Candida neoformans* – Exhibit antioxidant activities – Inhibit biofilm formation	(Bazaid et al. 2023)
	Honey	– Exhibit antioxidant properties	(Mduda et al. 2023)
	Blossom honey	– Rank antioxidant activity and phenolic content as propolis > bee pollen > honey	(Saroğlu et al. 2023)
	French Guiana honey	– Analyze TPC and anti‐radical potential in comparison with multi‐floral honeys from adjacent regions.	(Jiang et al. 2023)
	Honey	– Observe higher antioxidant activity in post‐feeding honey samples compared to pre‐feeding sample – Detect significant antibacterial activity in post‐feeding honey	(Kumari et al. 2023)
	Spanish oak honeydew honey	– Identify six biomarkers to distinguish Spanish oak honey – Establish correlations between individual phenolic compounds – Link antioxidant activity to phenolic compounds	(Hernanz et al. 2023)
	Unifloral honey	– B. ceiba exhibits the strongest antioxidant activity	(Wu, Zhao, et al. 2023)
	Chestnut (Castanea sativa) honey‐mediated silver nanoparticles	– Inhibit rates of myeloperoxidase and collagenase – Reveal antibacterial activity targeting both Gram staining‐positive and Gram staining‐negative bacteria.	(Keskin et al. 2023)
	*Eurya* honey	– Exhibit antioxidant activity – Identify phenolic and bioactive compounds	(Liu, Yao, et al. 2023)
	Honey	– Exhibit variable antioxidant activities – Demonstrate antimicrobial activity	(Zapata‐Vahos et al. 2023)
	Honey	– Exhibit antioxidant activity – Correlate phenolic content with antioxidant activity	(Tahirovića et al. 2023)
	Avocado (Persea americana Mill.) honey	– Demonstrate high antioxidant activity – Prevent ROS accumulation – Prevent the progression of Alzheimer's disease	(Romero‐Márquez et al. 2023)
	*Citrus* monofloral honey	– Exhibit variable antioxidant activity – Preserve bovine serum albumin against denaturation – Inhibit biofilm formation	(Fratianni, Amato, et al. 2023)
	Western Australian honey	– Exhibit significant antioxidant activity – Characterize the role of distinct phenolic compounds	(Lawag et al. 2023)
Prebiotic Effect	Pine honey	– Enhance the viability of probiotics namely B. animalis *subsp. lactis*	(Ayaz et al. 2024)
	Honey	– Possess antimicrobial and prebiotic effects supporting the growth of several probiotics	(Nazzaro et al. 2024)
	Honeybee brood biopeptides	– Exhibit prebiotic effects on certain species and strong antioxidant activity	(Ounjaijean et al. 2024)
	Honey	– Resist stomach acidity and digestive enzymes – Enhance the prebiotic effects of Bifidobacterium spp. and lactic acid bacteria – Increase in SCFA production	(Kowalska et al. 2024)
	Honey	– Enhance the growth of probiotics – Exhibit antioxidative effect – Demonstrate anti‐biofilm effects	(Fratianni, De Giulio, et al. 2023)
Neuroprotective Effect	Chestnut Honey	– Exhibit antioxidative effect – Protective effect against cognitive dysfunction	(Jeong et al. 2024)
	Tualang honey	– Decrease TNF‐α levels and caspase‐3 activity	(Hasim et al. 2024)
	Kelulut (Stingless Bee) honey	– Decrease amyloid plaque accumulation – Exhibit antioxidative effect	(Shaikh et al. 2024)
	Honey	– Modulate changes in motor coordination – Exhibit antioxidative effect	(Sulaimon et al. 2024a)
	Leguminous honey	– Exhibit antioxidant activity	(Fratianni et al. 2024)
	Chestnut honey	– Exhibit antioxidative effect – Decrease neuroinflammation	(Terzo et al. 2023)
Anti‐diabetic	Sidr honey	– Regulate blood glucose and fructosamine levels – Modulate antioxidant enzyme activity	(El‐Aarag et al. 2024)
	Stingless bee honey	– Regulate glucose levels and levels of enzymes indicating liver damage – Mitigate kidney damage	(Mohd Nasir et al. 2024)
	Green honey	– Regulate blood sugar levels – Exhibit antioxidant activity	(Huyop et al. 2024)
	Bitter honey	– Modulate cholesterol levels – Inhibit alpha‐amylase and alpha‐glucosidase enzymes	(Koodathil et al. 2023)
	Manuka honey	– Modulate hyperglycemia, hyperinsulinemia, and oxidative stress	(Iftikhar et al. 2023)
	Honey proteins	– Inhibit ROS production – Modulate expression of inflammatory markers	(Naqvi et al. 2023)
	*Devdarvadyarishta* honey	– Modulate hyperglycemia – Exhibit antioxidant activity	(Anti‐Diabetic and Anti‐Oxidant Activities of Devdarvadyarishta in StreptozotocinInduced Diabetic Rats 2023)
Wound healing and skin health	Medical grade honey	– Exhibited antibacterial effect and reduced bacterial infection. – Promote keratinocyte proliferation.	(Boekema et al. 2024)
	*Argania Spinosa* honey	– Exhibited antibacterial effect and reduced bacterial infection. – Healing surgical wounds.	(Khattabi et al. 2023)
	*Manuka* Honey	– Exhibited antibacterial effect and reduced bacterial infection. – Moist wound area.	(Gandini et al. 2024)
	Honey	– Antibacterial healing demonstrated.	(Kassym et al. 2024)
	Unprocessed Pasteurized *Manuka* Honey	– Topical application of honey reduces pathogens on the skin and promotes inflammatory response of the skin.	(Lemmen et al. 2024)
	*Manuka* honey	– Gene expression associated with tissue regeneration and human dermal fibroblast was increased. – Proliferation of bacterial population inhibited	(Gallo et al. 2024)
	*Sumatera Forest* honey	– Reduced size of wounds	(Darwis et al. 2024)
	*Mexican* honey	– Reduced edema and increased granulation tissue.	(Ramírez Miranda et al. 2024)
	Eucryphia cordifolia (Ulmo honey)	– Supported collagen and fibroblast maturation – Reduced bacterial population.	(Salvo et al. 2023)
	*Bacopa* Honey	– Decreasing biofilm population on the skin by activating the immune system of skin cells.	(Li et al. 2023)
	Honey	– Wound improved	(Sutrisno 2024)
	Medical grade honey	– Reduced wound size. – Exhibited antimicrobial impact on skin.	(Dzani and Nair 2024)
	Honey	– Exhibited antifungal activity on skin cells. – Reduced wound size	(Ifeoma et al. 2024)
	Honey	– Mimic skin tissue for feasibility on the treatment of diabetic wounds	(Dhiman et al. 2024)
	Honey	– Improve the duration of wound healing. – Exhibited acceleration of the healing process of the skin tissues	(Suryadinata et al. 2024)

### 4.1 Antimicrobial

Generally, honey is nowadays a natural therapeutic mixture used in the treatment of multiple diseases. The antimicrobial property of honey, most widely documented health benefits of honey, has shown an active role against important pathogens (Hossain et al. 2022). Additionally, the antimicrobial activity of honey is affected by various factors, including low water content, high sugar content (resulting in high viscosity), acidity, phytochemical components, hydrogen peroxide (H_2_O_2_), non‐peroxide substances, and peptides (Almasaudi 2021). The water activity (unbound water) in honey is low to prevent the microbial proliferation (Mavric et al. 2008). In addition, honey, with its high concentration of sugars, can induce osmosis in living cells, and therefore, since undiluted honey is hypertonic, if the water in the bacteria moves out of the cell, the bacterial cell dies. In this way, it can eliminate bacterial growth (Molan 1992). Subsequently, the pH range suitable for the growth of bacteria is usually in the range of 6.5–7.5, while the pH value of honey is highly acidic (pH 3.2–4.5) (Albaridi 2019). H_2_O_2_ is a strong oxidizing element formed as a result of the reaction of glucose oxidase enzyme activated in diluted honey on glucose (Molan 1992). It helps to inactivate bacteria through the oxidation process (Peter and Indelicato 2018). In addition, honey, with its phenolic acid and flavonoid content as non‐peroxide components, can show this property (Albaridi 2019). Honey varieties that have an antimicrobial effect with parameters such as these have contributed a lot of data to the literature in this context.

Among studies recent study aimed to exhibit Spanish honeys from various botanical origins for antibacterial activity against *Staphylococcus epidermidis* , which can be related to wound infection (Núñez‐Gómez et al. 2024). This bacterial strain is the most commonly found in human skin membranes and has the potential to cause wound infection. According to the result of MIC analysis, rosemary honey demonstrated antibacterial activity against *Staphylococcus epidermidis* by inhibiting of growth of the bacterial strain. These results highlight the antimicrobial properties of honey and for future research therapeutic usage of honey for potential applications in wound healing is also highlighted.

A recent study, focused on the characterizing the biofilm‐inhibiting and antimicrobial properties of Trigona stingless bee honey, comparing its effects with those of stinging bee Centaurea hyalolepsis and Citrus honey (Aburayyan et al. 2024). The study was conducted with five different bacterial stains, such as *Pseudomonas aeruginosa* , *Streptococcus pyogenes* , *Staphylococcus aureus* , *Escherichia coli* , *Klebsiella pneumoniae* , and two fungal strains, *Candida albicans* , and *Candida krusei*. Trigona honey was shown to exhibit a broader spectrum of antimicrobial activity against bacterial and fungal strains compared to the other two honey types. It was proven to show activity against microbial strains with a significant difference in the inhibition zone area from 9 to 25 mm and MIC between 9.4% and 29.6% (w/v). It was also found that the addition of honey to the biofilms formed caused a reduction effect on the szie of biofilm, which endows the usage of the Trigona honey in the industry of antimicrobial and antifungal.

In another recent study similar to this study, the potential effect of chestnut honey on mixed biofilm was investigated (Koloh et al. 2024). Because of the formation of biofilm or antibiotic resistance, suppression of bacterial strain growth becomes difficult and can cause many chronic infections. Therefore, researchers suggested that chestnut honey can be an alternative therapeutic agent and performed an antibacterial activity assay for bacteria such as *Pseudomonas aeruginosa* , methicillin‐resistant *Staphylococcus aureus* , and *Staphylococcus epidermidis* , which are recognized for forming multi‐species biofilm structures. All bacterial stains are exposed with chestnut honey for different maturity stages and different durations, as 2, 4, 6, 12, and 24 h. The biofilm inhibition was evaluated with scanning electron microscopy (SEM) and methicillin‐resistant *Staphylococcus aureus* , and *Staphylococcus epidermidis* were most sensitive with a 93.5% growth inhibition after 2 h of incubation. As a consequence of this study, chestnut honey is proper for preventing the growth stages of mixed biofilms.

Additionally, a complex study is performed to show the antimicrobial effect of 12 different honey from different regions (Mohammed et al. 2024). For the antimicrobial and antifungal study experiment, 4 bacterial strains such as *Bacillus subtilis* , *Staphylococcus aureus* , *Escherichia coli* , and *Pseudomonas aeruginosa* , and 2 fungi strains such as Aspergillus niger and *Candida albicans* were used. For antimicrobial processes, all bacterial stains are incubated at 37°C for 18 h but for fungal strain 25°C for 2–3 days. Although honey samples showed different antibacterial properties from each other, they generally showed the most antibacterial properties against *Escherichia coli* . Also, all honey species studied demonstrated similar antifungal activity against *Candida albicans* and Aspergillus niger. As a consequence of this study, Cucurbita and Ziziphus honey possess precious antimicrobial activity.

A different in vitro study aimed to show Western Australian and Manuka honey's effect against clinically serious yeasts (Haines et al. 2024). 12 Australian and 2 Manuka honey were selected for this research. Six clinical yeast isolates such as *Candida albicans* , *Candida parapsilosis* , *Nakaseomyces glabratus*, *Pichia kudriavzevii*, *Saccharomyces cerevisiae* , and *Trichosporon asahii* obtained from a laboratory service. As a consequence of the study, Australian honey exhibited antifungal activity against Candida species. In addition, researchers suggested that osmotic activity plays an important role in the inhibition of the growth of fungi species and the results of the study provided crucial preclinical data and potential therapeutic compound for candidiasis.

In addition to this study, bacterial strains are isolated from urinary tract infections among patients from a hospital (Alain Prudence et al. 2024). The bacteria that cause this infection have been isolated and in descending order, species such as *S. aureus* , *P. aeruginosa* , CoNS, *Escherichia coli* , K. pneumonia, and Streptococcus spp. have been obtained. All bacterial species subsequently inoculated into plates and incubated at 37°C for 24 h. Different bacteria possess classical antibiotic resistance, but natural honey samples showed that bacteria that are isolated from the urinary tract can be controlled. However, further studies are needed to show the effects of honey on the living cell.

A stingless bee (Meliponulla baccaerii) honey was tested for its antibacterial activity against *Salmonella typhi* , *Escherichia coli* , *Staphylococcus aureus* , and *Enterococcus faecalis* (Jilo 2024). Different concentrations (100%, 75%, 50%, and 25%) of stingless be honey were prepared and all bacterial stains were inoculated into plates and incubated at 37°C for 48 and 72 h then a zone of inhibition was measured. As a consequence of this study, these bacteria strains possess MIC of 25% and this research demonstrated antibacterial activity against all tested strains. Researchers suggested that this type of honey can be a potential agent against antibiotic‐resistant strains by controlling bacterial growth.

An in vitro study investigated the antimicrobial activity of manuka honey against multi‐drug‐resistant *Salmonella typhi* , which is causing septicemia in Pakistan (Bashir et al. 2024). Honey from the flowers of Manuka trees in Australia and New Zealand has shown antibacterial activity against this bacterial strain whose DNA is resistant to commonly used antibiotic drugs (XDR and MDR). According to the results of the analysis, honey at 100% concentration showed inhibition zones of 15–24 mm (MDR resistant strain) and 15–23 mm (XDR resistant strain). Moreover, honey tested at a concentration of 3.1% destroyed most of the bacterial strains resistant to XDR. With these results, the researchers suggested that manuka honey may be a potential therapeutic option for the treatment of infections caused by this bacterium after further in vivo or human analysis.

Researchers studied to demonstrate the antibiofilm and antibacterial activity of *Fallopia japonica* (FJ) plants and honey against 7 bacterial strains such as *Staphylococcus aureus* , *Enterococcus faecalis* , *Escherichia coli* , *Pseudomonas aeruginosa* , *Bacillus cereus* , *Salmonella enteritidis* , and the yeast *Candida albicans* (Cucu et al. 2024). The inhibitory analysis was tested with a crystal violet staining test. According to the result of the study, especially against *Escherichia coli* demonstrated the most antibacterial activity and FJ plant extract and honey exhibited high antibiofilm effect against *Staphylococcus aureus* and *Escherichia coli* , while the lowest inhibition was detected in *Enterococcus faecalis* and Candida yeast. According to the results, honey from this FJ plant may be a potential antibacterial agent in Gram‐positive and Gram‐negative bacteria.

Another research study aimed to evaluate the antimicrobial activity of Brazilian honey against oral microorganisms, which is the biofilm of *Streptococcus mutans* . Organic honey possess subgroups as OH at 1–8 position of carbon atoms, diluted and filtered. Antimicrobial analysis was performed with the MIC and CBM analysis as a catalyze assay. These honey samples exhibited principal antimicrobial activity especially, OH‐1, OH‐2, OH‐3, and OH‐7, with MIC values of these OH positions changing between 10 to 25%. Also, these OH positions demonstrated total biofilm decreases at basal concentrations of honey samples. As a consequence of this study, researchers suggested that these honey samples are potential inhibition agents to control the growth of oral microorganisms (Romário‐Silva et al. 2024).

Another recent study demonstrated *Apis mellifera* bees honey possesses antibacterial activity against Salmonella in Southern Ethiopia, and also compared with the antibacterial activity of garlic extract (Wolde and Mahamed 2024). Antibiotic‐resistant Salmonella strains threat to public health thus, alternative antibacterial agents need to be investigated. Therefore, this study evaluated concentrations of 25, 50, and 100 g/mL of *Apis mellifera* honey and garlic extracts were tested. As a consequence of the study, researchers showed that honey has the strongest antibacterial activity against Salmonella by performing an inhibition zone between 13.67 and 26.33 mm in all concentrations. However, garlic extracts showed normal moderate antibacterial activity with an inhibition zone between 12.00 and 15.67 mm. As a result of this study, researchers provided potential natural antibacterial agents against the threatening Salmonella.

Another *Candida albicans* study demonstrated that clover honey has anti‐biofilm activity against Candida. This in vitro study aimed to evaluate the activity of clover honey in degrading the biofilm formation of *Candida albicans* in vitro. The results demonstrated that clover honey exerted inhibitory activity against *Candida albicans* at a MIC50 value of 31.60% w/v. Additionally, observations of SEM images and clover honey altered the morphology of *Candida albicans* and decreased the thickness of the forms of biofilm. The findings of this study concluded that clover honey has an antifungal role on *Candida albicans* by degrading mature biofilm form (Masfufatun et al. 2024).

According to recent studies, different honey samples possess antimicrobial roles on different microorganism strains. Therefore, numerous studies and researchers suggested that natural antimicrobial and therapeutic mixture honey samples promising future in this field. In addition, researchers have argued that honey can take place in the health sector as a therapeutic component in bacterial and fungal infections by supporting this feature with further in vivo and human studies.

### 4.2 Anticancer

Approximately, 320 distinct types of honey are present in the world consisting of different sources of plants. The physical and chemical composition of each type of honey differs based on plants and flowers (Meo et al. 2017). Recently, different types of natural honey have been investigated in the context of cancer treatments and cancer‐related situations. Cancer types such as breast, liver, colorectal, and the rest of many cancers have been studied both in experiments and clinical trials (Eisa et al. 2024; Ode Sumarlin et al. 2023; Sánchez‐Martín et al. 2023; Eteraf‐Oskouei and Najafi 2021). Various mechanisms in the alteration of cancer mechanisms have been observed so far. Recent studies have been evaluated, and some remarkable studies were discussed and explained in detail.

In several studies, honey and its constituents were combined with different drugs or treatments to investigate the anticancer effects and mechanisms on cancer cell lines (Qanash et al. 2023; Hamadou et al. 2023; Zhang et al. 2023). A study evaluating the anticancer effects of sitagliptin (Sita, an oral medication primarily used in the management of diabetes Type 2) and bee honey extract on colon cancer (Eisa et al. 2024). In this study, Caco‐2 cells were used in the comparison of impacts to 5‐fluorouracil (5‐Fu) and other distinct combinations. After the study, results revealed that honey extract, alone and in combination with traditional drugs, efficiently hindered the proliferation of colon cancer by interfering with the Raf‐1 pathway as well as promoting programmed cell death (PCD) by activating p53 and Caspase 3 proteins. The production of apoptotic cytokines, IL‐6 and IL‐8 were also promoted and the levels of pro‐inflammatory (IL‐1α and IL‐1β), and anti‐inflammatory, IL‐4 and IL‐10, were adjusted in a way that allowed the body to fight against colon cancer more efficiently. As a result, honey extract possesses the potential to be a complementary therapy for colon cancer by both promoting apoptosis and modulating inflammatory responses. In another study conducted with various bee products such as honey, bee venom, royal jelly, pollen, perga, and propolis, the impact on harmful E*scherichia coli* biofilms and HCT116 colon cancer cell lines was investigated (Akkoyunlu and Dülger 2024). In cancer studies, honey (50% conc.) was found to be effective against HCT116 colon cancer cell lines by hindering cell proliferation at a rate of 86.51% at the end of 48 h. On the other hand, royal jelly and bee venom also showed strong levels of cytotoxic effects. Ultimately, all bee products increased the expression of caspase 9 protein, and all results highlight the potential effects aiming at proliferative diseases through caspase activation.

Besides colon cancer, lung cancer, and its complications are one of the most common cancer types worldwide (Tao 2019). In attributed studies for lung cancers, a study by Dalet et al. examined honey samples from the giant honey bees ( *Apis dorsata* ) and whether the anticancer effects of cyclophosphamide (CP) in human lung carcinoma (A549) cells (Dalet et al. 2023). The investigation focused on the effects of a low concentration of honey on gene expression resulting in an increased CP cytotoxicity, along with an upregulation of the CYP450 genes CYP2J2 and CYP1B1. An increase in the expression of the pro‐apoptotic gene, CASP8, and a decrease in the expression of the anti‐apoptotic gene BLC2 were also observed. The ingredients of *A. dorsata* bee honey, namely phytosphingosine, and sphinganine, were identified as effectors in the attributed mechanism and showed significant anticancer effects. In another study conducted by using lactic acid bacteria (LAB) from Egyptian immature citrus honey, two LAB isolates, *Lactobacillus acidophilus* Ch2 and *Levilactobacillus brevis* Ch1, were used specifically and targeted their secondary metabolites for antibacterial and anticancer activities (Elsayed et al. 2024). The results demonstrated that both bacterial strains possessed significant cytotoxic effects on the human non‐small lung cancer cell line (A549). Ultimate results showed reductions in cell viability to 39.5% for Ch1 and 18.76% for Ch2. A detailed high‐performance liquid chromatography‐quadrupole time‐of‐flight mass spectrometry (HPLC‐QTOF) analysis also concluded 27 metabolites exerting antibacterial and anticancer properties, and their potential in the food sector as medicinal alternatives or biocontrol agents were indicated. Another study, focusing on three different cancer types (including lung A549 and cervical HeLa) and anticancer properties of different sources namely Manuka honey and Saudi honey along with *Peganum harmala* was conducted (Alhawiti et al. 2024). According to whole results, Manuka honey possessed a significant inhibitory effect on cervical cancer cells (HeLa), while Saudi honey implied a significant inhibition of lung cancer cells (A549). It is also indicated that Saudi honey may be used in the management of lung cancers. It is also recalled that these natural products could be possibly used as alternative or complementary sources combined with conventional cancer therapies and favoring fewer side effects.

In other aspects, breast cancer (BC) is one of the most common types of cancer in the world currently and a major cause of death for women (Márquez‐Garbán et al. 2024). Because of the high prevalence of breast cancer, the anticancer effects of honey have been studied extensively until today (Al‐Eisa et al. 2023; Ghramh et al. 2023). Significant results were obtained in a study in which Manuka honey was used to examine antitumor effects, particularly against breast cancer cells (MCF‐7) (Márquez‐Garbán et al. 2024). Manuka honey hindered the proliferation of MCF‐7 cells compared to conventional tamoxifen (antiestrogen agent) in vitro. Also, Manuka honey specifically inhibited the growth of MCF‐7 cells, whereas did not affect the growth of non‐malignant human mammary epithelial cells (HMECs). Other positive results are the promotion of apoptosis in MCF‐7 cells via the activation of PARP, AMPK phosphorylation, and the inhibition of the AKT/mTOR signaling pathway. In vivo studies, Manuka honey was able to hinder tumor growth in MCF‐7 breast cancer models by up to 84%. To sum up, all results indicate that Manuka honey is a promising natural anticancer agent. Again, one recent study has investigated the antibacterial and anticancer effects of four Palestinian honey samples (Abu‐Farich et al. 2024). The results indicated that the anticancer effects of cornflower (PH2) and milk thistle (PH3) against MDA breast cancer cells showed significant cytostatic activity and a decrease in cell viability up to 43%. And also, the cytostatic effects were associated with phenolic compounds of honey such as caffeic acid, rutin, and salicylic acid. Moreover, honey samples led to a significant inhibition of cell migration, up to 85%. All results ultimately suggested that the phenolic compounds of honey possess a potential for anticancer treatments in terms of cytostatic and antimigration properties of honey. Another study focusing on the anticancer properties of Yemeni Sidr honey (YSH) on various cancer lines concluded that the use of YSH on breast cancer cell lines (MDA‐MB‐231 and MCF‐7) and cervical cancer cell lines (HeLa) almost inhibited cell proliferation and promoted apoptosis in cancer cells (Almnayan and Lafrenie 2024). In the study phase, treatment with 1% (w/v) YSH was applied for 48–72 h to cells. Furthermore, the non‐malignant HBL‐100 cell line proved to resist YSH, concluding that YSH possessed selectivity for cancer cell types. Although further research is essential, YSH may be thought to be a promising agent in anticancer therapies. The anticancer studies of honey samples are diverse, and ongoing studies are present worldwide. In a recently published study, two types of honey were investigated on MCF‐7 breast cancer cells: Ziziphus jujube honey (JH) and commercial honey (CH) (Karbasi et al. 2024). The cytotoxic assays were performed by MTT, and the results indicated that JH was a more efficient option in the inhibition of MCF‐7 cell growth than CH according to IC50 values, 170 and 385.3 μg/L, respectively. Also, in a scratch assay, it was indicated that JH could be more efficient in reducing cancer cell migration dose‐dependently. In the context of pro‐apoptotic gene (Bax, p53i, and p21) upregulation and anti‐apoptotic gene (Bcl‐2) downregulation, significant positive effects were also determined. All these affirmative results concluded that honey containing higher phenolic compounds, exerting higher antioxidant properties, and showing higher diastatic activity could be exploited in the suppression of tumor progression via reduced cell proliferation, metastasis inhibition, and apoptosis induction. In another remarkable study in 2024, fir honeydew honey was evaluated with several parameters, including the anti‐migration effects on MCF‐7 breast cancer cell lines and fibroblasts (Dżugan et al. 2024). The scratch assay results concluded that fir honeydew honey remarkably hindered the migration of MCF‐7 cancer cells without applying minimal impact on normal fibroblast movement, indicating the potential medicinal use of fir honeydew honey in anticancer treatments.

Clinical studies provide novel and further aspects in every type of discipline, but meta‐analyses are also a good way to evaluate the current knowledge and findings (Elfar et al. 2023). In the anticancer properties of honey samples, certain studies are present. In a recent meta‐analysis, head and neck cancer patients were investigated for the effect of honey samples in the management of radiotherapy‐induced severe oral mucositis, often in randomized controlled trials (Li et al. 2024). An extensive literature scanning across major databases such as PubMed, Web of Science (WOS), and the Cochrane Library, was conducted up to December 2023. After all specific eliminations, out of 176 records, 10 studies (599 radiotherapy patients) were selected. The ultimate results indicated that different honey samples were found effective in reducing the prevalence of severe mucositis, relieving pains efficiently, and contributing to reducing weight loss in patients due to radiotherapy. In severe mucositis, it was also indicated that the type of honey was not important for reduction effects, meaning most types of honey possessed similar impacts. As a result, it may be concluded that radiotherapy‐induced oral mucositis may be managed with honey, especially for patients requiring long‐term applications. In another study, Idriss et al. studied raw and powdered Manuka honey (MH) for its antiproliferative effects on various human murine cancer cell lines and performed an extensive metabolomics analysis (Márquez‐Garbán et al. 2024). Both MH types caused an inhibition of tumor cell growth dose‐dependently in viability cells. It was also indicated that throughout the cell lines, distinct responses were recorded. Human cancer cell lines, such as MDA‐MB‐231 (breast cancer) and A549 (lung cancer), showed relative resistance to treatment according to two independent assays. All findings resulted in the potential effects of MH as a natural source of anticancer agents.

Honey is a remarkable source of anticancer treatments through bioactive components, including phenolic compounds and flavonoids. This bioactive source of nutrition triggers the induction of apoptosis, inhibition of angiogenesis, the regulation of oxidative stress, and the modulation of immune response. As mentioned, honey is an effective source in various cancer types such as breast, colon, liver, and prostate cancers, as well as improves the efficacy of traditional therapies and reduces side effects. In vitro and in vivo studies also emphasize the significance of honey in selective toxicity to cancer cells and its tumor‐suppressing capabilities. Further investigations and clinical trials are necessary to neutralize the use and therapeutic dosages of distinct honey samples.

### 4.3 Anti‐Inflammatory

Inflammation is the innate response mechanism exerted naturally by the immune system of an organism to harmful pathogens in case of infections and in which a variety of cellular and humoral immunity is developed (Ingawale et al. 2015). Inflammation and oxidative stress together, are also correlated with inflammatory responses and influence multiple signaling pathways synergistically (Ranneh et al. 2017; Reuter et al. 2010). Reactive oxygen species, the direct result of oxidative stress emerging in mitochondria, trigger a set of transcription factors such as ERK, JNK, MAPK, NF‐κB, and P38 participating in the production of pro‐inflammatory cytokines and mediator factors. Consequently, the interaction between ROS and pro‐inflammatory leads to metabolic alterations in which the commencement of inflammatory effects results in pathophysiological disorders (Ranneh, Akim, et al. 2021; Duman and Karav 2025). In recent years, studies regarding the inhibitory effects of honey on chronic and acute inflammation, as well as associated gene expression (Erejuwa et al. 2014). The potential effects of honey in the regulation of inflammatory responses is a trending interest and some remarkable studies have been conducted recently (Kwon et al. 2023; Ren et al. 2023; Shahid et al. 2023; Holubová et al. 2023; Tel‐Çayan et al. 2023; Emin Duru et al. 2023). Some of these studies and consecutive results have been discussed to assess their importance and the affiliated mechanisms behind these effects.

A study regarding the anti‐inflammatory and anti‐endotoxemic effects of monofloral honey derived from *Hovenia dulcis* Thunb. (HMH) investigated the mitigation of inflammation (Hasitha Maduranga Karunarathne et al. 2024). In the inflammatory context, HMH significantly decreased the production of pro‐inflammatory mediators, including nitric oxide and prostaglandin E2, as well as CK such as tumor necrosis factor‐ α and interleukin‐12 in lipopolysaccharide (LPS)‐stimulated RAW 264.7 macrophages. All these downregulation effects were attributed to the inhibition of the NF‐ κB signaling pathway. The study was conducted on a zebrafish larvae model, in which LPS microinjection was performed to trigger inflammation. The mechanism effect of HMH is the elimination of mortality and minimization of inflammatory malfunctions, as well as the inhibition of pro‐inflammatory gene expressions. The study also demonstrated the capability of HMH to activate the Nrf2‐HO‐1 pathway in which nuclear translocation of Nrf2 is increased and expression of HO‐1 is decreased. Due to the HO‐1 inhibitor, the induced anti‐inflammatory responses of HMH were reversed, explaining the critical role of the Nrf‐2‐HO‐1 pathway in these effects. In terms of anti‐inflammatory and anti‐endotoxemic potential effects of HMH, this study is attributed as a pioneer and could be exploited as a therapeutic agent in inflammation. In another highlighted study, focusing on Centauri Honey produced in pristine alpine regions of Türkiye, Filibe et al. investigated the physicochemical properties, and biological activities of Centauri Honey as well as its nutritional composition (Santos Filipe et al. 2024). As for the anti‐inhibitory effect, this high‐quality honey demonstrated remarkable nitric oxide inhibition in vitro which is a sign of its reduction in inflammation capacity and immune response‐enhancing effects. This assay was performed using RAW 264.7 macrophage cells, with an IC50 = 17.86 ± 1.45. All findings indicate the health benefits and potential anti‐inflammatory effects of Centauri Honey, but further research is recommended. Mostly, the health effects of honey stem from its phenolic constituents, because of flower and plant origin. In a study in which the anti‐inflammatory effects of flavonoids from Manuka honey and also methyl syringate, another key phenolic compound in this honey, were investigated and the results indicated the prominent affirmative effects of Manuka honey (Main et al. 2024). During clinical trials, primary human neutrophils were exposed to phenolic compounds both alone and combined, and results for ROS activity reductions and neutrophil extracellular trap formations were assessed. According to the results, Manuka honey was capable of decreasing NET formation by up to 91% and ROS activity by up to 36%. As for methyl syringate, both NET levels and ROS activity decreased by 68% and 66%, respectively. Complementary results demonstrated that the anti‐inflammatory effects of Manuka honey are promising and methyl syringate is a major component in the mitigation of pro‐inflammatory neutrophil responses. From a different perspective, oligosaccharides (Mel) isolated from Alhagi honey were investigated for their therapeutic effects on liver fibrosis symptoms (Lv et al. 2024). Alhagi pseudoalhagi‐derived alhagi honey is known for having anti‐tumor, anti‐oxidative, anti‐inflammatory, anti‐bacterial and hepatoprotective properties. Thus, the isolated compound, Mel, successfully mitigated carbon tetrachloride (CCl4)‐induced liver injury. Mel also contributed to improved antioxidative enzyme (glutathione peroxidase, superoxide mutase) activity, reduced oxidative stress markers, and hindered inflammatory factors (TNF‐α, IL‐1β, IL‐6). Consequently, it was suggested that Mel may be a promising treatment agent for liver fibrosis and its side effects such as inflammation and oxidation.

Various types of analytical and chemical assays are present in the assessment of several properties of honey and its constituents. In certain cases, combinations of honey with effector agents influence the efficacy of these properties. A study, aimed at the investigation of therapeutic effects of *Mentha pulegium* essential oil (MPEO) and thyme honey, assessed the antioxidant, anti‐inflammatory, and dermatoprotective properties of these compounds (Assaggaf et al. 2024). Antioxidant properties were demonstrated via DPPH●, hydrogen peroxide, and xanthine oxidase assays. MPEO demonstrated a remarkable antioxidant activity with IC50 values of 21.13, 29.53, and 16.49 μg/mL in DPPH●, H_2_O_2_, and XO assays, respectively. Thyme honey, on the other hand, exhibited IC50 values ranging from 14.67 to 29.53 μg/mL. The TH‐MPEO mixture, however, enhanced antioxidant effects, achieving together IC50 values of 15.45, 14.67, and 22.06 μg/mL, though slightly less effective than standard controls. In vitro and in vivo anti‐inflammatory assays demonstrated significant anti‐inflammatory and dermoprotective effects, again combined with thyme honey. One of the most common inflammations is edema, described as the swelling caused by too much fluid trapped in the body tissues. In an attributed study by Miranda et al., honey from *Melipona beecheii* bees, used for therapeutic effects dating back to Hispanic times, was assessed for its wound‐healing and anti‐inflammatory properties (Ramírez Miranda et al. 2024). In this study using albino mice, it was demonstrated that the honey sample achieved wound‐healing contributions, both granulation tissue formation and contraction of wound edges. In the context of anti‐inflammatory activity, the honey successfully led to reductions in edema, compared to the control group. In wound treatment, it was suggested that *M. beecheii* honey was a milestone for specifically topical applications.

To sum up, honey is a strongly efficient natural product in inflammatory conditions as well as other affirmative health effects. Besides triggering direct mechanisms, honey also contributes to organism defense factors to fight against inflammation indirectly. More diverse and novel approaches to clinical trials and honey types are necessary for further investigations and analysis.

### 4.4 Antioxidant

Free radicals caused by oxygen are natural intermediate molecules in the organism. However, these radicals damage cellular compartments and severely break down the structure of DNA. Antioxidants strive for these free radicals to eliminate their harmful effects on the metabolism (Jaganathan and Mandal 2009; Yaribeygi et al. 2024). Honey, with its health‐promoting properties, possesses natural antioxidants carrying no side effects potentially harmful to health. The ingredients found in honey, such as phenolic compounds, catalase, peroxides, and flavonoids play an important role in its antioxidative properties. The abundance level of these organic compounds is a determinant of the antioxidative levels of honey species (Khalil et al. 2010). Numerous studies have been designed and conducted to assess the antioxidant potential of honey species (Magoshi et al. 2023; Wahyono et al. 2024; Bazaid et al. 2023; Mduda et al. 2023; Saroğlu et al. 2023).

In a multifaceted study, the physicochemical properties of semi‐wild Jara honey, which is from Western Georgia, were explored (Abashidze et al. 2024). The complete analyses concluded that phenolic compounds of Jara honey were correlated with the antioxidant activity, which was performed by DPPH assay. DPPH assay revealed that the Jara honey exhibited significant antioxidant activity, with 50% less mass to inhibit DPPH radical. Owing to total phenolic compounds, the unique chemical composition and potential health benefits were indicated after this characterization study of Jara honey. In a more common sense, commercial Manuka honey (MH) and its antioxidant properties were investigated due to the well‐known methylglyoxal (MGO) content and health benefits of MH (Kaźmierczak‐Barańska and Karwowski 2024). Once again, the results of DPPH demonstrated the phenolic content of MH and correlated radical scavenging activity, yet the equivalents of MH present higher antioxidative effects. Different floral honeys are also a suitable option for altered properties and efficiency. In this concept, three rare unifloral kinds of honey, namely ailanthus, fennel, and raspberry, were investigated in the context of physiochemical properties, antioxidant capacity, as well as antimicrobial activity, and phenolic content (Saftić Martinović et al. 2024). Among all kinds of honey, ailanthus honey exhibited the highest levels of phenolic compounds, and a significant DPPH antioxidant activity was also demonstrated. All results concluded the potential of these honey samples and applications for appropriate application fields.

Comprehensive and detailed analyses and studies are valuable sources for the evaluation of current data and future potential for research (Jiang et al. 2023; Kumari et al. 2023; Wu, Zhao, et al. 2023; Keskin et al. 2023; Khattabi et al. 2023; Ziaei et al. 2024). In a comprehensive study investigating honey samples from more than 90 regions in the Middle East, in Iran and Azerbaijan areas, between 2020 and 2021, the antioxidant activities were evaluated (Shakoori et al. 2024). The results of the study concluded that antioxidant levels in honey were primarily altered by the plant species, as the highest impact was observed in Rosaceae, Amaranthaceae, Fabaceae, and Asteraceae families. This study also correlates the altitude level with increasing antioxidant activity, ultimately concluding that the key factors for antioxidant property levels are directly associated with plant origin, geographical factors, and altitude. In a study conducted in Australia on native stingless bee honey, 36 distinct samples were evaluated for the analysis of their physicochemical, sensory, and antioxidant properties via the Ferric Reducing Antioxidant Power (FRAP) assay (Mello dos Santos et al. 2024). Along with considerable composition and sensory differences, phenolic content, and antioxidant activity, assays demonstrated the potential of honey samples for antioxidant activity and suggested that these kinds of honey could be a natural antioxidant source. Generally, studies including honey types from one country or region are a preferred way to analyze inclusive pools. In a dedicated study, nine monofloral Greek honey types, namely fir, chestnut, citrus, erica, cotton, Jerusalem thorn, pine, oak, and thyme were investigated for antioxidant properties and related phenolic contents over the Manuka honey. After FRAP assays, oak, and chestnut kinds of honey exhibited the highest phenolic content (203.75 mg GAE/100 g) and following antioxidant activity (106.2 mg AAE/10 g). The significant finding of this study revealed that there is a strong correlation between antioxidant activity and electrical conductivity. It was also indicated that fir, pine, and erica honey possessed relatively higher antioxidant activity than model Manuka's honey, resulting in a high potential capacity for Greek monofloral honey. In Iran, a complementary study performed with 10 Iranian and two commercial multi‐floral honey types investigated the antioxidant and melissopalynological (the study of pollen contained in honey) properties (Hajian‐Tilaki et al. 2024). Focusing on the phenolic profile and color characteristics, results demonstrated that local honey samples originating from Fabaceae and Asteraceae were assessed by antioxidant assays (DPPH, FRAP, and β‐carotene bleaching assay). According to outcomes, multi‐floral honey samples exhibited higher antioxidant activity compared to commercial honey samples. A remarkable correlation was also determined between honey color, melanoidin content, and antioxidant activity, where phenolic compounds namely syringic acid and luteolin are dominant. It was also noted that the phenolic content profile was not a direct factor in antioxidant capacity, since sugar feeding and post‐harvest processes could influence the outcomes. In a comprehensive study completed with honey collected from five monofloral legume species, a complete set of versatile physicochemical and phytochemical properties were investigated (Sultana et al. 2024). Among all results, antioxidant activity assays revealed that there was a variety between species in the context of antioxidant activity. Purple clover honey exhibited the highest level of antioxidant effects in the FRAP assay (7.3 mmol Fe^2+^/kg). Moreover, High‐Performance Thin‐Layer Chromatography (HPTLC) analysis and DPPH assay combined to determine the various bioactive compounds in honey samples. The presence of these compounds was found to contribute to the antioxidant activity of honey. This comprehensive study was a leading data for monofloral honey species for future applications.

The general concept in the investigation of honey samples generally comprises the characteristics and physicochemical properties (Alarjani and Mohammed 2024; Zapata‐Vahos et al. 2023; Tahirovića et al. 2023; Romero‐Márquez et al. 2023; Fratianni, Amato, et al. 2023; Lawag et al. 2023). In Algeria, various regional honeys were investigated by this means along with antioxidant activity via DPPH and FRAP assays (Harbane et al. 2024). From 53 botanical families, a total of 129 pollen species were identified, in which honeys from Ceratonia siliqua, Eucalyptus, and honeydew exhibited relatively higher antioxidant activities correlated with phenolic and flavonoid contents. This local study underscores the significant correlations between botanical origin and physicochemical properties, concluding the result that better characterization and investigation are necessary. In some cases, enrichments of honey aid in the enhancement of honey properties. Rapeseed honey enriched with propolis, bee bread, and bee pollen were comprehensively analyzed (Derewiaka et al. 2024). Properties such as color analysis, total polyphenol content, and antioxidant activity were examined in this study. In the context of antioxidant properties, the DPPH assay revealed that tested bee products demonstrated strong antioxidant activity, with which rapeseed honey enriched with 5% bee pollen exhibited the highest levels of antioxidant activity and ingredients. In the same concept, eight monofloral honey samples from central and eastern Morocco were analyzed for phenolic composition, antioxidant activity, and anti‐glycation properties (Lakhmili et al. 2024). After all examinations, the highest phenolic and flavonoid ingredients were measured as 163.83 ± 1.84 mg GAE/kg and 84.44 ± 1.20 mg CE/kg, respectively. Among all honey types, zantaz honey exerted a strong level of antioxidant activity via ABTS, DPPH, β‐carotene bleaching, FRAP, and ORAC assays. The ORAC value was measured at 4.65 mM Trolox equivalent per gram. The floral origin and polyphenolic profiles were also linked with HPLC‐DAD analyses.

A different animal study was performed with 40 male albino Swiss mice to detect the antioxidative role of honey on Parkinson's disease (Ayobami 2022). A dopaminergic neurotoxicant, which is 1‐methyl‐4phenyl‐1,2,3,6‐tetrahydropyridine (MPTP) can stimulate parkinsonism. In this study, another molecule that is a precursor of dopamine, L‐3,4‐dihydroxyphenylalanine (L‐DOPA) can transported into the brain, thus researchers investigated the combination of L‐DOPA and honey effect on parkinsonism induced with MPTP. As a result of the study, researchers provided that honey protected the longevity of L‐DOPA and improved neuroprotection of the dopaminergic neurons of the substantia nigra region of the brain. Additionally, after MPTP‐induced parkinsonism, honey samples reduced radical species and improved the gait abnormality of Swiss mice. Therefore, researchers suggested that honey is a potentially active substance for the treatment of Parkinson's disease and should be counted in the diets of Parkinson's patients or elderly people. A complex study is aimed to demonstrate the antioxidant and cytotoxicity effect of 12 different honeys (Mohammed et al. 2024). For this study, to define antioxidant activity researchers performed a free radical scavenging DPPH assay, and as a result of this assay, most active honeys were found in Assasia nilotica and Acacia seyal honeys. The cytotoxicity of researched 12 kinds of honey was tested and for all honey samples the cytotoxicity activity was not reported, all are safe.

Antioxidative effects are a general property for honey samples since the essential components behind this property are polyphenolic compounds, mainly flavonoids, and stem from floral sources (Bolat et al. 2024; Sejbuk et al. 2024). Since bees collect nectar from flowers to produce honey, their primary energy source, the ingredients of honey include all major components for all requirements of bees from nutritional to protective aspects. Polyphenols are plant‐based compounds responsible for plant defense and primarily possess antioxidative properties. Considering the diversity of plant species, honey from different floral sources should be investigated to extend the knowledge and be exploited for their potential health effects.

### 4.5 Prebiotic Effect

Honey is considered a strong therapeutic agent by evidence and has been used in the enhancement of digestive health (Abou El‐Soud 2012; Kuropatnicki et al. 2018). The bioactivity of honey is directly associated with the floral source and certain types of honey are especially classified as bioactive (Irish et al. 2011; Carter et al. 2016). In the context of prebiotic effects, honey occasionally possesses oligosaccharides that cannot be digested by the human gastrointestinal (GI) tract, leading to studies investigating the prebiotic capability of honey. In vitro, animal and human studies have been established and concluded that certain types of honey may exert prebiotic activity to promote GI health (Schell et al. 2022; Fratianni, De Giulio, et al. 2023). Honey supports and induces the development of probiotics namely Bifidobacterium and Lactobacillus species, such as *B. longum* , *B. bifidum* , and *B. infantis* ; *Lactobacillus acidophilus* , *Lactobacillus plantarum* , and *Lactobacillus rhamnosus* and so on. The promotional effects of honey on these species are attributed to oligosaccharide prebiotics, and certain prebiotics proved the prebiotic effects on these strains (Shin and Ustunol 2005; Schell et al. 2022; Haddadin et al. 2007). Additional studies also suggest that honey possesses affirmative effects on the bacterial strains from the GI tract as well as promotes the development of probiotics (Mohan et al. 2017). Oligosaccharide composition is a determinant in the variable prebiotic activity, and honey from different floral origins provides altered prebiotic effects within this framework (Kolayli et al. 2012). Different honey samples from variable floral origins have been investigated in the determination of differential effects and their potential effects on the GI tract bacteria. Recent studies have established and designed differential frameworks discussed in this chapter.

In general concept, the design of the experiment relies on three basic procedures: the choice of honey sample, probiotic organism, and feeding. A study investigating the effects of pine honey on the viability of probiotic strains, *Lactobacillus acidophilus* LA‐5 and *Bifidobacterium animalis* subsp. lactis BB‐12, and the properties of probiotic yogurts (Ayaz et al. 2024). A complete set of three groups were designed but for honey samples, there were three subgroups: A control without honey, 5% pine honey, and 7% pine honey. After 3 weeks of storage and gradual analyses, results indicated that pine honey did not exhibit remarkable effects on *L. acidophilus* LA‐5 strains, but somewhat, 7% pine honey was successful in enhancing the viability of *B. animalis* subsp. lactis BB‐12. To sum up, the study resulted that 5% and 7% pine honey could be used as a prebiotic source in yogurt manufacture. In some cases, antimicrobial and prebiotic properties are investigated simultaneously. In a similar manner, a recent study investigated the antimicrobial and prebiotic potentials of six distinct honey types, namely basil, mint, oregano, rosemary, savory, and thyme (Nazzaro et al. 2024). Besides its antimicrobial effects, these honey samples were evaluated for the prebiotic potential on *Lacticaseibacillus* casei Shirota, *Lactobacillus gasseri* , Lacticaseibacillus paracasei subsp. paracasei, and Lacticaseibacillus rhamnosus species. The complete effects of honey samples include the growth of probiotics, adhesive capacity, antioxidant activity, and cytotoxic properties. Moreover, supernatants from *L. casei* Shirota, *L. gasseri* , and *L. paracasei* successfully prohibited biofilms of the pathogens, indirectly supporting antimicrobial activity. All results concluded that certain honeys hold a capacity to possess antimicrobial and prebiotic potentials. In the context of honey studies, several honey compartments are included to extensively investigate the effects of bee products. In a study guided by this concept, the potential effects of honeybee brood biopeptides (HBb‐Bps) and Maillard reaction conjugates of these biopeptides with honey, glucose, and fructose as functional food ingredients were investigated (Ounjaijean et al. 2024). With these alterations, HBb‐Bps exhibited a promotion in the growth of probiotic species (*Lactiplantibacillus plantarum* and *Lactococcus lactis* ), as well as the stimulation in short‐chain fatty acid production (SCFA). Additionally, antioxidant activity and ACE inhibition were also observed, indicating that HBb‐Bps is a promising candidate for health‐promoting functional foods, yet further investigations on in vivo organisms are recommended.

Novel methodologies in the exploitation of food products are a trending and frequently applied phenomenon in recent studies (Duman et al. 2021; Nogueira‐Rio et al. 2024). An affiliated study regarding the microencapsulation of honeydew honey and royal jelly into rye bran heteropolysaccharide‐based biopolymeric microparticles via spray‐drying was aimed at the creation of controlled‐release systems and in the preservation of antioxidant, immunomodulatory, and prebiotic properties (Kowalska et al. 2024). In this comprehensive study, versatile and several results were obtained. Created microcapsules successfully resisted stomach acidity and digestive enzymes. These microcapsules also enhanced the prebiotic effects of Bifidobacterium spp. and lactic acid bacteria by promoting growth, survival, and adhesive abilities. Fermentation of these microcapsules was also investigated and resulted in a significant increase (39.2%) in SCFA production compared to native bee products. This study indicated a potential for nutraceutical applications of microencapsulated bee products. A different research study is performed to show the potential prebiotic effect of oligosaccharides of 2 different types of honey (named KR and KL) from Apis spp, and Trigona spp bees (Susilowati and Azkia 2022). In this in vitro study, the prebiotic activity of these honey oligosaccharides was demonstrated with bacterial growth assay of *Lactobacillus acidophilus* and *Bifidobacterium longum* . According to the procedure of the study, bacteria were inoculated at different duration times like 0, 24, and 48 h and maximum bacterial growth for *Lactobacillus acidophilus* was determined for KR honey at 24 h incubation time for KL honey at 48 h incubation time. As a consequence of the results, oligosaccharides of different honey types can stimulate the growth of gut microbiota. In addition, the longer the incubation period, the more the pH value also decreases due to the accumulation of SCFA and lactic acid, which leads to the death of pathogenic microbes. Overall, honey studies regarding prebiotic effects demonstrate that honey and its derivatives exhibit remarkable prebiotic activity by promoting the growth and viability of probiotics as well as enhancing gut health by increasing SCFA production.

### 4.6 Neuroprotective Effect

The brain is the most sensitive organ to oxidative stress due to its unsaturated fatty acid content, high metabolic activity, low repair capacity, and high iron load (Zulkifli et al. 2023). The development of neurological and neurodegenerative diseases, such as Alzheimer's, Parkinson's, epilepsy, and Amyotrophic lateral sclerosis (ALS), is significantly influenced by oxidative stress (Ashrafi et al. 2007). Excessive free radical production leads to neuronal damage, protein oxidation, lipid peroxidation, and DNA damage. Neuronal death is highly induced, especially by glutamate‐induced neurotoxicity and increased reactive oxygen species (Yaacob et al. 2017). Therefore, honey has been the subject of research in terms of improving brain health with its rich bioactive content and antioxidant and anti‐inflammatory properties.

A research study aimed to assess the impacts of ethyl acetate chestnut honey fraction (EACH) on induced cognitive impairment and neurotoxicity in C57BL/6 mice (Jeong et al. 2024). Researchers injected scopolamine (SCO) into mice cognitive impairment was generated and an investigation of glutamate‐induced neurotoxicity was performed in the HT22 mouse hippocampal neuronal cell line. For purposes of the study, water maze and passive avoidance tests were performed and treatment of EACH demonstrated sufficiently increased recovery of the neuronal cell model with the reduction of accumulation of ROS and neurınal apoptosis. Additionally, due to the antioxidant proteins of honey, nuclear factors, calcium response, and brain‐derived neurotrophic factor expressions improved, EACH can protect the nerve neurons from oxidative damage and dysfunction of cognition. Therefore, researchers suggested that this EACH can be used as a therapeutic agent for the treatment of neuronal issues. Furthermore, investigations were conducted into potential therapeutic solutions for neurodegenerative diseases, including Parkinson's and Alzheimer's (Hasim et al. 2024). According to the aim of the study, Tualang honey was tested on the kainic acid (KA) induced rat model to observe tumor necrosis factor‐alpha and caspase‐3 activity. 72 male rats were classified into six different groups, which were treated with Tualang honey and Cresyl Violet, and Fluoro‐Jade C staining was used for the evaluation of the number of degenerative and viable neurons. As a consequence of the study, up‐regulation of tumor necrosis factors and caspase‐3 activity was observed and led to neuronal loss; however, treatment with tualang honey performed a role as a protective agent against neuronal loss and degenerations by decreasing the expression of tumor necrosis factor and caspase‐3 activity. Owing to the result of the study, Tualang honey possesses a protective role against neuronal damage and improves cognitive health.

In addition to rat model studies, researchers also investigated the potential healing effects of Kelulut honey on Alzheimer's disease (Shaikh et al. 2024). 26 male Srague Dawley rats were used for this study, and divided into 3 different groups as control, induced, and treated groups. For induction of Alzheimer's disease, researchers give 6.25 μg of amyloid‐β1 through intrahippocampal injection. After injection, Kelulut honey was administered to the induced group and they were subjected to behavioral tests. Administration of Kelulut honey demonstrated improved mobility of rats. Outcomes of researchers suggested consumption of Kelulut honey for 28 days possesses a healing impact on Alzheimer's disease by decreasing the pathological burden of the disease. Furthermore, researchers investigated the combination effect of honey and levodopa protective role for the substantia nigra region, which is related to Parkinson's disease, in the brain (Sulaimon et al. 2024a). Oxidative and inflammatory cascade damage impacts neurological health and leads to Parkinson's disease, which is an extensive health issue for about 8.5 million individuals (Eker et al. 2023). In this study, 1‐methyl‐4‐phenyl‐1,2,3,6‐tetrahydropyridine (MPTP) induced Swiss mice were used for the analysis of honey treatment against oxidative stress. Male Swiss mice were classified into a control group of 27 mice and a Parkinson's model group, with control mice given phosphate‐buffered saline, honey, or levodopa supplementation for 21 days. In addition, Parkinson's models were either given honey and levodopa pre‐supplementation or not. Following the administration of the supplements, behavioral studies were performed and the results were recorded after 2 and 8 days. As a result of the behavioral analyses, the researchers observed that motor and movement skills were better in the groups of mice that received honey and levodopa supplements. There was also a sufficient increase in the expression of glutathione (GSH), an antioxidant marker, and nuclear factor 2 (Nrf2). In line with these results, the researchers showed that MPTP‐induced oxidative stress was eliminated and reduced after honey and levodopa supplementation.

Another animal study aimed to show the impacts of Chestnut honey on brain damage of high‐fat diet and obese‐induced mice. Normally, obesity is a factor that triggers and increases infection and oxidative stress. Accordingly, infection and an increase in ROS can cause brain damage, leading to the loss of neurons and affecting the flow of the nervous system. Therefore, the ability of honey and D‐limonene to ameliorate neurodegeneration in obese mice induced by high‐fat diet feeding was investigated (Terzo et al. 2023). Mice fed heavy diets for 10 weeks were divided into 4 groups: control, honey‐supplemented, D‐limonene‐supplemented, and both‐supplemented groups. Neuronal loss, oxidative stress, infection, and Alzheimer's disease‐related gene expression assays were analyzed. Increased neuronal apoptosis, elevation of pro‐inflammatory cytokines, and oxidative stress markers were attenuated in the honey and D‐limonene‐supplemented groups; moreover, the beneficial health effects were more clearly observed when honey and D‐limonene were taken together. Furthermore, in controlling Alzheimer's disease‐related gene expressions, amyloid plaque accumulation was reduced and synaptic function improved. As a consequence of the study, researchers suggested obesity causes a harmful impact on neuropathology and inflammation conditions of Alzheimer's diseases, but consumption of long‐term honey and D‐limonene possess recent neuroprotective therapy agents. In addition to the animal studies, an in vitro study was performed and aimed to increase knowledge about the chemical character and health impacts of Italian leguminous honey, including *Medicago sativa* L., *Astragalus nebrodensis*, *Ceratonia silique L*., *Indigofera tinctoria L*., and *Onobrychis viciifolia* Scop (Fratianni et al. 2024). Polyphenols and vitamin C contents of these honeys were analyzed and in vitro anti‐inflammatory properties and their ability to reactivate acetylcholinesterase, butyrylcholinesterase, and tyrosinase enzymes associated with neurodegenerative diseases. According to the results of the chemical content analysis, the highest polyphenol content was found in *Medicago sativa* , 408 μg/g, while the lowest polyphenol content was found in *Indigofera tinctoria* , 110 μg/g. In the analysis of three different enzymes, *Onobrychis viciifolia* showed the highest antioxidant activity and showed a strong inhibitory effect of 74% on the butyrylcholinesterase enzyme. Plant‐derived bioactive compounds are receiving more attention than synthetic compounds in the treatment of diseases such as neurodegeneration. Researchers have shown that the presence of compounds such as gallic acid, 5‐hydroxymethyl‐furfural, rutin, and taxifolin enhances the neuroprotective effect of honey. These properties suggest the use of honey in combination with “sensitive diets” and traditional therapies as a potential natural supplement. In the future, investigating the effects of honey on enzymes associated with other metabolic diseases will further substantiate its potential as a therapeutic agent.

Studies investigating the neuroprotective effect of honey have recently highlighted ist potential to mitigate neuronal loss, normalize neuronal transmission, and safeguard areas of the brain like the substantia nigra through its antioxidant and anti‐inflammatory action. However, the majority of studies have been limited to animal models and in vitro studies. Adding more broad, especially recent clinical studies on the therapeutic effects of honey tp the literature will provide further evidence of its potential to treat damage to brain.

### 4.7 Anti‐Diabetic

Diabetes is a metabolic disease that is becoming increasingly widespread worldwide (Naqvi et al. 2023). It is caused by a problem that occurs during insulin secretion and use (Anti‐Diabetic and Anti‐Oxidant Activities of Devdarvadyarishta in StreptozotocinInduced Diabetic Rats 2023). This problem causes blood sugar levels to change, and if this condition continues for a long time, it causes problems in protein, carbohydrate, and lipid metabolism (Iftikhar et al. 2023). It is generally known as hyperglycemia, increased hunger, and increased urination, and it generally causes delayed wound healing in diabetic patients (Iftikhar et al. 2023).

Some foods can exhibit functional properties thanks to their bioactive content (Sarıtaş, Duman, et al. 2024; Sarıtaş, Portocarrero, et al. 2024). These foods can positively affect health due to their bioactive components (Coşkun et al. 2024). Honey is also considered one of these foods (Sharma et al. 2024). Honey does not cause rapid increases in blood sugar thanks to its low glycemic index. In addition, it can reduce/prevent oxidative stress thanks to the antioxidants it contains (Anti‐Diabetic and Anti‐Oxidant Activities of Devdarvadyarishta in StreptozotocinInduced Diabetic Rats 2023). On the other hand, the anti‐inflammatory properties of honey can prevent damage to organs such as the kidneys, liver, and pancreas (Mohd Nasir et al. 2024; Iftikhar et al. 2023). In addition, honey is thought to modulate insulin release and protect pancreatic cells from damage (Iftikhar et al. 2023). Finally, honey consumption can reduce the likelihood of heart‐related complications associated with diabetes by elevating high‐density lipoprotein (HDL) levels and lowering low‐density lipoprotein (LDL) levels (Koodathil et al. 2023; Iftikhar et al. 2023).

Zebrafish is an emerging vertebrate model with numerous benetifs, such as its compact size remarkable experimental efficiency. Malaysia has one of the highest diabetes prevalence rates in the Western Pacific, resulting in an annual economic burden of 600 million US dollars. This study aims to investigate the antidiabetic effects of green honey (GH) using a zebrafish model. Adult zebrafish underwent overfeeding and received intraperitoneal injection (IP) of streptozotocin (STZ) on Days 7 and 9. The research aimed to investigate the anti‐diabetic properties of green honey by performing an oral sucrose tolerance test (OSTT). Measurements were recorded at three intervals: 30, 60, and 120 min post‐treatment and sucrose administration. The experimen was conducted using a model with a sample size of five. The study was performed in six groups. These groups are (1) non‐diabetic, no intervention, (2) non‐diabetic, receiving 3 μL GH, (3) diabetic, no treatment, (4) diabetic, receiving 3 μL GH, (5) diabetic, receiving 6 μL GH, (6) diabetic, administered acarbose. Fasting blood glucose levels for non‐diabetic (non‐DM) and diabetic (DM) groups were evaluated before and after the 10 days of diabetic induction. DM groups (excess of food and two injections of STZ) have caused a significant increment in the fasting blood glucose to 11.55 mmol/L (*p* < 0.0001). Both GH treatments effectively decreased postprandial blood glucose levels and the area under the curve in the oral glucose tolerance test (OSTT). Based on these results, investigate the potential of green honey as a natural molecule for treating hyperglycemia and as a promising, natural alternative before conventionel treatment begins. Yet the mechanisms of this benefits require additional investigation, and its potential application in the treatment of diabetes in humans should be evaluated. In a like manner, to investigate the effect of stingless bee honey towards kidney and liver in STZ‐exposed diabetic rats, serum biochemical indices (and histological changes) were evaluated (Mohd Nasir et al. 2024). In the kidney sections of the rats treated for 12 days, it was observed that there was an alleviation of kidney damage compared to the control group. This situation was revealed by the decrease in hyphropic changes and the increase in the number of small capillary bundles called glomeruli, which filter the blood in the kidneys. In the study conducted on periodic acid‐Schiff (PAS) stain carbohydrates in the kidneys, it was concluded that the thickening of the glomerulus basement membrane was prevented/reduced in the honey‐treated group, which obtained similar results to the non‐diabetic control group. When the liver tissues were examined, it was seen that STZ‐induced diabetic liver toxicity and hyperlipidemia of the rats treated with stingless bee honey were improved. When the biochemical results were evaluated, it was demonstrated that there were decreases in glucose levels and levels of enzymes indicating liver damage.

In a recent study investigating the antidiabetic effects of bitter honey, the antidiabetic properties were evaluated in vitro and in vivo (Koodathil et al. 2023). In vitro experiments were conducted to investigate the inhibitory effect of bitter honey at different concentrations on alpha‐amylase and alpha‐glucosidase. The inhibitory effect of bitter honey at different concentrations in the 0.2–10 mg/mL range was evaluated. According to the results, 0.2 mg/mL of bitter honey inhibited the alpha‐amylase enzyme by 38.45%. This value was 42.69% inhibition when the drug acarbose was used as a positive control at the same concentration. According to these results, alpha‐amylase enzyme exhibited a similar effect to acarbose. Bitter honey was tested in the concentration range of 0.1–1 mg/mL in order to evaluate inhibition of alpha‐glucosidase enzyme. 0.1 mg/mL of bitter honey inhibited the alpha‐glucosidase enzyme by 16.46%, while acarbose at the same concentration exhibited 112.86% inhibition. According to the results of the in vivo study, diabetic rats induced with streptozotocin‐nicotinamide and treated with bitter honey showed significant improvement compared to untreated rats. A notable reduction in fasting blood sugar levels was observed in diabetic rats treated with bitter honey compared to their untreated counterparts. Additionally, treatment with bitter honey led to an increase in HDL levels and a decrease in LDL and total cholesterol levels. Furthermore, improvements were observed in liver and kidney functions in diabetic rats following bitter honey treatment. To investigate the dose‐dependent effects of bitter honey on diabetes, two different doses (200 and 400 mg/kg) were administered. While both doses resulted in improvements in metabolic parameters and pancreatic tissue, the reduction in blood sugar levels and the increase in HDL levels were more pronounced in rats treated with 400 mg/kg bitter honey.

Another study conducted by Iftikhar et al. aimed to reveal the potential role of Manuka honey in pancreatic regeneration (Iftikhar et al. 2023). For this purpose, diabetic rats were treated with 3 g/kg Manuka honey once a day for 21 days. When the results of the study were evaluated, it was determined that insulin levels increased and glucose levels decreased in diabetic rats. In addition, there was an increase in the expression levels of transcription factors known as MAFA, PDX‐1, INS‐1, INS‐2, NEUROG3, NKX6‐1, and NEUROD, which play a role in the regeneration of pancreatic beta cells. The findings of the study revealed that Manuka honey modulates blood sugar levels by reducing oxidative stress in diabetic rats and supports the regeneration of pancreatic beta cells.

Overall, studies suggest that honey and its bioactive components may reduce the risk of diabetes and help alleviate its symptoms. Hence, further studies are needed to prove these outcomes.

### 4.8 Wound Healing and Skin Health

Honey is a naturally derived substance abundant in phenolic compounds, enzymes and vitamins, minerals, and sugars, with health benefits such as anticarcinogenic, antioxidant, antimicrobial, anti‐inflammatory, and anti‐carcinogenic properties, and has been popular in biomedical therapy applications in wound care and healing since ancient times (Scepankova et al. 2021). In this field, some data have been presented to the literature with current clinical, animal and in vitro studies (Khattabi et al. 2023; Darwis et al. 2024; Suryadinata et al. 2024). According to these data, the roles of honey in wound healing include skin tissue repair and acceleration of cell proliferation, angiogenesis, and fighting against infections caused by pathogenic microorganisms.

The existence of Pseudomonas aeruginosa on the skin can cause skin infections, burns, and skin wounds. For this major global issue, researchers evaluated the activities of medical grade honey (MGH) for the investigation of alternative treatments. Normally, silver‐based alternatives were used for treatments of burns and wounds; however, these designed things can delay healing time by inducing skin irritation. Thus, a study investigated the antibacterial and healing effects of MGH and silver‐based (Eker, Duman, et al. 2024; Duman, Eker, et al. 2024) wound‐care formulas (Boekema et al. 2024). To perform the aim of the study, researchers analyzed wound healing and bacterial survival parameters, such as keratinocyte proliferation and re‐epithelialization after treatment with Medi honey (Manuka), MGH‐gel, and AgNO3. As a consequence of the analysis, MGH‐gel and AgNO3 decreased the population of Pseudomonas aeruginosa and Medi honey exhibited less activity than MGH‐gel. As a consequence, clinical cases exhibited the property of MGH therapy in infected burns, and after 8 and 13 weeks researchers evaluated the healing levels. After 13 weeks, noticeable important improvements have been reported. Thus, treatment of MGH can be used as an alternative therapy for *Pseudomonas aeruginosa* ‐infected burns and wounds.

A different clinical study demonstrated that Argania Spinosa honey has high levels of polyphenols and due to these constituents, possesses antioxidant and antibacterial activity in the care of surgical wounds (Khattabi et al. 2023).

Researchers tested antibacterial properties on different bacterial populations, including Klebsiella pneumonia, *Staphylococcus epidermidis* , *Staphylococcus aureus* , *Pseudomonas aeruginosa* , *Enterococcus faecalis* , *Escherichia coli* , *and Bacillus subtilis*, and analysis results showed that inhibition zone of honey is average between 7.7öö and 12.07 mm for Staphylococcus aureus, Klebsiella pneumonia, *Bacillus subtilis* . Consequences of case action showed that topical application of Argania Spinosa honey can heal surgical wounds in 18 days. Therefore, researchers suggested that this honey can be used as an alternative wound‐care agent in surgical wound cases. A pilot study was aimed to demonstrate the combination of hydrogel formation of pectin‐honey impacts on surgical site infection, which is an infection in some parts of the body, especially celiotomy in horses (Gandini et al. 2024). Generally, it can be treated with antibiotics; however, antibiotic resistance leads to the investigation of different natural sources like Manuka honey. Therefore, in this study, Manuka honey and pectin combined and produced a hydrogel, which is pectin‐honey hydrogel (PHH). PHH provided inhibited microbial area and moist wound medium in the horses challenged with emergency laparotomy. According to the study, 44 horses were divided into two groups for PHH application therapy and no treatment. No macroscopically noticeable adverse outcomes have been reported in horses treated with PHH without suturing the surgical wound. Therefore, the researchers concluded that the application of PHH during surgery was safe and reduced the prevalence of regional surgical infections in horses. Additionally, researchers demonstrated antibacterial activity against *E. coli* by using a broth culture assay with the phytochemicals of honey, including 3‐phenyllactic acid, p‐coumaric acid, and phloretin. The most efficient antibacterial agent 3‐phenyllactic acid for inhibition of the *E. coli* population. The pursuit of this p‐coumaric acid and a lower concentration of phloretin allowed the bacterial population to grow. Researchers suggested that these outcomes may be useful for the improvement of antibacterial healing subjects for skin infection treatment (Kassym et al. 2024). A similar study showed various types of honey including unprocessed, pasteurized and Manuka can inhibit the growth of *E. coli* , and *Staphylococcus epidermidis* (Lemmen et al. 2024). 20 trials were performed and after that results exhibited that Manuka and unprocessed honey have more crucial impacts than pasteurized ones. Therefore, researchers suggested that topical application of honey on the skin might be helpful for infection treatment.

In fact, chronic wounds represent a major global health concern, cause significant discomfort to patients, and require a long time and the therapeutic component to treat. In this context, dressings with biologically active ingredients are promising. Therefore, a research study is aimed at investigating the application of polycaprolactone (PCL) enriched with manuka honey and essential oils for improved wound healing (Gallo et al. 2024). According to the results of the study, compatibility with human dermal fibroblasts and the expression of genes involved in cell‐tissue regeneration stimulated and inhibited the proliferation of bacterial strains such as *S. aureus* and *P. aeruhinosa*.

A different recent study aimed to comparison of Sumatera forest honey and Triamcinolone acetonide, which is a medicine used to relieve mouth sores, for application in the healing of traumatic ulcers in Wistar rats (Darwis et al. 2024). Traumatic ulcer causes lesions on oral mucosa and they lead to inconvenience in chewing, speaking, and swallowing. In this study, researchers divided 32 rats into two different groups including a treatment group with 100% forest honey and Triamcinolone acetonide as a control group. It is observed that the impacts of honey on the 1, 5, 7, 10, and 14 days of treatment and outcomes showed that honey significantly reduced the size of ulcers and erythema. Researchers suggested that Sumatera forest honey can be used effectively for the healing of wounds on oral mucosa. Another similar in vivo study demonstrated wound healing impacts and anti‐inflammatory activity of honey produced from Mexico, *Melipona beecheii* Bees on male albino mice. For observation of the study, researchers used pirfenidone and indomethacin as positive controls against honey application. The consequence of the experiments, after 1‐day treatment, formation of granulation tissue and contraction of the wound edges sustainability has been supported. Thus, researchers suggested Mexican honey can enhance wound healing, and also honey treatment exhibited that can reduce edema at a higher level than 1 mg/mL indomethacin application. Therefore, this type of honey can be used as an alternative for topical treatment of wounds (Ramírez Miranda et al. 2024). Another study used a nano‐functionalized Medical grade honey for infected wound therapy in guinea pigs. For this study, researchers developed Medical grade honey based on Ulmo honey ( *Eucryphia cordifolia* ), and the copper (Cu) element is known as a developing agent for the treatment of wounds, thus, investigation of the healing effect of developed medical‐grade honey with copper nanoparticles (Duman, Akdaşçi, et al. 2024; Eker, Akdaşçi, et al. 2024) (CuNPs) combination on infected and non‐infected wounds therapy. According to analysis including bacteriological, histopathological, and content of collagen fibers, researchers observed that CuNPs and medical grade honey can be used as antibacterial agents, and due to their regulatory role for inflammatory processes collagen and fibers maturation is supported (Salvo et al. 2023). Moreover, for prevention and control of skin infections interest in natural elements is increasing. A study incorporating both in vivo and in vitro experiments formulated an antibacterial combination of honey and *Lactobacillus plantarum* , which was assessed for its effectiveness wound‐healing therapy. The developed formulation was composed of 10% honey and 1 × 109 CFU/mL of *Lactobacillus plantarum* and the antimicrobial impact of this formulation was tested with an in vitro study by biofilm crystalline violet staining and fluorescent staining techniques. Additionally, with 8 weeks of old Sprague–Dawley rats, the wound‐healing impact of the developed formulation was concluded. Consequences of staining experiments developed formulations that exhibited reduced biofilm formation of *Staphylococcus aureus* and *Pseudomonas aeruginosa* . Thus, it is considered that the viability of bacteria in infected skin wounds can be reduced with the formulation. Decreasing the number of bacteria in the infected wounds leads to the enhancement of connective tissue to improve skin healing. Therefore, researchers suggested that this formulation accelerates the healing of skin wounds (Li et al. 2023).

A recently performed case report demonstrated the managing impact of medical‐grade honey and foam on diabetic foot ulcers with osteomyelitis. The broad spectrum of diabetes mellitus comprises the formation of ulceration and infection, thus diabetic foot ulcer is one of the most common in patients. For this study, a 55‐year‐old man with hypertension and diabetes mellitus, had a foot ulcer and medical‐grade honey was used for wound healing therapy. Due to the antimicrobial impact of medical‐grade honey, it can inactivate distinct bacteria, thus it can be used for the treatment of wounds. After 6 weeks of treatment, the wound size reduced significantly and demonstrated improving progress (Dzani and Nair 2024). A similar study was performed on patients challenged with diabetic foot ulcers who were treated with honey and aloe vera extracts (Sutrisno 2024). According to the study result, wound improvement was observed and indicated with 82.4%. Another study was performed to show the antifungal impact of honey on dermatophytes, which causes skin, nail, and hair infection in the body, isolated from children and farmers in North East Nigeria (Ifeoma et al. 2024). According to the study, Epidermophyton floccosum, Microsporum canis, and Trichophyton species types were determined from isolates taken from skin, nails, and hair. With the MIC and agar well diffusion methods, the inhibitory impact of honey on Epidermophyton floccosum, Microsporum canis, and Trichophyton species and inhibition zone were calculated. Investigating the antifungal impacts of honey, it was observed that 100% honey concentration was effective against different fungal species. Epidermophyton floccosum showed an inhibition zone of 25 mm, Microsporum canis 40 mm, and Trichophyton species 36 mm in diameter. In MIC tests with 60% honey dilution, Epidermophyton floccosum and Trichophyton species showed very little growth, but Microsporum canis showed no growth. This was also found to be the case for 5‐ and 7‐day cultures. With these outcomes, researchers suggested honey can be a potential therapy for fungal infections. Furthermore, it investigated the feasibility of using a nanofibrous scaffold containing aloe vera and honey extract that mimics skin tissue and thus can be applied for the treatment of diabetic wounds (Dhiman et al. 2024). The modeled skin structure was made effective against diabetic wound problems such as high blood sugar, inflammation, and oxidative stress. It was observed that this scaffold model demonstrated remarkable mechanical properties, biodegradability, antibacterial, and anti‐diabetic effects, promoted skin cell proliferation, and exhibited promising wound healing resutls in in vivo experiments. In conclusion, a skin model loaded with plant extracts is a potential model for an effective self‐renewal and wound‐healing process. Another pilot randomized‐controlled trial (RCT) study evaluated the impact of honey and povidone‐iodine combination on healing of acute laceration, which is a common skin problem in Indonesia (Suryadinata et al. 2024). Researchers compared with paraffin gauze and povidone‐iodine combination for healing duration of acute laceration by single‐blind, and RCT methods through three divided groups as honey (*n*: 12), povidone‐iodine (*n* = 11), and paraffin (*n* = 12). In 3 different groups, wound healing was realized in a timely manner, with an average of 10 days in the honey group, 5–15 days in the paraffin group, and 4–13 days in the povidone‐iodine group. In conclusion, the researchers showed that honey clinically accelerates and improves healing and is a low‐cost therapeutic agent compared to povidone‐iodine.

As a consequence of these studies, honey and other combinations with components can impact wound healing time in clinical and animal studies. However, the conditions of these studies are insufficient for further human studies and investigations of therapeutic agents. Therefore, the usage of honey on skin and wound healing should be studied in detail, especially if different honey types may impact skin health in different ways.

## 5 Conclusion and Future Perspective

As a result, honey possesses many health benefits with both physical and chemical characteristics. Honey, which has been used as both medical medicine and food in many fields from the past to the present, is nowadays of great interest to researchers and the therapeutic effects of different honey varieties are being tried to be explained. The health benefits of honey are largely due to its content of phenolic compounds, flavonoids, organic acids, enzymes, and various micronutrients. These bioactive compounds reduce oxidative stress through their free radical scavenging effects, modulate inflammatory processes by regulating cytokine levels, and suppress the growth of pathogens while supporting the balance of beneficial microbiota thanks to their antimicrobial properties. In addition, honey's mechanisms for strengthening the immune system, accelerating wound healing, and positively affecting metabolic balance (e.g., glucose and lipid metabolism) are increasingly being proven.

Honey is unartificial treatment that is remarkable for its healing properties. With the contribution of phenolic compounds, proteins, enzymes and oligosaccharides, it is effective against bacterial and fungal infections and provides benefits in various types of cancer through mechanisms such as apoptosis induction, inhibition of angiogenesis and regulation of immune response. In addition, thanks to its anti‐inflammatory and antioxidant properties, it reduces neuronal loss, regulates nerve conduction protects brain regions and shows neuroprotective effects. In addition to these, honey supports intestinal health with its prebiotic effect, nourishes intestinal motility and beneficial bacteria population while destroying the pathogen population. Well known for its benefits to skin health, honey has been shown to speed up wound healing and promote skin regeneration. The current literature presents numerous in vitro and in vivo studies supporting the biological activities of honey; however, clinical studies remain limited, and variables such as dosage, type (monofloral vs. polyfloral), and bioavailability have not been sufficiently elucidated. Furthermore, the effects of honey on the gut microbiota have not yet been fully elucidated at the signaling pathway level. Furthermore, the effects of honey on the gut microbiota have not yet been fully elucidated at the signaling pathway level. Additionally, more research is needed on whether honey is suitable for pharmaceutical companies and human clinical applications. Nevertheless, comprehensive clinical research is required to gain deeper insights into the therapeutic potential of various honey types. Additionally, in vitro and limited animal model studies in the literature are insufficient to explain the biological pathway of the potential therapeutic effects of honey and to show under which conditions the bioactive component can show the appropriate effect.

Moreover, new human clinical trials investigating how the therapeutic effects shown in these studies are affected under conditions in human metabolism, their therapeutic utility and the development of therapeutic drugs should be included in the literature. To continue, the inclusion of studies evaluating the therapeutic effects that may occur as a result of the combination or interaction of bioactive components with each other in the literature will support a comprehensive understanding of the health benefits of honey. It is also very important to investigate the changes in the therapeutic effects of varying component concentrations of different honey types on therapeutic effects shown and to present data to the literature about which honey varieties can show which therapeutic properties more predominantly. The inclusion of such clinical trials in the literatüre will provide a more detailed understanding of honey's therapeutic drugs.

**FIGURE 5:**
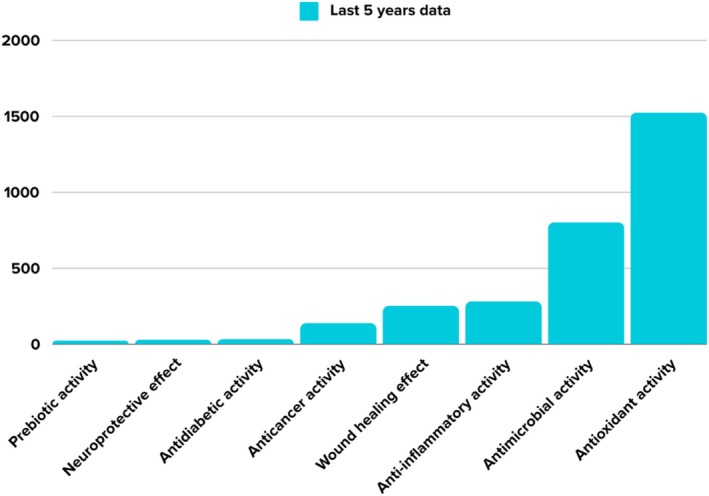
According to data from the Web of Science platform, the graphical figure of honey therapeutic impacts researches entitled “which (antimicrobial, wound healing, etc.) health impact” for last 5 years within 15,920 total research documents (Clarivate 2024).

Furthermore, although the therapeutic effect of honey studies in the literature recently attracted the interest of researchers, research, determining honey health effects is limited and it is crucial to bring new research, which are investigating the therapeutic health impacts of honey, to the literature (Figure 5). Especially, the introduction of new studies about prebiotic, neuroprotective, anti‐diabetic, anticancer, wound healing, anti‐inflammatory, antimicrobial and antioxidant effects of honey need to be investigated for further developing treatment agents.

In this context, new clinical trials and human applications will support the potential of honey as a natural medicine and provide new data sources for pharmaceutical companies. Thus, honey will gain more place in the field of health and pave the way for innovative natural therapies.

## Author Contributions

Sercan Karav structured the overall content of the paper. Ecem Bolat managed the general editing and coordination of the authors. The remaining authors were involved in editing and organizing the manuscript. All authors contributed to the article and approved the final version submitted. All authors have read and consented to the published version of the manuscript.

## Funding

The authors have nothing to report.

## Ethics Statement

The authors have nothing to report.

## Consent

The authors have nothing to report.

## Conflicts of Interest

The authors declare no conflicts of interest.

## Data Availability

The authors have nothing to report.

## References

[fsn371645-bib-0001] Aati, H. , S. Y. Aati , H. S. Bahr , et al. 2025. “Tamarix Honey Phenolics Attenuate Cisplatin‐Induced Kidney Toxicity by Inhibition of Inflammation Mediated IL‐6/STAT3/TNF‐α and Oxidative Stress‐Dependent Nrf2/Caspase‐3 Apoptotic Signaling Pathways.” Frontiers in Pharmacology 16: 1584832. 10.3389/fphar.2025.1584832. 40689208 PMC12271747

[fsn371645-bib-0002] Abashidze, N. , I. Djafaridze , M. Vanidze , et al. 2024. “Physicochemical Characterization and Antioxidant Activity of Jara Honey Produced in Western Georgia.” Applied Sciences 14: 6874. 10.3390/app14166874.

[fsn371645-bib-0003] Abdulreda, G. R. , Z. A. Waheed , N. H. Sarhan , and N. N. Abbas . 2023. “Antibacterial Activity of Honey on Acne Vulgaris Caused by *Staphylococcus epidermidis* .” pp. 040012.

[fsn371645-bib-0004] Abdulrhman, M. , M. El‐Hefnawy , R. Hussein , and A. A. El‐Goud . 2011. “The Glycemic and Peak Incremental Indices of Honey, Sucrose and Glucose in Patients With Type 1 Diabetes Mellitus: Effects on C‐Peptide Level—A Pilot Study.” Acta Diabetologica 48: 89–94. 10.1007/s00592-009-0167-7. 19941014

[fsn371645-bib-0005] Abel, S. D. A. , and S. K. Baird . 2018. “Honey Is Cytotoxic Towards Prostate Cancer Cells but Interacts With the MTT Reagent: Considerations for the Choice of Cell Viability Assay.” Food Chemistry 241: 70–78. 10.1016/j.foodchem.2017.08.083. 28958561

[fsn371645-bib-0011] Abou El‐Soud, N. H. 2012. “Neveen Helmy Abou El‐Soud Honey Between Traditional Uses and Recent Medicine.” Macedonian Journal of Medical Sciences 5: 205–214.

[fsn371645-bib-0006] Abu‐Farich, B. , H. Hamarshi , M. Masalha , et al. 2024. “Polyphenol Contents, Antibacterial and Antioxidant Effects of Four Palestinian Honey Samples, and Their Anticancer Effects on Human Breast Cancer Cells.” Clinical Traditional Medicine and Pharmacology 18: 1372–1385. 10.22207/JPAM.18.2.60.

[fsn371645-bib-0007] Aburayyan, W. S. , N. Seder , O. Al‐fawares , A. Fararjeh , I. S. Majali , and Y. Al‐Hajaya . 2024. “Characterization of Antibiofilm and Antimicrobial Effects of Trigona Stingless Bee Honey Compared to Stinging Bee Centaurea Hyalolepis and Citrus Honeys.” Journal of Evidence‐Based Integrative Medicine 29: 2515690X241271978. 10.1177/2515690X241271978. PMC11311187 39118572

[fsn371645-bib-0008] Adesoji, F. , and A. Oluwakemi . 2010. “Differential Effect of Honey on Selected Variables in Alloxan‐Induced and Fructose‐ Induced Diabetic Rats.” African Journal of Biomedical Research 11: 191–196. 10.4314/ajbr.v11i2.50706.

[fsn371645-bib-0009] Adeyemo Emmanuel Ayobami . 2020. “Yusuf Robiah Arafah Combination of Honey and L‐Dopa Protected the Dopaminergic Neurons Against MPTP Induced Parkinsonism in Adult Male Swiss Mice.” International Journal of Innovative Science and Research Technology 7: 1313–1318.

[fsn371645-bib-0010] Adeyomoye, O. I. , O. T. Olaniyan , N. Adewumi , and M. M. Anyakudo . 2022. “Honey Supplemented With Vitamin C Prevents Dyslipidaemia and Oxidative Stress Induced by Exposure to Lead Acetate in Wistar Rats.” Indian Journal of Physiology and Pharmacology 65: 229. 10.25259/IJPP_445_2021.

[fsn371645-bib-0012] Afrin, S. , T. Forbes‐Hernandez , M. Gasparrini , et al. 2017. “Strawberry‐Tree Honey Induces Growth Inhibition of Human Colon Cancer Cells and Increases ROS Generation: A Comparison With Manuka Honey.” International Journal of Molecular Sciences 18: 613. 10.3390/ijms18030613. 28287469 PMC5372629

[fsn371645-bib-0013] Afrin, S. , F. Giampieri , T. Y. Forbes‐Hernández , et al. 2018. “Manuka Honey Synergistically Enhances the Chemopreventive Effect of 5‐Fluorouracil on Human Colon Cancer Cells by Inducing Oxidative Stress and Apoptosis, Altering Metabolic Phenotypes and Suppressing Metastasis Ability.” Free Radical Biology & Medicine 126: 41–54. 10.1016/j.freeradbiomed.2018.07.014. 30056083

[fsn371645-bib-0014] Afrox, R. , and E. M. Tanvir . 2016. “Molecular Pharmacology of Honey.” Clinical and Experimental Pharmacology 6: 3. 10.4172/2161-1459.1000212.

[fsn371645-bib-0015] Afshari, J. , S. Davoodi , and S. Samarghandian . 2011. “Honey Induces Apoptosis in Renal Cell Carcinoma.” Pharmacognosy Magazine 7: 46. 10.4103/0973-1296.75901. 21472079 PMC3065157

[fsn371645-bib-0016] Ahmed, E. M. 2015. “Hydrogel: Preparation, Characterization, and Applications: A Review.” Journal of Advanced Research 6: 105–121. 10.1016/j.jare.2013.07.006. 25750745 PMC4348459

[fsn371645-bib-0017] Ahmed, S. , and N. H. Othman . 2013. “Honey as a Potential Natural Anticancer Agent: A Review of Its Mechanisms.” Evidence‐Based Complementary and Alternative Medicine 2013: 1–7. 10.1155/2013/829070. PMC3865795 24363771

[fsn371645-bib-0018] Ahmed, S. , S. A. Sulaiman , A. A. Baig , et al. 2018. “Honey as a Potential Natural Antioxidant Medicine: An Insight Into Its Molecular Mechanisms of Action.” Oxidative Medicine and Cellular Longevity 2018: 8367846. 10.1155/2018/8367846. 29492183 PMC5822819

[fsn371645-bib-0019] Ajibola, A. , J. P. Chamunorwa , and K. H. Erlwanger . 2012. “Nutraceutical Values of Natural Honey and Its Contribution to Human Health and Wealth.” Nutrition & Metabolism (London) 9: 61. 10.1186/1743-7075-9-61. PMC3583289 22716101

[fsn371645-bib-0020] Akanda, M. R. , and B.‐Y. Park . 2017. “Involvement of MAPK/NF‐ΚB Signal Transduction Pathways: Camellia Japonica Mitigates Inflammation and Gastric Ulcer.” Biomedicine & Pharmacotherapy 95: 1139–1146. 10.1016/j.biopha.2017.09.031. 28926923

[fsn371645-bib-0021] Akkoyunlu, A. , and G. Dülger . 2024. “Exploring the Antibiofilm Effects on *Escherichia coli* Biofilm Associated With Colon Cancer and Anticancer Activities on HCT116 Cell Line of Bee Products.” Biofouling 40: 235–244. 10.1080/08927014.2024.2338106. 38584359

[fsn371645-bib-0022] Alaerjani, W. M. A. , S. Abu‐Melha , R. M. H. Alshareef , et al. 2022. “Biochemical Reactions and Their Biological Contributions in Honey.” Molecules 27: 4719. 10.3390/molecules27154719. 35897895 PMC9331712

[fsn371645-bib-0023] ALaerjani, W. M. A. , S. A. Abu‐Melha , K. A. Khan , et al. 2021. “Presence of Short and Cyclic Peptides in Acacia and Ziziphus Honeys May Potentiate Their Medicinal Values.” Open Chemistry 19: 1162–1173. 10.1515/chem-2021-0106.

[fsn371645-bib-0024] Alain Prudence, I. , M. Pierre Celestin , M. Hiberte , I. Josue , and S. Eric . 2024. “The Effect of Honey on Bacteria Isolated From Urinary Tract Infections Among Patients Attending Ruhengeri Referral Hospital.” Journal of Drug Delivery and Therapeutics 14: 10–13. 10.22270/jddt.v14i4.6104.

[fsn371645-bib-0025] Alarjani, W. M. A. , and M. E. A. Mohammed . 2024. “Antioxidant Activities of Saudi Honey Samples Related to Their Content of Short Peptides.” Scientific Reports 14: 24318. 10.1038/s41598-024-74824-4. 39414854 PMC11484816

[fsn371645-bib-0026] Al‐Awadhi, M. A. , and R. R. Deshmukh . 2021. “A Review on Automatic Classification of Honey Botanical Origins Using Machine Learning.” Proceedings of the 2021 International Conference of Modern Trends in Information and Communication Technology Industry (MTICTI); IEEE 4: 1–5.

[fsn371645-bib-0027] Albaridi, N. A. 2019. “Antibacterial Potency of Honey.” International Journal of Microbiology 2019: 1–10. 10.1155/2019/2464507. PMC6589292 31281362

[fsn371645-bib-0028] Alberti, K. G. M. M. , R. H. Eckel , S. M. Grundy , et al. 2009. “Harmonizing the Metabolic Syndrome.” Circulation 120: 1640–1645. 10.1161/CIRCULATIONAHA.109.192644. 19805654

[fsn371645-bib-0029] Al‐Eisa, R. A. , A. A. Ashour , M. Helal , et al. 2023. “Anticancer Effects of Honey Varieties on Human Cells by Studying Some Physical Parameters, Hydrogen Peroxide Content, Catalase, Glucose Oxidase, and Microbial Activities.” Journal of Biobased Materials and Bioenergy 17: 160–166. 10.1166/jbmb.2023.2272.

[fsn371645-bib-0030] Al‐Hatamleh, M. A. I. , M. M. Hatmal , K. Sattar , et al. 2020. “Antiviral and Immunomodulatory Effects of Phytochemicals From Honey Against COVID‐19: Potential Mechanisms of Action and Future Directions.” Molecules 25: 5017. 10.3390/molecules25215017. PMC7672575 33138197

[fsn371645-bib-0031] Alhawiti, N. M. , E.‐L. Myles , A. M. Alhewaitey , Y. M. Almutairi , and F. M. Alhawiti . 2024. “Cytotoxicity, Toxicity and Anticancer Activity of Manuka Honey, Saudi's Honey and *Peganum harmala* Plant Against Cancer Cells.” Journal of Biosciences and Medicines 12: 311–325. 10.4236/jbm.2024.122025.

[fsn371645-bib-0032] Al‐Kafaween, M. A. , M. Alwahsh , A. B. Mohd Hilmi , and D. H. Abulebdah . 2023. “Physicochemical Characteristics and Bioactive Compounds of Different Types of Honey and Their Biological and Therapeutic Properties: A Comprehensive Review.” Antibiotics 12: 337. 10.3390/antibiotics12020337. 36830249 PMC9952753

[fsn371645-bib-0033] All About Honey . 2024. “Everything Your Wanted to Know About Honey.” https://localhoneyfinder.org/AllAboutHoney.php.

[fsn371645-bib-0034] Almasaudi, S. 2021. “The Antibacterial Activities of Honey.” Saudi Journal of Biological Sciences 28: 2188–2196. 10.1016/j.sjbs.2020.10.017. 33911935 PMC8071826

[fsn371645-bib-0035] Almnayan, D. , and R. M. Lafrenie . 2024. “Yemeni Sidr Honey Inhibits Cell Proliferation and Promotes Apoptosis in Human Cancer and Mouse‐Derived Cell Lines.” Targets 2: 64–79. 10.3390/targets2020004.

[fsn371645-bib-0036] Altin‐Celik, P. , M. Derya‐Andeden , H. Eciroglu‐Sarban , and H. Donmez‐Altuntas . 2025. “Antiproliferative and Apoptotic Effects of Pervari Honey on SH‐SY5Y Neuroblastoma Cells.” Medical Oncology 42: 394. 10.1007/s12032-025-02963-3. 40736760

[fsn371645-bib-0037] Alvarez‐Suarez, J. , M. Gasparrini , T. Forbes‐Hernández , L. Mazzoni , and F. Giampieri . 2014. “The Composition and Biological Activity of Honey: A Focus on Manuka Honey.” Food 3: 420–432. 10.3390/foods3030420. PMC5302252 28234328

[fsn371645-bib-0038] Alvarez‐Suarez, J. , F. Giampieri , and M. Battino . 2013. “Honey as a Source of Dietary Antioxidants: Structures, Bioavailability and Evidence of Protective Effects Against Human Chronic Diseases.” Current Medicinal Chemistry 20: 621–638. 10.2174/092986713804999358. 23298140

[fsn371645-bib-0039] Alvarez‐Suarez, J. M. , F. Giampieri , M. Cordero , et al. 2016. “Activation of AMPK/Nrf2 Signalling by Manuka Honey Protects Human Dermal Fibroblasts Against Oxidative Damage by Improving Antioxidant Response and Mitochondrial Function Promoting Wound Healing.” Journal of Functional Foods 25: 38–49. 10.1016/j.jff.2016.05.008.

[fsn371645-bib-0040] 2023. “Anti‐Diabetic and Anti‐Oxidant Activities of Devdarvadyarishta in StreptozotocinInduced Diabetic Rats.” Indian Journal of Traditional Knowledge 22: 68–75. 10.56042/ijtk.v22i1.33710.

[fsn371645-bib-0041] Arung, E. T. , R. Ramadhan , B. Khairunnisa , et al. 2021. “Cytotoxicity Effect of Honey, Bee Pollen, and Propolis From Seven Stingless Bees in Some Cancer Cell Lines.” Saudi Journal of Biological Sciences 28: 7182–7189. 10.1016/j.sjbs.2021.08.017. 34867021 PMC8626249

[fsn371645-bib-0042] Asari, M. A. , M. H. Zulkaflee , K. N. S. Sirajudeen , N. A. Mohd Yusof , and N. S. Mohd Sairazi . 2019. “Tualang Honey and DHA‐Rich Fish Oil Reduce the Production of Pro‐Inflammatory Cytokines in the Rat Brain Following Exposure to Chronic Stress.” Journal of Taibah University Medical Sciences 14: 317–323. 10.1016/j.jtumed.2019.06.004. 31488962 PMC6717079

[fsn371645-bib-0043] Ashrafi, M. R. , S. Shams , M. Nouri , et al. 2007. “A Probable Causative Factor for an Old Problem: Selenium and Glutathione Peroxidase Appear to Play Important Roles in Epilepsy Pathogenesis.” Epilepsia 48: 1750–1755. 10.1111/j.1528-1167.2007.01143.x. 17555528

[fsn371645-bib-0044] Assaggaf, H. , N. El Hachlafi , A. Elbouzidi , et al. 2024. “Unlocking the Combined Action of * Mentha pulegium L*. Essential Oil and Thym Honey: In Vitro Pharmacological Activities, Molecular Docking, and In Vivo Anti‐Inflammatory Effect.” Heliyon 10: e31922. 10.1016/j.heliyon.2024.e31922. 38947443 PMC11214453

[fsn371645-bib-0045] Aumeeruddy, M. Z. , Z. Aumeeruddy‐Elalfi , H. Neetoo , et al. 2019. “Pharmacological Activities, Chemical Profile, and Physicochemical Properties of Raw and Commercial Honey.” Biocatalysis and Agricultural Biotechnology 18: 101005. 10.1016/j.bcab.2019.01.043.

[fsn371645-bib-0046] Ayaz, R. , S. Andiç , and Ş. Oğuz . 2024. “The Effect of Pine Honey on the Viability of Probiotics and Some Properties of Probiotic Yogurt.” Emirates Journal of Food and Agriculture 75: 8099–8112. 10.12681/jhvms.37054.

[fsn371645-bib-0047] Ayobami, A. E. 2022. “Yusuf Robiah Arafah Combination of Honey and L‐Dopa Protected the Dopaminergic Neurons Against MPTP Induced Parkinsonism in Adult Male Swiss Mice.” International Journal of Innovative Science and Research Technology 7: 1313–1318.

[fsn371645-bib-0048] Azman, K. F. , R. Zakaria , Z. Othman , and C. B. Abdul Aziz . 2018. “Neuroprotective Effects of Tualang Honey Against Oxidative Stress and Memory Decline in Young and Aged Rats Exposed to Noise Stress.” Journal of Taibah University for Science 12: 273–284. 10.1080/16583655.2018.1465275.

[fsn371645-bib-0049] Ball, D. W. 2007. “The Chemical Composition of Honey.” Journal of Chemical Education 84: 1643. 10.1021/ed084p1643.

[fsn371645-bib-0050] Baloš, M. M. Ž. , N. S. Popov , J. Z. P. Radulović , I. M. Stojanov , and S. M. Jakšić . 2020. “Sugar Profile of Different Floral Origin Honeys From Serbia.” Journal of Apicultural Research 59: 398–405. 10.1080/00218839.2020.1714193.

[fsn371645-bib-0051] Bardy, J. , A. Molassiotis , W. D. Ryder , et al. 2012. “A Double‐Blind, Placebo‐Controlled, Randomised Trial of Active Manuka Honey and Standard Oral Care for Radiation‐Induced Oral Mucositis.” British Journal of Oral & Maxillofacial Surgery 50: 221–226. 10.1016/j.bjoms.2011.03.005. 21636188

[fsn371645-bib-0052] Barreiros, J. , A. Cepeda , C. Franco , C. Nebot , and B. Vázquez . 2024. “Analysis of Minerals in Honey and Their Nutritional Implications.” Journal of Food Composition and Analysis 136: 106733. 10.1016/j.jfca.2024.106733.

[fsn371645-bib-0053] Bashir, I. , M. H. Rasool , M. Shafique , K. Jabeen , and M. U. Qamar . 2024. “Exploring the Antimicrobial Efficacy of Manuka Honey Against Multidrug‐Resistant and Extensively Drug‐Resistant *Salmonella typhi* Causing Septicemia in Pakistan.” Future Microbiology 19: 1377–1387. 10.1080/17460913.2024.2384260. 39109942 PMC11485888

[fsn371645-bib-0054] Bazaid, A. S. , A. Alsolami , M. Patel , et al. 2023. “Antibiofilm, Antimicrobial, Anti‐Quorum Sensing, and Antioxidant Activities of Saudi Sidr Honey: In Vitro and Molecular Docking Studies.” Pharmaceutics 15: 2177. 10.3390/pharmaceutics15092177. 37765148 PMC10534861

[fsn371645-bib-0055] Becerril‐Sánchez, A. L. , B. Quintero‐Salazar , O. Dublán‐García , and H. B. Escalona‐Buendía . 2021. “Phenolic Compounds in Honey and Their Relationship With Antioxidant Activity, Botanical Origin, and Color.” Antioxidants 10: 1700. 10.3390/antiox10111700. 34829570 PMC8614671

[fsn371645-bib-0056] Beck, I. M. E. , W. Vanden Berghe , L. Vermeulen , K. R. Yamamoto , G. Haegeman , and K. De Bosscher . 2009. “Crosstalk in Inflammation: The Interplay of Glucocorticoid Receptor‐Based Mechanisms and Kinases and Phosphatases.” Endocrine Reviews 30: 830–882. 10.1210/er.2009-0013. 19890091 PMC2818158

[fsn371645-bib-0057] Beltekin, B. 2022. “Nurullah Demir Production and Some Bioactive Properties of Royal Jelly–A Review.” BinBee–Arı ve Doğal Ürünler Dergisi 2: 38–46.

[fsn371645-bib-0058] Beteri, B. , M. Barone , S. Turroni , et al. 2024. “Impact of Combined Prebiotic Galacto‐Oligosaccharides and *Bifidobacterium breve* ‐Derived Postbiotic on Gut Microbiota and HbA1c in Prediabetic Adults: A Double‐Blind, Randomized, Placebo‐Controlled Study.” Nutrients 16: 2205. 10.3390/nu16142205. 39064648 PMC11280236

[fsn371645-bib-0059] Bezerra, A. , H. Fonseca , F. Rodrigues , C. Delerue‐Matos , I. Gouvinhas , and J. Garcia . 2023. “Honey Therapy in Diabetic Foot Ulcers: A Promising Strategy for Effective Wound Healing.” Applied Sciences 13: 12820. 10.3390/app132312820.

[fsn371645-bib-0060] Birru, R. L. , K. Bein , N. Bondarchuk , et al. 2021. “Antimicrobial and Anti‐Inflammatory Activity of Apple Polyphenol Phloretin on Respiratory Pathogens Associated With Chronic Obstructive Pulmonary Disease.” Frontiers in Cellular and Infection Microbiology 11: 652944. 10.3389/fcimb.2021.652944. 34881190 PMC8645934

[fsn371645-bib-0061] Boekema, B. K. H. L. , D. Chrysostomou , G. Ciprandi , et al. 2024. “Comparing the Antibacterial and Healing Properties of Medical‐Grade Honey and Silver‐Based Wound Care Products in Burns.” Burns 50: 597–610. 10.1016/j.burns.2023.10.009. 37940425

[fsn371645-bib-0062] Bolat, E. , S. Sarıtaş , H. Duman , et al. 2024. “Polyphenols: Secondary Metabolites With a Biological Impression.” Nutrients 16: 2550. 10.3390/nu16152550. 39125431 PMC11314462

[fsn371645-bib-0063] Bonnici, L. , S. Suleiman , P. Schembri‐Wismayer , and A. Cassar . 2023. “Targeting Signalling Pathways in Chronic Wound Healing.” International Journal of Molecular Sciences 25: 50. 10.3390/ijms25010050. 38203220 PMC10779022

[fsn371645-bib-0064] Boo, Y. C. 2019. “P‐Coumaric Acid as an Active Ingredient in Cosmetics: A Review Focusing on Its Antimelanogenic Effects.” Antioxidants 8: 275. 10.3390/antiox8080275. 31382682 PMC6720745

[fsn371645-bib-0065] Bose, D. , A. C. Famurewa , A. Akash , and E. M. Othman . 2024. “The Therapeutic Mechanisms of Honey in Mitigating Toxicity From Anticancer Chemotherapy Toxicity: A Review.” Journal of Xenobiotics 14: 1109–1129. 10.3390/jox14030063. 39189178 PMC11348124

[fsn371645-bib-0066] Browne, E. , S. Kavanagh , and S. Devery . 2025. “The In Vitro Antioxidant and Immunomodulatory Effects of the Irish Monofloral Ivy and Heather Honey Varieties.” International Journal of Molecular Sciences 26: 3625. 10.3390/ijms26083625. 40332151 PMC12027192

[fsn371645-bib-0069] Brudzynski, K. 2006. “Effect of Hydrogen Peroxide on Antibacterial Activities of Canadian Honeys.” Canadian Journal of Microbiology 52: 1228–1237. 10.1139/w06-086. 17473892

[fsn371645-bib-0067] Brudzynski, K. , K. Abubaker , M. Laurent , and A. Castle . 2011. “Re‐Examining the Role of Hydrogen Peroxide in Bacteriostatic and Bactericidal Activities of Honey.” Frontiers in Microbiology 2: 213. 10.3389/fmicb.2011.00213. 22046173 PMC3201021

[fsn371645-bib-0068] Brudzynski, K. , K. Abubaker , and T. Wang . 2012. “Powerful Bacterial Killing by Buckwheat Honeys Is Concentration‐Dependent, Involves Complete DNA Degradation and Requires Hydrogen Peroxide.” Frontiers in Microbiology 3: 242. 10.3389/fmicb.2012.00242. 22783246 PMC3389331

[fsn371645-bib-0070] Bunyatratchata, A. , A. Parc , J. M. L. N. de Moura Bell , et al. 2023. “Release of Bifidogenic N‐Glycans From Native Bovine Colostrum Proteins by an Endo‐β‐N‐Acetylglucosaminidase.” Enzyme and Microbial Technology 162: 110138. 10.1016/j.enzmictec.2022.110138. 36252443

[fsn371645-bib-0071] Can, Z. , O. Yildiz , H. Sahin , E. Akyuz Turumtay , S. Silici , and S. Kolayli . 2015. “An Investigation of Turkish Honeys: Their Physico‐Chemical Properties, Antioxidant Capacities and Phenolic Profiles.” Food Chemistry 180: 133–141. 10.1016/j.foodchem.2015.02.024. 25766810

[fsn371645-bib-0072] Cani, P. D. , R. Bibiloni , C. Knauf , et al. 2008. “Changes in Gut Microbiota Control Metabolic Endotoxemia‐Induced Inflammation in High‐Fat Diet–Induced Obesity and Diabetes in Mice.” Diabetes 57: 1470–1481. 10.2337/db07-1403. 18305141

[fsn371645-bib-0073] Cárdenas‐Escudero, J. , C. Mármol‐Rojas , S. Escribano Pintor , D. Galán‐Madruga , and J. O. Cáceres . 2023. “Honey Polyphenols: Regulators of Human Microbiota and Health.” Food & Function 14: 602–620. 10.1039/D2FO02715A. 36541681

[fsn371645-bib-0074] Cardona, F. , C. Andrés‐Lacueva , S. Tulipani , F. J. Tinahones , and M. I. Queipo‐Ortuño . 2013. “Benefits of Polyphenols on Gut Microbiota and Implications in Human Health.” Journal of Nutritional Biochemistry 24: 1415–1422. 10.1016/j.jnutbio.2013.05.001. 23849454

[fsn371645-bib-0075] Carter, D. A. , S. E. Blair , N. N. Cokcetin , et al. 2016. “Therapeutic Manuka Honey: No Longer So Alternative.” Frontiers in Microbiology 7: 569. 10.3389/fmicb.2016.00569. 27148246 PMC4837971

[fsn371645-bib-0076] Caturano, A. , M. D'Angelo , A. Mormone , et al. 2023. “Oxidative Stress in Type 2 Diabetes: Impacts From Pathogenesis to Lifestyle Modifications.” Current Issues in Molecular Biology 45: 6651–6666. 10.3390/cimb45080420. 37623239 PMC10453126

[fsn371645-bib-0077] Chari, R. V. J. 2008. “Targeted Cancer Therapy: Conferring Specificity to Cytotoxic Drugs.” Accounts of Chemical Research 41: 98–107. 10.1021/ar700108g. 17705444

[fsn371645-bib-0078] Chaudhary, P. , P. Janmeda , A. O. Docea , et al. 2023. “Oxidative Stress, Free Radicals and Antioxidants: Potential Crosstalk in the Pathophysiology of Human Diseases.” Frontiers in Chemistry 11: 1158198. 10.3389/fchem.2023.1158198. 37234200 PMC10206224

[fsn371645-bib-0079] Chen, S. , F. Wu , C. Yang , et al. 2022. “Alternative to Sugar, Honey Does Not Provoke Insulin Resistance in Rats Based on Lipid Profiles, Inflammation, and IRS/PI3K/AKT Signaling Pathways Modulation.” Journal of Agricultural and Food Chemistry 70: 10194–10208. 10.1021/acs.jafc.2c03639. 35971648

[fsn371645-bib-0080] Chepulis, L. M. , and E. Francis . 2012. “An Initial Investigation Into the Anti‐Inflammatory Activity and Antioxidant Capacity of Alpha‐Cyclodextrin‐Complexed Manuka Honey.” Journal of Complementary & Integrative Medicine 9: 1–12. 10.1515/1553-3840.1646. 23023642

[fsn371645-bib-0081] Chin, S. W. , A. Azman , and J. W. Tan . 2024. “Incorporation of Natural and Synthetic Polymers Into Honey Hydrogel for Wound Healing: A Review.” Health Science Reports 7: e2251. 10.1002/hsr2.2251. 39015423 PMC11250418

[fsn371645-bib-0082] Chua, L. S. , J. Y. Lee , and G. F. Chan . 2013. “Honey Protein Extraction and Determination by Mass Spectrometry.” Analytical and Bioanalytical Chemistry 405: 3063–3074. 10.1007/s00216-012-6630-2. 23292042

[fsn371645-bib-0083] Cianciosi, D. , T. Y. Forbes‐Hernández , S. Afrin , et al. 2018. “Phenolic Compounds in Honey and Their Associated Health Benefits: A Review.” Molecules 23: 2322. 10.3390/molecules23092322. 30208664 PMC6225430

[fsn371645-bib-0084] Clarivate . 2024. “Web of Science Core Collection.”

[fsn371645-bib-0085] Combarros‐Fuertes, P. , J. M. Fresno , M. M. Estevinho , M. Sousa‐Pimenta , M. E. Tornadijo , and L. M. Estevinho . 2020. “Honey: Another Alternative in the Fight Against Antibiotic‐Resistant Bacteria?” Antibiotics 9: 774. 10.3390/antibiotics9110774. 33158063 PMC7694208

[fsn371645-bib-0086] Coşkun, N. , S. Sarıtaş , Y. Jaouhari , M. Bordiga , and S. Karav . 2024. “The Impact of Freeze Drying on Bioactivity and Physical Properties of Food Products.” Applied Sciences 14: 9183. 10.3390/app14209183.

[fsn371645-bib-0087] Cruz Neto, J. P. R. , M. O. de Luna Freire , D. E. de Albuquerque Lemos , et al. 2024. “Targeting Gut Microbiota With Probiotics and Phenolic Compounds in the Treatment of Atherosclerosis: A Comprehensive Review.” Food 13: 2886. 10.3390/foods13182886. PMC11431284 39335815

[fsn371645-bib-0088] Cucu, A.‐A. , A. C. Urcan , O. Bobiș , et al. 2024. “Preliminary Identification and Quantification of Individual Polyphenols in *Fallopia japonica* Plants and Honey and Their Influence on Antimicrobial and Antibiofilm Activities.” Plants 13: 1883. 10.3390/plants13131883. 38999722 PMC11244575

[fsn371645-bib-0089] Dalet, J. T. , J. K. T. Narag , A. V. Hallare , and F. T. Heralde . 2023. “Effects of Apis Dorsata Honey on the MRNA Expression of Selected CYP450, Pro‐Apoptotic, and Anti‐Apoptotic Genes During Induced Cytotoxicity in Cyclophosphamide‐Treated Human Lung Carcinoma (A549) Cells.” Acta Medica Philippina 58: 37. 10.47895/amp.vi0.7600. PMC11586291 39600658

[fsn371645-bib-0090] Damayanti, E. , H. Nirwati , and J. Widada . 2024. “The Evaluation of Antimicrobial, Antioxidant, and Antiproliferative Activity of Stingless Bee Honey (Heterotrigona Itama and *Tetragonula laeviceps* ) as Functional Food.” In AIP Conference Proceedings, 060025. AIP Publishing LLC.

[fsn371645-bib-0091] Darwis, A. F. , A. Syahputra , E. Sufarnap , and E. W. S. Harahap . 2024. “The Comparison of Sumatera Forest Honey and Triamcinolone ACETONIDE for Traumatic Ulcer Healing in WISTAR Rat: Clinical and Histological Evaluation.” Journal of Health and Translational Medicine (JUMMEC) 27: 326–331.

[fsn371645-bib-0092] Das, N. , N. Ray , A. R. Patil , et al. 2022. “Inhibitory Effect of Selected Indian Honey on Colon Cancer Cell Growth by Inducing Apoptosis and Targeting the β‐Catenin/Wnt Pathway.” Food & Function 13: 8283–8303. 10.1039/D1FO03727G. 35834215

[fsn371645-bib-0093] De Gregorio, P. R. , A. Gennari , C. V. Nied , G. Volpato , and C. F. Volken de Souza . 2023. “Production of Oligosaccharides, a Prebiotic From Lactose, Using β‐Galactosidase.” In Enzymes Beyond Traditional Applications in Dairy Science and Technology; Elsevier, 383–401. Academic Press.

[fsn371645-bib-0094] de Jesús Romero‐Prado, M. M. , J. A. Curiel‐Beltrán , M. V. Miramontes‐Espino , E. G. Cardona‐Muñoz , A. Rios‐Arellano , and L. Balam‐Salazar . 2015. “Dietary Flavonoids Added to Pharmacological Antihypertensive Therapy Are Effective in Improving Blood Pressure.” Basic & Clinical Pharmacology & Toxicology 117: 57–64. 10.1111/bcpt.12360. 25441094

[fsn371645-bib-0095] Demir Kanbur, E. , T. Yuksek , V. Atamov , and A. E. Ozcelik . 2021. “A Comparison of the Physicochemical Properties of Chestnut and Highland Honey: The Case of Senoz Valley in the Rize Province of Turkey.” Food Chemistry 345: 128864. 10.1016/j.foodchem.2020.128864. 33601663

[fsn371645-bib-0096] Derewiaka, D. , E. Majewska , and P. Pruszkowska . 2024. “The Effects of Bee Additives on the Physico‐Chemical and Antioxidant Properties of Rapeseed Honey.” Applied Sciences 14: 1292. 10.3390/app14031292.

[fsn371645-bib-0097] Derya Andeden, M. , P. Altın Çelik , M. Çakır , R. Üzen , and H. Altuntaş . 2024. “Effects of Pervari Honey From Türkiye on Proliferation, Oxidative Stress, and Apoptosis of Human Breast Cancer Cells.” Journal of Advance Research in Applied Science 10: 627–639. 10.28979/jarnas.1456528.

[fsn371645-bib-0098] Dhiman, M. , S. Ghosh , T. G. Singh , S. Chauhan , P. Roy , and D. Lahiri . 2024. “Exploring the Potential of an Aloe Vera and Honey Extract Loaded bi‐Layered Nanofibrous Scaffold of PCL‐Col and PCL‐SBMA Mimicking the Skin Architecture for the Treatment of Diabetic Wounds.” Journal of Materials Chemistry B 12: 10383–10408. 10.1039/D4TB01469C. 39290135

[fsn371645-bib-0099] Diab, T. A. , T. Donia , and K. M. Saad‐Allah . 2021. “Characterization, Antioxidant, and Cytotoxic Effects of Some Egyptian Wild Plant Extracts.” Beni‐Suef University Journal of Basic and Applied Sciences 10: 13. 10.1186/s43088-021-00103-0.

[fsn371645-bib-0100] Didaras, N. A. , K. Karatasou , T. G. Dimitriou , G. D. Amoutzias , and D. Mossialos . 2020. “Antimicrobial Activity of Bee‐Collected Pollen and Beebread: State of the Art and Future Perspectives.” Antibiotics 9: 811. 10.3390/antibiotics9110811. 33202560 PMC7697837

[fsn371645-bib-0101] Djebli, N. , A. Yagoub , G. S. Oskay , and N. Arda . 2024. “Chestnut ‘Castanea Sativa Mill.’ Honey Potential in Preventing Memory Decline on Alzheimer's Disease Model Mice: Vitro Enzyme Activities, Behavioural, Memorial and Histopathological Studies.”

[fsn371645-bib-0102] Duman, H. , E. Akdaşçi , F. Eker , M. Bechelany , and S. Karav . 2024. “Gold Nanoparticles: Multifunctional Properties, Synthesis, and Future Prospects.” Nanomaterials 14: 1805. 10.3390/nano14221805. 39591046 PMC11597081

[fsn371645-bib-0103] Duman, H. , F. Eker , E. Akdaşçi , A. M. Witkowska , M. Bechelany , and S. Karav . 2024. “Silver Nanoparticles: A Comprehensive Review of Synthesis Methods and Chemical and Physical Properties.” Nanomaterials 14: 1527. 10.3390/nano14181527. 39330683 PMC11434896

[fsn371645-bib-0104] Duman, H. , M. Kaplan , A. Arslan , et al. 2021. “Potential Applications of Endo‐β‐N‐Acetylglucosaminidases From *Bifidobacterium longum* Subspecies Infantis in Designing Value‐Added, Next‐Generation Infant Formulas.” Frontiers in Nutrition 8: 646275. 33898500 10.3389/fnut.2021.646275 PMC8063050

[fsn371645-bib-0105] Duman, H. , and S. Karav . 2025. “Fiber and the Gut Microbiome and Its Impact on Inflammation.” In Nutrition in the Control of Inflammation, 51–76. Elsevier.

[fsn371645-bib-0106] Dumas, M.‐E. , R. H. Barton , A. Toye , et al. 2006. “Metabolic Profiling Reveals a Contribution of Gut Microbiota to Fatty Liver Phenotype in Insulin‐Resistant Mice.” Proceedings of the National Academy of Sciences 103: 12511–12516. 10.1073/pnas.0601056103. PMC1567909 16895997

[fsn371645-bib-0107] Durmishi, B. , V. Knights , I. Mehmeti , et al. 2023. “Determining the Quality of Honey in the Region of KOSOVA With Physiochemical Analysis.” Uludağ Aricilik Dergisi 23: 202–214. 10.31467/uluaricilik.1349616.

[fsn371645-bib-0108] Dzani, M. S. B. , and H. K. R. Nair . 2024. “Managing Diabetic Foot Ulcer With Osteomyletis Changes Using Medical Grade Honey and Foam Dressing A Case Report.” IWII 6th Glob. Wound Conf.

[fsn371645-bib-0109] Dżugan, M. , E. Ciszkowicz , M. Tomczyk , M. Miłek , and K. Lecka‐Szlachta . 2024. “Coniferous Honeydew Honey: Antibacterial Activity and Anti‐Migration Properties Against Breast Cancer Cell Line (MCF‐7).” Applied Sciences 14: 710. 10.3390/app14020710.

[fsn371645-bib-0110] Ebadi, P. , and M. Fazeli . 2021. “Evaluation of the Potential In Vitro Effects of Propolis and Honey on Wound Healing in Human Dermal Fibroblast Cells.” South African Journal of Botany 137: 414–422. 10.1016/j.sajb.2020.10.003.

[fsn371645-bib-0111] Eisa, A. , M. F. Elshal , S. Muawia , and H. Khalil . 2024. “The Combination of Sitagliptin and Bee Honey Extract Potentiates the Anti‐Proliferative Properties of 5‐Fluorouracil on Caco‐2 Cell Line Without Detectable Inflammatory Events.” Clinical Traditional Medicine and Pharmacology 5: 200165. 10.1016/j.ctmp.2024.200165.

[fsn371645-bib-0112] Eker, F. , E. Akdaşçi , H. Duman , M. Bechelany , and S. Karav . 2024. “Gold Nanoparticles in Nanomedicine: Unique Properties and Therapeutic Potential.” Nanomaterials 14: 1854. 10.3390/nano14221854. 39591094 PMC11597456

[fsn371645-bib-0113] Eker, F. , E. Bolat , B. Pekdemir , H. Duman , and S. L. Karav . 2023. “Neuroprotection Against Parkinson's Disease and Secondary Molecule for Potential Treatment.” Frontiers in Aging Neuroscience 15: 904. 10.3389/fnagi.2023.1204149. PMC10508234 37731953

[fsn371645-bib-0114] Eker, F. , H. Duman , E. Akdaşçi , A. M. Witkowska , M. Bechelany , and S. Karav . 2024. “Silver Nanoparticles in Therapeutics and Beyond: A Review of Mechanism Insights and Applications.” Nanomaterials 14: 1618. 10.3390/nano14201618. 39452955 PMC11510578

[fsn371645-bib-0115] Eko, S. N. , I. Arya , M. Rima , M. R. Mochamad , and W. J. Ririh . 2025. “Kuncara Bayu Rachmad Indonesian Honey as a Natural Remedy: Accelerating Wound Healing in Staphylococcus‐Infected Mice.” Jurnal Teknologi Laboratorium 1: 1–11. 10.29238/teknolabjournal.v14i1.394.

[fsn371645-bib-0116] El‐Aarag, B. , S. B. Shehata , I. M. El‐Garawani , H. R. El‐Seedi , and A. E. Nofal . 2024. “Regulation of Oxidative Stress and Apoptosis in Streptozotocin‐Induced Diabetic Rats by Egyptian Sidr Honey.” Chemistry & Biodiversity 21: e202400351. 10.1002/cbdv.202400351. 38717108

[fsn371645-bib-0117] Elfar, S. A. , I. M. Bahgat , M. A. Shebl , M. Lihoreau , and M. M. Tawfik . 2023. “Intraspecific Variability in Proteomic Profiles and Biological Activities of the Honey Bee Hemolymph.” Insects 14: 365. 10.3390/insects14040365. 37103179 PMC10142140

[fsn371645-bib-0118] Elsayed, T. R. , E. Nour , A. A. Hamed , A. A.‐M. Hassan , and Y. E. Elenany . 2024. “The Influence of Lactobacillus Spp. Secondary Metabolites Isolated From Immature Egyptian Honey on Human Pathogens, Transcription of Virulence Genes and Lung Cancer.” Indian Journal of Microbiology 64: 671–682. 10.1007/s12088-024-01224-7. 39011000 PMC11246380

[fsn371645-bib-0119] Emin Duru, M. , B. Eroğlu , G. Tel‐Çayan , et al. 2023. “HPLC‐DAD Analysis and Versatile Bioactivities of Turkish Sunflower Honeys Using Chemometric Approaches.” Chemistry & Biodiversity 20: e202300486. 10.1002/cbdv.202300486. 37192321

[fsn371645-bib-0120] Eming, S. A. , T. Krieg , and J. M. Davidson . 2007. “Inflammation in Wound Repair: Molecular and Cellular Mechanisms.” Journal of Investigative Dermatology 127: 514–525. 10.1038/sj.jid.5700701. 17299434

[fsn371645-bib-0121] Erejuwa, O. , S. Sulaiman , and M. Wahab . 2014. “Effects of Honey and Its Mechanisms of Action on the Development and Progression of Cancer.” Molecules 19: 2497–2522. 10.3390/molecules19022497. 24566317 PMC6270987

[fsn371645-bib-0122] Erejuwa, O. O. , S. A. Sulaiman , M. S. Ab Wahab , K. N. S. Sirajudeen , S. Salleh , and S. Gurtu . 2012. “Honey Supplementation in Spontaneously Hypertensive Rats Elicits Antihypertensive Effect via Amelioration of Renal Oxidative Stress.” Oxidative Medicine and Cellular Longevity 20: 1–14. 10.1155/2012/374037. PMC3270456 22315654

[fsn371645-bib-0123] Erejuwa, O. O. , S. A. Sulaiman , and M. S. A. Wahab . 2011. “Oligosaccharides Might Contribute to the Antidiabetic Effect of Honey: A Review of the Literature.” Molecules 17: 248–266. 10.3390/molecules17010248. 22205091 PMC6268503

[fsn371645-bib-0124] Erejuwa, O. O. , S. A. Sulaiman , M. S. A. Wahab , K. N. S. Sirajudeen , M. S. M. Salleh , and S. Gurtu . 2010. “Antioxidant Protective Effect of Glibenclamide and Metformin in Combination With Honey in Pancreas of Streptozotocin‐Induced Diabetic Rats.” International Journal of Molecular Sciences 11: 2056–2066. 10.3390/ijms11052056. 20559501 PMC2885093

[fsn371645-bib-0125] Eteraf‐Oskouei, T. , and M. Najafi . 2021. “Uses of Natural Honey in Cancer: An Updated Review.” Advanced Pharmaceutical Bulletin 12: 248. 10.34172/apb.2022.026. 35620330 PMC9106964

[fsn371645-bib-0126] Fadzil, M. A. M. , S. Mustar , and A. A. Rashed . 2023. “The Potential Use of Honey as a Neuroprotective Agent for the Management of Neurodegenerative Diseases.” Nutrients 15: 1558. 10.3390/nu15071558. 37049399 PMC10096917

[fsn371645-bib-0127] Faúndez, X. , M. E. Báez , J. Martínez , M. C. Zúñiga‐López , J. Espinoza , and E. Fuentes . 2023. “Evaluation of the Generation of Reactive Oxygen Species and Antibacterial Activity of Honey as a Function of Its Phenolic and Mineral Composition.” Food Chemistry 426: 136561. 10.1016/j.foodchem.2023.136561. 37321119

[fsn371645-bib-0128] Fauzi, A. N. , M. N. Norazmi , and N. S. Yaacob . 2011. “Tualang Honey Induces Apoptosis and Disrupts the Mitochondrial Membrane Potential of Human Breast and Cervical Cancer Cell Lines.” Food and Chemical Toxicology 49: 871–878. 10.1016/j.fct.2010.12.010. 21167897

[fsn371645-bib-0129] Francesko, A. , P. Petkova , and T. Tzanov . 2019. “Hydrogel Dressings for Advanced Wound Management.” Current Medicinal Chemistry 25: 5782–5797. 10.2174/0929867324666170920161246. 28933299

[fsn371645-bib-0130] Fratianni, F. , G. Amato , A. d'Acierno , et al. 2023. “In Vitro Prospective Healthy and Nutritional Benefits of Different Citrus Monofloral Honeys.” Scientific Reports 13: 1088. 10.1038/s41598-023-27802-1. 36658323 PMC9852249

[fsn371645-bib-0131] Fratianni, F. , G. Amato , M. N. Ombra , V. De Feo , F. Nazzaro , and B. De Giulio . 2024. “Chemical Characterization and Biological Properties of Leguminous Honey.” Antioxidants 13: 482. 10.3390/antiox13040482. 38671929 PMC11047671

[fsn371645-bib-0132] Fratianni, F. , B. De Giulio , A. d'Acierno , et al. 2023. “In Vitro Prebiotic Effects and Antibacterial Activity of Five Leguminous Honeys.” Food 12: 3338. 10.3390/foods12183338. PMC10529961 37761047

[fsn371645-bib-0133] Gad, S. F. , M. A. Mohamed , M. V. Asaad , et al. 2025. “To What Extent Garlic and Royal Honey Mitigating the Gastric Toxicity Induced by Bisphenol A in Adult Albino Rats: Histological, Immunohistochemical and Scanning Electron Microscopic Study.” Benha Medizinhistorisches Journal 42: 117–132. 10.21608/bmfj.2025.390708.2449.

[fsn371645-bib-0134] Gallo, C. , J. Girón‐Hernández , D. A. Honey , et al. 2024. “Synergistic Nanocoating With Layer‐By‐Layer Functionalized PCL Membranes Enhanced by Manuka Honey and Essential Oils for Advanced Wound Healing.” Scientific Reports 14: 20715. 10.1038/s41598-024-71466-4. 39237556 PMC11377730

[fsn371645-bib-0135] Gandini, M. , A. Cerullo , and G. Giusto . 2024. “Pectin‐Honey Hydrogel to Prevent Laparotomy Surgical Site Infection in Horses: A Pilot Study.” Journal of Equine Veterinary Science 139: 105128. 10.1016/j.jevs.2024.105128. 38852926

[fsn371645-bib-0136] Ganesan, K. , and B. Xu . 2017. “A Critical Review on Polyphenols and Health Benefits of Black Soybeans.” Nutrients 9: 455. 10.3390/nu9050455. 28471393 PMC5452185

[fsn371645-bib-0137] Gharibzahedi, S. M. T. , and S. M. Jafari . 2017. “The Importance of Minerals in Human Nutrition: Bioavailability, Food Fortification, Processing Effects and Nanoencapsulation.” Trends in Food Science and Technology 62: 119–132. 10.1016/j.tifs.2017.02.017.

[fsn371645-bib-0138] Ghasemi, M. , F. Sadeghimahalli , H. Jamali , A. Yazdi , M. Reza , and S. Moqadam . 2025. “Thyme Honey Reduced Hyperglycemia in Male Rats Subjected to Chronic Unpredictable Mild Stress: Possible Involvement of GLUT4 Protein and Circulating Irisin.” Avicenna Journal of Phytomedicine 14: 1379–1390. 10.22038/ajp. PMC12244951 40656624

[fsn371645-bib-0139] Gheldof, N. , X.‐H. Wang , and N. J. Engeseth . 2002. “Identification and Quantification of Antioxidant Components of Honeys From Various Floral Sources.” Journal of Agricultural and Food Chemistry 50: 5870–5877. 10.1021/jf0256135. 12358452

[fsn371645-bib-0140] Ghramh, H. A. , S. A. Alrumman , I. Ahmad , et al. 2023. “Chemical Characterization of Honey and Its Effect (Alone as Well as With Synthesized Silver Nanoparticles) on Microbial Pathogens' and Human Cancer Cell Lines' Growth.” Nutrients 15: 684. 10.3390/nu15030684. 36771391 PMC9919140

[fsn371645-bib-0141] Gibson, G. R. , K. P. Scott , R. A. Rastall , et al. 2010. “Dietary Prebiotics: Current Status and New Definition.” Food Sci. Technol. Bull. Funct. Foods 7: 1–19. 10.1616/1476-2137.15880.

[fsn371645-bib-0142] Gomes, S. , L. G. Dias , L. L. Moreira , P. Rodrigues , and L. Estevinho . 2010. “Physicochemical, Microbiological and Antimicrobial Properties of Commercial Honeys From Portugal.” Food and Chemical Toxicology 48: 544–548. 10.1016/j.fct.2009.11.029. 19909782

[fsn371645-bib-0143] Gouda, Z. , S. Abdi , S. Al Majid , et al. 2015. “Honey on the Healing of Foot Ulcers: A Case Series. Index Wounds.” 25855854

[fsn371645-bib-0144] Guiné, R. P. F. , S. G. Florença , P. M. R. Correia , O. Anjos , C. Coelho , and C. A. H. Costa . 2022. “Bee (*Apis mellifera* L.) Broods: Composition, Technology and Gastronomic Applicability.” Food 11: 2750. 10.3390/foods11182750. PMC9497570 36140877

[fsn371645-bib-0145] Guo, H. , Y. Kouzuma , and M. Yonekura . 2009. “Structures and Properties of Antioxidative Peptides Derived From Royal Jelly Protein.” Food Chemistry 113: 238–245. 10.1016/j.foodchem.2008.06.081.

[fsn371645-bib-0146] Guo, Q. , Y. Jin , X. Chen , et al. 2024. “NF‐ΚB in Biology and Targeted Therapy: New Insights and Translational Implications.” Signal Transduction and Targeted Therapy 9: 53. 10.1038/s41392-024-01757-9. 38433280 PMC10910037

[fsn371645-bib-0147] Haddadin, M. S. Y. , I. N. S. Jamal Abu , and R. K. Robinson . 2007. “Effect of Honey on the Growth and Metabolism of Two Bacterial Species of Intestinal Origin.” Pakistan the Journal of Nutrition 6: 693–697. 10.3923/pjn.2007.693.697.

[fsn371645-bib-0148] Haines, R. R. , S. Xi , K. J. Green , and K. A. Hammer . 2024. “In Vitro Activity of Western Australian Honeys and Manuka Honey Against Clinically Important Yeasts.” Yeast 41: 537–548. 10.1002/yea.3974. 39032089

[fsn371645-bib-0149] Hajian‐Tilaki, A. , R. E. Kenari , R. Razavi , and R. Farahmandfar . 2024. “Phenolic Profile, Antioxidant Properties, and Pollen Spectra of Iranian‐Originated Honeys.” European Food Research and Technology 250: 2317–2329. 10.1007/s00217-024-04539-3.

[fsn371645-bib-0150] Hamadou, W. S. , N. Bouali , E. B. Alhejaili , et al. 2023. “Acacia Honey‐Derived Bioactive Compounds Exhibit Induction of P53‐Dependent Apoptosis in the MCF‐7 Human Breast Cancer Cell Line.” Pharmacognosy Magazine 19: 144–155. 10.1177/09731296221145076.

[fsn371645-bib-0151] Han, C. Y. 2016. “Roles of Reactive Oxygen Species on Insulin Resistance in Adipose Tissue.” Diabetes and Metabolism Journal 40: 272. 10.4093/dmj.2016.40.4.272. 27352152 PMC4995181

[fsn371645-bib-0152] Harbane, S. , O. Escuredo , Y. Saker , et al. 2024. “The Contribution of Botanical Origin to the Physicochemical and Antioxidant Properties of Algerian Honeys.” Food 13: 573. 10.3390/foods13040573. PMC10888090 38397550

[fsn371645-bib-0153] Hasam, S. , D. Qarizada , and M. Azizi . 2020. “A Review: Honey and Its Nutritional Composition.” Asian Journal of Research in Biochemistry 7: 34–43. 10.9734/ajrb/2020/v7i330142.

[fsn371645-bib-0154] Hashim, K.‐N. , K.‐Y. Chin , and F. Ahmad . 2021. “The Mechanism of Honey in Reversing Metabolic Syndrome.” Molecules 26: 808. 10.3390/molecules26040808. 33557218 PMC7913905

[fsn371645-bib-0155] Hasim, H. , S. Salam , P. V. Rao , S. Muthuraju , N. A. Che Jalil , and M. A. Asari . 2024. “Effect of Tualang Honey‐Mediated Silver Nanoparticles on TNF‐α Level, Caspase‐3 Activity and Hippocampal Morphology in Kainic Acid‐Induced Neurodegeneration in Male Rats.” IIUM Medical Journal Malaysia 23: 4. 10.31436/imjm.v23i04.2223.

[fsn371645-bib-0156] Hasim, H. , S. Q. A. Suhaimi , C. B. A. Aziz , T. W. Yaw , and S. K. Hassan . 2020. “Comparison of Antinociceptive and Antioxidative Effects of Tualang Honey and Vitamin C in a Rat Model of Inflammatory Pain.” Indian Journal of Natural Products and Resources 11: 52–59. 10.56042/ijnpr.v11i1.24665.

[fsn371645-bib-0157] Hasitha Maduranga Karunarathne, W. A. , S. Na , M.‐H. Lee , C.‐H. Kang , Y. H. Choi , and G.‐Y. Kim . 2024. “ *Hovenia dulcis* Thunb. Monofloral Honey Attenuates LPS‐Induced Inflammation and Endotoxemia Through the Activation of the Nrf2/HO‐1 Axis.” Journal of Traditional and Complementary Medicine 15: 647–658. 10.1016/j.jtcme.2024.09.003. 41169942 PMC12570105

[fsn371645-bib-0158] Hayes, J. D. , A. T. Dinkova‐Kostova , and K. D. Tew . 2020. “Oxidative Stress in Cancer.” Cancer Cell 38: 167–197. 10.1016/j.ccell.2020.06.001. 32649885 PMC7439808

[fsn371645-bib-0159] Hernanz, D. , M. J. Jara‐Palacios , J. L. Santos , A. Gómez Pajuelo , F. J. Heredia , and A. Terrab . 2023. “The Profile of Phenolic Compounds by HPLC‐MS in Spanish Oak (Quercus) Honeydew Honey and Their Relationships With Color and Antioxidant Activity.” LWT 180: 114724. 10.1016/j.lwt.2023.114724.

[fsn371645-bib-0160] Holubová, A. , L. Chlupáčová , J. Krocová , et al. 2023. “The Use of Medical Grade Honey on Infected Chronic Diabetic Foot Ulcers—A Prospective Case‐Control Study.” Antibiotics 12: 1364. 10.3390/antibiotics12091364. 37760661 PMC10525154

[fsn371645-bib-0161] Hossain, M. L. , L. Y. Lim , K. Hammer , D. Hettiarachchi , and C. Locher . 2022. “A Review of Commonly Used Methodologies for Assessing the Antibacterial Activity of Honey and Honey Products.” Antibiotics 11: 975. 10.3390/antibiotics11070975. 35884229 PMC9312033

[fsn371645-bib-0162] Hosseini, S. A. , S. Noruzi , P. Kesharwani , and A. Sahebkar . 2025. “Hydrogel‐Based Dressing for Wound Healing: A Systematic Review of Clinical Trials.” International Journal of Biological Macromolecules 308: 142322. 10.1016/j.ijbiomac.2025.142322. 40118421

[fsn371645-bib-0163] Huyop, F. , N. Huda , R. Ab Wahab , et al. 2024. “Green Honey of Banggi Island: A Preliminary Anti‐Diabetic Study on Zebrafish Model.” Heliyon 10: e26469. 10.1016/j.heliyon.2024.e26469. 38404777 PMC10884957

[fsn371645-bib-0164] Idriss, I. , A. H. Ali , A. Alam , et al. 2024. “Differential In Vitro Cytotoxic Effects and Metabolomic Insights Into Raw and Powdered Manuka Honey Through UPLC‐Q‐TOF‐MS.” Scientific Reports 14: 17551. 10.1038/s41598-024-68387-7. 39079967 PMC11289323

[fsn371645-bib-0165] Ifeoma, A. V. , I. E. P. Kenneth , A. O. Abel , and O. P. Ngozi . 2024. “Ofiri Pascal Ngozi Antifungal Potential of Honey Against Dermatophytes: A Comprehensive Study on Isolates From Children and Farmers in Wukari, North East Nigeria.” International Journal of Advanced Biological and Biomedical Research 12: 206–217.

[fsn371645-bib-0166] Iftikhar, A. , R. Nausheen , M. Khurshid , et al. 2023. “Pancreatic Regenerative Potential of Manuka Honey Evidenced Through Pancreatic Histology and Levels of Transcription Factors in Diabetic Rat Model.” Heliyon 9: e20017. 10.1016/j.heliyon.2023.e20017. 37809953 PMC10559747

[fsn371645-bib-0167] Iglesias, M. T. , C. De Lorenzo , M. Del Carmen Polo , P. J. Martín‐Alvarez , and E. Pueyo . 2004. “Usefulness of Amino Acid Composition to Discriminate Between Honeydew and Floral Honeys. Application to Honeys From a Small Geographic Area.” Journal of Agricultural and Food Chemistry 52: 84–89. 10.1021/jf030454q. 14709017

[fsn371645-bib-0168] Ingawale, D. K. , S. K. Mandlik , and S. S. Patel . 2015. “An Emphasis on Molecular Mechanisms of Anti‐Inflammatory Effects and Glucocorticoid Resistance.” Journal of Complementary and Integrative Medicine 12: 1–13. 10.1515/jcim-2014-0051. 25503867

[fsn371645-bib-0169] Iosageanu, A. , L. M. Stefan , O. Craciunescu , and A. Cimpean . 2024. “Anti‐Inflammatory and Wound Healing Properties of Different Honey Varieties From Romania and Correlations to Their Composition.” Life 14: 1187. 10.3390/life14091187. 39337969 PMC11432766

[fsn371645-bib-0170] Irish, J. , S. Blair , and D. A. Carter . 2011. “The Antibacterial Activity of Honey Derived From Australian Flora.” PLoS One 6: e18229. 10.1371/journal.pone.0018229. 21464891 PMC3065476

[fsn371645-bib-0171] Irish, J. , D. A. Carter , T. Shokohi , and S. E. Blair . 2006. “Honey Has an Antifungal Effect Against Candida Species.” Medical Mycology 44: 289–291. 10.1080/13693780500417037. 16702110

[fsn371645-bib-0172] Jaganathan, S. K. , and M. Mandal . 2009. “Antiproliferative Effects of Honey and of Its Polyphenols: A Review.” BioMed Research International 2009: 1–13. 10.1155/2009/830616. PMC2712839 19636435

[fsn371645-bib-0173] Jan Mei, S. , M. S. Mohd Nordin , and A. S. Norrakiah . 2010. “Fructooligosaccharides in Honey and Effects of Honey on Growth of *Bifidobacterium longum* BB 536.” International Food Research Journal 17: 557–561.

[fsn371645-bib-0174] Jeeva, J. , J. Sunitha , R. Ananthalakshmi , S. Rajkumari , M. Ramesh , and R. Krishnan . 2015. “Enzymatic Antioxidants and Its Role in Oral Diseases.” Journal of Pharmacy & Bioallied Sciences 7: 331. 10.4103/0975-7406.163438. PMC4606614 26538872

[fsn371645-bib-0175] Jeong, Y. H. , W. Li , H. J. Yang , et al. 2024. “Ethyl Acetate Fraction of Chestnut Honey Attenuates Scopolamine‐Induced Cognitive Impairment in Mice and Glutamate‐Induced Neurotoxicity in HT22 Cells.” Antioxidants 13: 1346. 10.3390/antiox13111346. 39594488 PMC11591166

[fsn371645-bib-0176] Jiang, L. , M. Xie , G. Chen , J. Qiao , H. Zhang , and X. Zeng . 2020. “Phenolics and Carbohydrates in Buckwheat Honey Regulate the Human Intestinal Microbiota.” Evidence‐Based Complementary and Alternative Medicine 2020: 6432942. 10.1155/2020/6432942. 32184894 PMC7061112

[fsn371645-bib-0177] Jiang, W. , J. Paolini , D. Bereau , et al. 2023. “French Guiana Honeys From the Amazon Biome: First Description of Volatile Fraction and Antioxidant Capacity.” Heliyon 9: e18526. 10.1016/j.heliyon.2023.e18526. 37554807 PMC10404971

[fsn371645-bib-0178] Jiang, X. , A. Lin , S. Li , et al. 2022. “Effects of Artificial Honey and Epigallocatechin‐3‐Gallate on Streptococcus Pyogenes .” BMC Microbiology 22: 207. 10.1186/s12866-022-02611-0. 36028794 PMC9419396

[fsn371645-bib-0179] Jilo, K. R. 2024. “Stingless Bee (Meliponulla Baccaerii) Honey Antibacterial Activities Against *Salmonella typhi* , *Escherichia coli* , Staphylococcus Aureus and *Enterococcus faecalis* .” Journal of Bacteriology Parasitology 15: 506.

[fsn371645-bib-0180] 2001. “Jozef Simúth Some Properties of the Main Protein of Honeybee ( *Apis mellifera* ) Royal Jelly.” Apidologie 32: 69–80. 10.1051/apido:2001112.

[fsn371645-bib-0181] Julianti, E. , K. K. Rajah , and I. Fidrianny . 2017. “Antibacterial Activity of Ethanolic Extract of Cinnamon Bark, Honey, and Their Combination Effects Against Acne‐Causing Bacteria.” Scientia Pharmaceutica 85: 19. 10.3390/scipharm85020019. 28398231 PMC5489923

[fsn371645-bib-0182] Kajiwara, S. , H. Gandhi , and Z. Ustunol . 2002. “Effect of Honey on the Growth of and Acid Production by Human Intestinal Bifidobacterium spp.: An In Vitro Comparison With Commercial Oligosaccharides and Inulin.” Journal of Food Protection 65: 214–218. 10.4315/0362-028X-65.1.214. 11808799

[fsn371645-bib-0183] Kamakura, M. 2011. “Royalactin Induces Queen Differentiation in Honeybees.” Nature 473: 478–483. 10.1038/nature10093. 21516106

[fsn371645-bib-0184] Kannan, K. , and S. K. Jain . 2000. “Oxidative Stress and Apoptosis.” Pathophysiology 7: 153–163. 10.1016/S0928-4680(00)00053-5. 10996508

[fsn371645-bib-0185] Kaplan, M. , A. S. Şahutoğlu , S. Sarıtaş , et al. 2022. “Role of Milk Glycome in Prevention, Treatment, and Recovery of COVID‐19.” Frontiers in Nutrition 9: 1033779. 10.3389/fnut.2022.1033779. 36424926 PMC9680090

[fsn371645-bib-0186] Karami, Z. , and B. Akbari‐adergani . 2019. “Bioactive Food Derived Peptides: A Review on Correlation Between Structure of Bioactive Peptides and Their Functional Properties.” Journal of Food Science and Technology 56: 535–547. 10.1007/s13197-018-3549-4. 30906011 PMC6400753

[fsn371645-bib-0187] Karapetsas, A. , G.‐P. Voulgaridou , D. Iliadi , et al. 2020. “Honey Extracts Exhibit Cytoprotective Properties Against UVB‐Induced Photodamage in Human Experimental Skin Models.” Antioxidants 9: 566. 10.3390/antiox9070566. 32629798 PMC7402120

[fsn371645-bib-0188] Karav, S. , G. Casaburi , A. Arslan , M. Kaplan , B. Sucu , and S. Frese . 2019. “N‐Glycans From Human Milk Glycoproteins Are Selectively Released by an Infant Gut Symbiont In Vivo.” Journal of Functional Foods 61: 103485. 10.1016/j.jff.2019.103485.

[fsn371645-bib-0189] Karav, S. , and A. Ekşi . 2013. “Antioxidant Capacity and Total Phenolic Contents of Peach and Apricot Cultivars Harvested From Different Regions of Turkey.”

[fsn371645-bib-0190] Karav, S. , A. Le Parc , L. Nobrega , et al. 2016. “Oligosaccharides Released From Milk Glycoproteins Are Selective Growth Substrates for Infant‐Associated Bifidobacteria.” Applied and Environmental Microbiology 82: 3622–3630. 10.1128/AEM.00547-16. 27084007 PMC4959171

[fsn371645-bib-0191] Karav, S. 2018. “Selective Deglycosylation of Lactoferrin to Understand Glycans' Contribution to Antimicrobial Activity of Lactoferrin.” Cellular and Molecular Biology 64: 52–57. 10.14715/cmb/2018.64.9.8. 30030954

[fsn371645-bib-0192] Karav, S. , A. O. Arikal , and A. Eksi . 2015. “Apple Peel Is a Promising Source of Natural Bioactive Compounds That Promote Human Health.” Journal of Food and Nutrition Research 3: 624–628.

[fsn371645-bib-0193] Karbasi, S. , A. H. Asadian , E. Azaryan , M. Naseri , and A. Zarban . 2024. “Quantitative Analysis of Biochemical Characteristics and Anti‐Cancer Properties in MCF‐7 Breast Cancer Cell Line: A Comparative Study Between Ziziphus Jujube Honey and Commercial Honey.” Molecular Biology Reports 51: 344. 10.1007/s11033-024-09219-9. 38400882

[fsn371645-bib-0194] Kassim, M. , M. Achoui , M. R. Mustafa , M. A. Mohd , and K. M. Yusoff . 2010. “Ellagic Acid, Phenolic Acids, and Flavonoids in Malaysian Honey Extracts Demonstrate In Vitro Anti‐Inflammatory Activity.” Nutrition Research 30: 650–659. 10.1016/j.nutres.2010.08.008. 20934607

[fsn371645-bib-0195] Kassym, L. , A. Kussainova , Y. Semenova , and P. McLoone . 2024. “Antimicrobial Effect of Honey Phenolic Compounds Against E. coli—An In Vitro Study.” Pharmaceuticals 17: 560. 10.3390/ph17050560. 38794130 PMC11123796

[fsn371645-bib-0196] Kaźmierczak‐Barańska, J. , and B. T. Karwowski . 2024. “The Antioxidant Potential of Commercial Manuka Honey From New Zealand—Biochemical and Cellular Studies.” Current Issues in Molecular Biology 46: 6366–6376. 10.3390/cimb46070380. 39057022 PMC11275220

[fsn371645-bib-0197] Keskin, M. , G. Kaya , S. Bayram , A. Kurek‐Górecka , and P. Olczyk . 2023. “Green Synthesis, Characterization, Antioxidant, Antibacterial and Enzyme Inhibition Effects of Chestnut ( *Castanea sativa* ) Honey‐Mediated Silver Nanoparticles.” Molecules 28: 2762. 10.3390/molecules28062762. 36985734 PMC10055715

[fsn371645-bib-0198] Khalil, M. , and S. Sulaiman . 2010. “The Potential Role of Honey and Its Polyphenols in Preventing Heart Disease: A Review.” African Journal of Traditional, Complementary, and Alternative Medicines 7: 315. 10.4314/ajtcam.v7i4.56693. PMC3005390 21731163

[fsn371645-bib-0199] Khalil, M. I. , M. Mahaneem , S. M. S. Jamalullail , N. Alam , and S. A. Sulaiman . 2011. “Evaluation of Radical Scavenging Activity and Colour Intensity of Nine Malaysian Honeys of Different Origin.” Journal ApiProduct and ApiMedical Science 3: 4–11. 10.3896/IBRA.4.03.1.02.

[fsn371645-bib-0200] Khalil, M. I. , S. A. Sulaiman , and L. Boukraa . 2010. “Antioxidant Properties of Honey and Its Role in Preventing Health Disorder.” Open Nutraceuticals Journal 3: 6–16. 10.2174/18763960010030100006.

[fsn371645-bib-0201] Khattabi, L. , M. Dakkach , and H. Bouziane . 2023. “Allouch M Physicochemical Properties, Antioxidant, and Antibacterial Activity of Argania Spinosa Honey Produced Only in Morocco: Application in the Care of Surgical Wounds.” Moroccan Journal of Chemistry 11: 1038–1056.

[fsn371645-bib-0202] Khitan, Z. , and D. H. Kim . 2013. “Fructose: A Key Factor in the Development of Metabolic Syndrome and Hypertension.” Journal of Nutrition and Metabolism 2013: 1–12. 10.1155/2013/682673. PMC3677638 23762544

[fsn371645-bib-0203] Kim, B. , Y. Kim , E. Kwon , et al. 2025. “ *Hovenia dulcis* Honey Suppresses Androgen‐Induced Epithelial–Mesenchymal Transition in Benign Prostatic Hyperplasia.” Food Frontiers 6: 1362–1375. 10.1002/fft2.70026.

[fsn371645-bib-0204] Kim, H.‐J. , B.‐R. Jin , C.‐D. Lee , et al. 2024. “Anti‐Inflammatory Effect of Chestnut Honey and Cabbage Mixtures Alleviates Gastric Mucosal Damage.” Nutrients 16: 389. 10.3390/nu16030389. 38337674 PMC10857084

[fsn371645-bib-0205] Kim, J. , P. Durai , D. Jeon , et al. 2018. “Phloretin as a Potent Natural TLR2/1 Inhibitor Suppresses TLR2‐Induced Inflammation.” Nutrients 10: 868. 10.3390/nu10070868. 29976865 PMC6073418

[fsn371645-bib-0206] Koch, W. , J. Zagórska , M. Michalak‐Tomczyk , S. Karav , and A. Wawruszak . 2024. “Plant Phenolics in the Prevention and Therapy of Acne: A Comprehensive Review.” Molecules 29: 4234. 10.3390/molecules29174234. 39275081 PMC11397085

[fsn371645-bib-0207] Kolayli, S. , L. Boukraâ , H. Sahin , and F. Abdellah . 2012. “Sugars in Honey.” In Dietary Sugars: Chemistry, Analysis, Function and Effects, 3–15. Royal Society of Chemistry.

[fsn371645-bib-0208] Koloh, R. , V. L. Balázs , L. Nagy‐Radványi , et al. 2024. “Chestnut Honey Is Effective Against Mixed Biofilms at Different Stages of Maturity.” Antibiotics 13: 255. 10.3390/antibiotics13030255. 38534690 PMC10967288

[fsn371645-bib-0209] Koodathil, J. , G. Venkatachalam , and K. Bhaskaran . 2023. “In Vitro and In Vivo Antidiabetic Activity of Bitter Honey in Streptozotocin‐Nicotinamide‐Induced Diabetic Wistar Rats.” Journal of Medicine and Life 16: 91–100. 10.25122/jml-2022-0099. 36873120 PMC9979185

[fsn371645-bib-0210] Kowalska, G. , J. Rosicka‐Kaczmarek , K. Miśkiewicz , et al. 2024. “Influence of Novel Microcapsulates of Bee Products on Gut Microbiota Modulation and Their Prebiotic and Pro‐Adhesive Properties.” Molecules 29: 2751. 10.3390/molecules29122751. 38930817 PMC11206356

[fsn371645-bib-0211] Kumari, I. , Y. A. Hajam , K. Thiyagarajan , A. Giri , and R. Kumar . 2023. “Evaluation of Antioxidant and Antibacterial Potential of Honey Produced From Stimulative Diet Fed Bee Colonies.” Discover Sustainability 4: 21. 10.1007/s43621-023-00135-9.

[fsn371645-bib-0212] Kuroda, K. , and G. A. Caputo . 2013. “Antimicrobial Polymers as Synthetic Mimics of Host‐Defense Peptides.” WIREs Nanomedicine and Nanobiotechnology 5: 49–66. 10.1002/wnan.1199. 23076870

[fsn371645-bib-0213] Kuropatnicki, A. K. , M. Kłósek , and M. Kucharzewski . 2018. “Honey as Medicine: Historical Perspectives.” Journal of Apicultural Research 57: 113–118. 10.1080/00218839.2017.1411182.

[fsn371645-bib-0214] Kwakman, P. H. S. , and S. A. J. Zaat . 2012. “Antibacterial Components of Honey.” IUBMB Life 64: 48–55. 10.1002/iub.578. 22095907

[fsn371645-bib-0215] Kwon, E.‐B. , Y. S. Kim , B. Kim , et al. 2023. “Korean Chestnut Honey Suppresses HSV‐1 Infection by Regulating the ROS–NLRP3 Inflammasome Pathway.” Antioxidants 12: 1935. 10.3390/antiox12111935. 38001788 PMC10669648

[fsn371645-bib-0216] Lakhmili, H. , K. Warda , A. El‐Abbassi , and A. Hafidi . 2024. “Antioxidant and Anti‐Glycation Activity of Eight Moroccan Honeys From Different Botanical Origins.” Discover Food 4: 6. 10.1007/s44187-024-00074-y.

[fsn371645-bib-0217] Lane, J. A. , J. Calonne , H. Slattery , and R. M. Hickey . 2019. “Oligosaccharides Isolated From MGO Manuka Honey Inhibit the Adhesion of *Pseudomonas aeruginosa* , *Escherichia coli* O157:H7 and *Staphylococcus aureus* to Human HT‐29 Cells.” Food 8: 446. 10.3390/foods8100446. PMC6835506 31581550

[fsn371645-bib-0218] Lawag, I. L. , M. K. Islam , T. Sostaric , L. Y. Lim , K. Hammer , and C. Locher . 2023. “Antioxidant Activity and Phenolic Compound Identification and Quantification in Western Australian Honeys.” Antioxidants 12: 189. 10.3390/antiox12010189. 36671051 PMC9854687

[fsn371645-bib-0219] Lei, K. , J. Fang , G. Wang , and X. Pang . 2025. “Advances in Multifunctional Diagnostic Hydrogels for Complex Chronic Wound Healing and Monitoring.” Sensors & Diagnostics 4: 642–668. 10.1039/D5SD00051C.

[fsn371645-bib-0220] Lemmen, G. , A. Hudson , and J. Rasmus . 2024. “Growth Inhibition of *Escherichia coli* and Staphylococcus Epidermidis From Various Types of Honey.” Journal of Immune Based Therapies and Vaccines Encompasses 13: 47–54. 10.4236/jibtva.2024.134004.

[fsn371645-bib-0221] León‐Ruiz, V. , S. Vera , A. V. González‐Porto , and M. P. San Andrés . 2013. “Analysis of Water‐Soluble Vitamins in Honey by Isocratic RP‐HPLC.” Food Analytical Methods 6: 488–496. 10.1007/s12161-012-9477-4.

[fsn371645-bib-0222] Li, C.‐P. , S.‐Y. Gau , C.‐C. Chen , C.‐H. Kao , R.‐Y. Tsai , and H.‐J. Yang . 2024. “Honey in Alleviating Severe Oral Mucositis Among Head and Neck Cancer Patients Undergoing Radiation Therapy.” In Vivo (Brooklyn) 38: 1397–1404. 10.21873/invivo.13581. PMC11059902 38688612

[fsn371645-bib-0223] Li, M. , H. Xiao , Y. Su , et al. 2023. “Synergistic Inhibitory Effect of Honey and *Lactobacillus plantarum* on Pathogenic Bacteria and Their Promotion of Healing in Infected Wounds.” Pathogens 12: 501. 10.3390/pathogens12030501. 36986423 PMC10053434

[fsn371645-bib-0224] Liang, X. , C. Huang , H. Liu , et al. 2024. “Natural Hydrogel Dressings in Wound Care: Design, Advances, and Perspectives.” Chinese Chemical Letters 35: 109442. 10.1016/j.cclet.2023.109442.

[fsn371645-bib-0225] Libby, P. 2002. “Inflammation in Atherosclerosis.” Nature 420: 868–874. 10.1038/nature01323. 12490960

[fsn371645-bib-0226] Liu, J.‐R. , Y.‐L. Ye , T.‐Y. Lin , Y.‐W. Wang , and C.‐C. Peng . 2013. “Effect of Floral Sources on the Antioxidant, Antimicrobial, and Anti‐Inflammatory Activities of Honeys in Taiwan.” Food Chemistry 139: 938–943. 10.1016/j.foodchem.2013.02.015. 23561193

[fsn371645-bib-0227] Liu, M. , X. Yao , G. Zhang , et al. 2023. “Physicochemical Properties, Chemical Constituents, and Antioxidant Activity of *Eurya* Honey.” International Journal of Food Science and Technology 58: 3458–3468. 10.1111/ijfs.16386.

[fsn371645-bib-0228] Liu, N. , H. Shen , F. Zhang , et al. 2023. “Applications and Prospects of Functional Oligosaccharides in Pig Nutrition: A Review.” Animal Nutrition 13: 206–215. 10.1016/j.aninu.2023.02.002. 37388461 PMC10300388

[fsn371645-bib-0229] Liu, X. , J. Xu , B. Zhang , et al. 2019. “The Reciprocal Regulation Between Host Tissue and Immune Cells in Pancreatic Ductal Adenocarcinoma: New Insights and Therapeutic Implications.” Molecular Cancer 18: 184. 10.1186/s12943-019-1117-9. 31831007 PMC6909567

[fsn371645-bib-0230] Lv, Z. , J. Song , Y. Xiang , et al. 2024. “Structural Characterization and Therapeutic Effect of Alhagi Honey Oligosaccharide on Liver Fibrosis in Mice.” Fitoterapia 175: 105974. 10.1016/j.fitote.2024.105974. 38663563

[fsn371645-bib-0231] Magoshi, I. B. , A. W. Nekhumbe , M. A. Ibrahim , J. C. Serem , and M. J. Bester . 2023. “Gastrointestinal Effects on the Antioxidant and Immunomodulatory Properties of South African Fynbos Honey.” International Journal of Food Science 2023: 1–13. 10.1155/2023/2553197. PMC10691895 38045104

[fsn371645-bib-0232] Mahmod, Z. , M. F. Zulkifli , M. A. A. Masimen , W. I. W. Ismail , M. A. Sharifudin , and K. A. M. Amin . 2025. “Investigating the Efficacy of Gellan Gum Hydrogel Films Infused With Acacia Stingless Bee Honey in Wound Healing.” International Journal of Biological Macromolecules 296: 139753. 10.1016/j.ijbiomac.2025.139753. 39800021

[fsn371645-bib-0233] Main, E. N. , J. C. Huang , and G. L. Bowlin . 2024. “Methyl Syringate: A Primary Driving Factor in Manuka Honeys Ability to Ameliorate Neutrophil Intracellular ROS Activity and NETosis.” Frontiers in Bioscience 29: 255. 10.31083/j.fbl2907255. PMC11973827 39082351

[fsn371645-bib-0234] Majtan, J. 2014. “Honey: An Immunomodulator in Wound Healing.” Wound Repair and Regeneration 22: 187–192. 10.1111/wrr.12117. 24612472

[fsn371645-bib-0235] Majtan, J. , M. Sojka , H. Palenikova , M. Bucekova , and V. Majtan . 2020. “Vitamin C Enhances the Antibacterial Activity of Honey Against Planktonic and Biofilm‐Embedded Bacteria.” Molecules 25: 992. 10.3390/molecules25040992. 32102181 PMC7070301

[fsn371645-bib-0236] Malkoc, M. A. , S. Ö. Yaman , Ş. Ersöz , and S. Kolaylı . 2025. “Bee‐Derived Antioxidants as a Protective Strategy Against Doxorubicin‐Induced Ovarian Damage.” Chemistry & Biodiversity 22: e00766. 10.1002/cbdv.202500766. 40694649 PMC12629153

[fsn371645-bib-0237] Mano, M. C. R. , P. N. dos Santos , B. N. Paulino , and G. Molina . 2022. “Enzyme Technology Applied to Biomolecule Synthesis for the Food Industry.” In Value‐Addition in Food Products and Processing Through Enzyme Technology, 57–69. Elsevier.

[fsn371645-bib-0238] Márquez‐Garbán, D. C. , C. D. Yanes , G. Llarena , et al. 2024. “Manuka Honey Inhibits Human Breast Cancer Progression in Preclinical Models.” Nutrients 16: 2369. 10.3390/nu16142369. 39064812 PMC11279598

[fsn371645-bib-0239] Martinotti, S. , G. Bonsignore , M. Patrone , and E. Ranzato . 2025. “Correlation Between Honey Parameters and Wound Healing Properties: The Case of Piedmont (Italy) Samples.” Current Pharmaceutical Biotechnology 26: 302–311. 10.2174/0113892010328741240828093859. 39238381

[fsn371645-bib-0240] Martinotti, S. , G. Bonsignore , and E. Ranzato . 2024. “Understanding the Anticancer Properties of Honey.” International Journal of Molecular Sciences 25: 11724. 10.3390/ijms252111724. 39519281 PMC11547017

[fsn371645-bib-0241] Martinotti, S. , G. Pellavio , M. Patrone , U. Laforenza , and E. Ranzato . 2020. “Manuka Honey Induces Apoptosis of Epithelial Cancer Cells Through Aquaporin‐3 and Calcium Signaling.” Life 10: 256. 10.3390/life10110256. 33120979 PMC7692226

[fsn371645-bib-0242] Martinotti, S. , and E. Ranzato . 2018. “Honey, Wound Repair and Regenerative Medicine.” Journal of Functional Biomaterial 9: 34. 10.3390/jfb9020034. PMC6023338 29738478

[fsn371645-bib-0243] Masfufatun, M. , B. Setiawan , R. Purbowati , et al. 2024. “Anti‐Biofilm Properties of Clover Honey Against Candida Albican.” Healthc. Low‐Resource Settings 12: 1–13. 10.4081/hls.2024.11988.

[fsn371645-bib-0244] Masri, S. , S. Aksoy , H. Duman , S. Karav , H. M. Kayili , and B. Salih . 2024. “Distinguishing Turkish Pine Honey From Multi‐Floral Honey Through MALDI‐MS‐Based N‐Glycomics and Machine Learning.” Journal of Food Measurement and Characterization 18: 5673–5682. 10.1007/s11694-024-02597-5.

[fsn371645-bib-0245] Mavric, E. , S. Wittmann , G. Barth , and T. Henle . 2008. “Identification and Quantification of Methylglyoxal as the Dominant Antibacterial Constituent of Manuka ( *Leptospermum scoparium* ) Honeys From New Zealand.” Molecular Nutrition & Food Research 52: 483–489. 10.1002/mnfr.200700282. 18210383

[fsn371645-bib-0246] McLoone, P. , T. O. Oladejo , L. Kassym , and G. J. McDougall . 2024. “Honey Phytochemicals: Bioactive Agents With Therapeutic Potential for Dermatological Disorders.” Phytherapy Research 38: 5741–5764. 10.1002/ptr.8330. 39324175

[fsn371645-bib-0247] Mduda, C. A. , J. M. Hussein , and M. H. Muruke . 2023. “The Effects of Bee Species and Vegetation on the Antioxidant Properties of Honeys Produced by Afrotropical Stingless Bees (Hymenoptera, Apidae, Meliponini).” Journal of Agriculture and Food Research 14: 100736. 10.1016/j.jafr.2023.100736.

[fsn371645-bib-0248] Megha, K. , X. Joseph , V. Akhil , and P. Mohanan . 2021. “Cascade of Immune Mechanism and Consequences of Inflammatory Disorders.” Phytomedicine 91: 153712. 10.1016/j.phymed.2021.153712. 34511264 PMC8373857

[fsn371645-bib-0249] Mello dos Santos, M. , N. Khan , L. Y. Lim , and C. Locher . 2024. “Antioxidant Activity, Physicochemical and Sensory Properties of Stingless Bee Honey From Australia.” Food 13: 1657. 10.3390/foods13111657. PMC11171737 38890884

[fsn371645-bib-0250] Melnik, B. 2015. “Linking Diet to Acne Metabolomics, Inflammation, and Comedogenesis: An Update.” Clinical, Cosmetic and Investigational Dermatology 15: 371–388. 10.2147/CCID.S69135. PMC4507494 26203267

[fsn371645-bib-0251] Meo, S. A. , S. A. Al‐Asiri , A. L. Mahesar , and M. J. Ansari . 2017. “Role of Honey in Modern Medicine.” Saudi Journal of Biological Sciences 24: 975–978. 10.1016/j.sjbs.2016.12.010. 28663690 PMC5478293

[fsn371645-bib-0252] Michelle, C. , and A. Lorna . 2018. “Honey for Wound Management: A Review of Clinical Effectiveness and Guidelines; Canadian Agency for Drugs and Technologies in Health: Ottowa.” 30855767

[fsn371645-bib-0253] Minden‐Birkenmaier, B. A. , and G. L. Bowlin . 2018. “Honey‐Based Templates in Wound Healing and Tissue Engineering.” Bioengineering 5: 46. 10.3390/bioengineering5020046. 29903998 PMC6027142

[fsn371645-bib-0254] Mohammed, S. E. A. , M. El‐Niweiri , H. Ghramh , et al. 2024. “Antimicrobial, Antioxidant and Cytotoxic Properties Oof Four Types of Honey as Related to Their Phenolic and Flavonoid Contents.”

[fsn371645-bib-0255] Mohan, A. , S.‐Y. Quek , N. Gutierrez‐Maddox , Y. Gao , and Q. Shu . 2017. “Effect of Honey in Improving the Gut Microbial Balance.” Food Quality and Safety 1: 107–115. 10.1093/fqsafe/fyx015.

[fsn371645-bib-0256] Mohd Nasir, S. , A. F. Ismail , T. S. Tuan Ismail , et al. 2024. “Hepatic and Renal Effects of Oral Stingless Bee Honey in a Streptozotocin‐Induced Diabetic Rat Model.” World the Journal of Experimental Medicine 14: 91271. 10.5493/wjem.v14.i1.91271. PMC10999067 38590306

[fsn371645-bib-0257] Molan, P. C. 1992. “The Antibacterial Activity of Honey.” Bee World 73: 5–28. 10.1080/0005772X.1992.11099109.

[fsn371645-bib-0258] Moniruzzaman, M. , M. Khalil , S. Sulaiman , and S. Gan . 2011. “Advances in the Analytical Methods for Determining the Antioxidant Properties of Honey: A Review.” African Journal of Traditional, Complementary, and Alternative Medicines 9: 36–42. 10.4314/ajtcam.v9i1.5. PMC3746522 23983317

[fsn371645-bib-0259] Moussa, A. , D. Noureddine , A. Saad , M. Abdelmelek , and B. Abdelkader . 2012. “Antifungal Activity of Four Honeys of Different Types From Algeria Against Pathogenic Yeast: Candida Albicans and Rhodotorula Sp.” Asian Pacific Journal of Tropical Biomedicine 2: 554–557. 10.1016/S2221-1691(12)60096-3. 23569970 PMC3609343

[fsn371645-bib-0260] Mu, W. , S. Yu , L. Zhu , T. Zhang , and B. Jiang . 2012. “Recent Research on 3‐Phenyllactic Acid, a Broad‐Spectrum Antimicrobial Compound.” Applied Microbiology and Biotechnology 95: 1155–1163. 10.1007/s00253-012-4269-8. 22782253

[fsn371645-bib-0261] Naqvi, F. , N. Dastagir , and A. Jabeen . 2023. “Honey Proteins Regulate Oxidative Stress, Inflammation and Ameliorates Hyperglycemia in Streptozotocin Induced Diabetic Rats.” BMC Complementary Medicine and Therapies 23: 14. 10.1186/s12906-023-03837-9. 36653816 PMC9847130

[fsn371645-bib-0262] Narag, J. K. T. , J. Dalet , and F. Heralde . 2023. “20P Effects of *Apis dorsata* Honey on the Expression of Selected CYP450, Pro‐Apoptotic, and Anti‐Apoptotic Genes During Induced Cytotoxicity in Cyclophosphamide‐Treated Human Lung Carcinoma (A549) Cells.” ESMO Open 8: 101666. 10.1016/j.esmoop.2023.101666. PMC11586291 39600658

[fsn371645-bib-0263] Narayanan, K. B. , and R. Bhaskar . 2025. “Innovations in Designing Hydrogels for Advanced Wound Dressing Applications: An Editorial Review.” Gels 11: 332. 10.3390/gels11050332. 40422351 PMC12111174

[fsn371645-bib-0264] Naskar, A. , K. Chatterjee , K. Roy , et al. 2024. “Mechanistic Roles of Different Varieties of Honey on Wound Healing: Recent Update.” Journal of Pharmacology and Pharmacotherapeutics 15: 5–18. 10.1177/0976500X241237361.

[fsn371645-bib-0265] Navarro‐Hortal, M. D. , J. M. Romero‐Márquez , J. Ansary , et al. 2025. “Honey as a Neuroprotective Agent: Molecular Perspectives on Its Role in Alzheimer's Disease.” Nutrients 17: 2577. 10.3390/nu17162577. 40871605 PMC12388817

[fsn371645-bib-0266] Nayan, N. S. , M. A. M. Yazid , K. Nallappan , et al. 2020. “In Vitro Modulation of Endogenous Antioxidant Enzyme Activities and Oxidative Stress in Autism Lymphoblastoid Cell Line (ALCL) by Stingless Bee Honey Treatment.” Oxidative Medicine and Cellular Longevity 2020: 1–7. 10.1155/2020/4539891. PMC7723473 33335642

[fsn371645-bib-0267] Nazzaro, F. , M. N. Ombra , F. Coppola , et al. 2024. “Antibacterial Activity and Prebiotic Properties of Six Types of Lamiaceae Honey.” Antibiotics 13: 868. 10.3390/antibiotics13090868. 39335041 PMC11428214

[fsn371645-bib-0268] Nguyen, H. T. L. , N. Panyoyai , S. Kasapis , E. Pang , and N. Mantri . 2019. “Honey and Its Role in Relieving Multiple Facets of Atherosclerosis.” Nutrients 11: 167. 10.3390/nu11010167. 30646548 PMC6356546

[fsn371645-bib-0269] Nguyen, T.‐H. , and D. Haltrich . 2013. “Microbial Production of Prebiotic Oligosaccharides.” In Microbial Production of Food Ingredients, Enzymes and Nutraceuticals, 494–530. Elsevier.

[fsn371645-bib-0270] Nogueira‐Rio, N. , L. Varela Vazquez , A. Lopez‐Santamarina , A. Mondragon‐Portocarrero , S. Karav , and J. M. Miranda . 2024. “Mobile Applications and Artificial Intelligence for Nutrition Education: A Narrative Review.” Dietetics 3: 483–503. 10.3390/dietetics3040035.

[fsn371645-bib-0271] Noh, H. , and G. L. King . 2007. “The Role of Protein Kinase C Activation in Diabetic Nephropathy.” Kidney International 72: S49–S53. 10.1038/sj.ki.5002386. 17653211

[fsn371645-bib-0272] Nolan, V. C. , J. Harrison , and J. A. G. Cox . 2019. “Dissecting the Antimicrobial Composition of Honey.” Antibiotics 8: 251. 10.3390/antibiotics8040251. 31817375 PMC6963415

[fsn371645-bib-0273] Núñez‐Gómez, V. , M. San Mateo , L. Sánchez‐Martínez , and M. J. Periago . 2024. “Antibacterial Effect of Spanish Honeys of Different Botanical Origins Against *Staphylococcus epidermidis* .” International Journal of Molecular Sciences 25: 6590. 10.3390/ijms25126590. 38928296 PMC11203921

[fsn371645-bib-0274] Ode Sumarlin, L. , A. T. Nugraha , A. Muawanah , N. Ernita , and N. Amilia . 2023. “Characterization of the Compound of Longan Honey From Indonesia Using LC‐MS/MS and FTIR and the Mechanism of Inhibition of HEp‐2 Cells.” Journal of Research in Pharmacy 27, no. 5: 2035–2057. 10.29228/jrp.483.

[fsn371645-bib-0275] Ofor, C. C. , O. O. Erejuwa , G. C. Akuodor , D. O. Aja , A. U. Mba , and E. N. Shu . 2022. “The Role of Honey in the Treatment of Type 2 Diabetes Mellitus: A Review of Literature.” International Journal of Basic and Clinical Pharmacology 12: 120. 10.18203/2319-2003.ijbcp20223366.

[fsn371645-bib-0276] Okamoto, I. , Y. Taniguchi , T. Kunikata , et al. 2003. “Major Royal Jelly Protein 3 Modulates Immune Responses In Vitro and In Vivo.” Life Sciences 73: 2029–2045. 10.1016/S0024-3205(03)00562-9. 12899927

[fsn371645-bib-0277] Oryan, A. , E. Alemzadeh , and A. Moshiri . 2016. “Biological Properties and Therapeutic Activities of Honey in Wound Healing: A Narrative Review and Meta‐Analysis.” Journal of Tissue Viability 25: 98–118. 10.1016/j.jtv.2015.12.002. 26852154

[fsn371645-bib-0278] Othman, N. H. 2012. “Honey and Cancer: Sustainable Inverse Relationship Particularly for Developing Nations—A Review.” Evidence‐Based Complementary and Alternative Medicine 2012: 1–10. 10.1155/2012/410406. PMC3385631 22761637

[fsn371645-bib-0279] Ounjaijean, S. , S. Chaipoot , R. Phongphisutthinant , et al. 2024. “Evaluation of Prebiotic and Health‐Promoting Functions of Honeybee Brood Biopeptides and Their Maillard Reaction Conjugates.” Food 13: 2847. 10.3390/foods13172847. PMC11395396 39272610

[fsn371645-bib-0280] Pai, S. , C. Shivappa , and A. Surendra . 2018. “Anti‐Obesity and Anti‐Hyperlipidemic Activity of Processed Honey–A Randomised, Open Labeled, Controlled Clinical Study.” Journal of Research in Traditional Medicine 4: 40. 10.5455/JRTM.2018/816.

[fsn371645-bib-0281] Park, H. G. , B. Y. Kim , M. J. Park , et al. 2019. “Antibacterial Activity of Major Royal Jelly Proteins of the Honeybee ( *Apis mellifera* ) Royal Jelly.” Journal of Asia‐Pacific Entomology 22: 737–741. 10.1016/j.aspen.2019.06.005.

[fsn371645-bib-0282] Pekdemir, B. , H. Duman , A. Arslan , et al. 2022. “Immobilization of a Bifidobacterial Endo‐ß‐N‐Acetylglucosaminidase to Generate Bioactive Compounds for Food Industry.” Frontiers in Bioengineering and Biotechnology 10: 922423. 10.3389/fbioe.2022.922423. 35935492 PMC9353140

[fsn371645-bib-0283] Pekdemir, B. , and S. Karav . 2024. “Exploring the Diverse Biological Significance and Roles of Fucosylated Oligosaccharides.” Frontiers in Molecular Biosciences 11: 1403727. 10.3389/fmolb.2024.1403727. 38863964 PMC11165149

[fsn371645-bib-0284] Peter, A. , and M. D. Indelicato . 2018. Chemical Sterilization Techniques for Allograft Preparation for Anterior Cruciate Ligament Reconstruction, 117–120. Reconstruction and Basic Science.

[fsn371645-bib-0285] PFund Color Grading . 2024. “What is Colour Measuring in Honey?” https://Honey‐Ai.Com/En/Technology/Pfund‐Color‐Grading.

[fsn371645-bib-0286] Pleeging, C. C. F. , F. A. D. T. G. Wagener , H. de Rooster , and N. A. J. Cremers . 2022. “Revolutionizing Non‐Conventional Wound Healing Using Honey by Simultaneously Targeting Multiple Molecular Mechanisms.” Drug Resistance Updates 62: 100834. 10.1016/j.drup.2022.100834. 35427872

[fsn371645-bib-0287] Prieto, K. , Y. Cao , E. Mohamed , et al. 2019. “Polyphenol‐Rich Extract Induces Apoptosis With Immunogenic Markers in Melanoma Cells Through the ER Stress‐Associated Kinase PERK.” Cell Death Discov 5: 134. 10.1038/s41420-019-0214-2. 31531232 PMC6733947

[fsn371645-bib-0288] Qanash, H. , A. S. Bazaid , N. K. Binsaleh , M. Patel , O. W. Althomali , and B. Sheeha . 2023. “Bin In Vitro Antiproliferative Apoptosis Induction and Cell Cycle Arrest Potential of Saudi Sidr Honey Against Colorectal Cancer.” Nutrients 15: 3448. 10.3390/nu15153448. 37571386 PMC10421499

[fsn371645-bib-0289] Rahmani, A. H. , and A. Y. Babiker . 2025. “Review on Role of Honey in Disease Prevention and Treatment Through Modulation of Biological Activities.” Open Life Sciences 20: 20251069. 10.1515/biol-2025-1069. 40059876 PMC11889511

[fsn371645-bib-0290] Ramanathan, A. N. K. G. , A. J. Nair , and V. S. Sugunan . 2018. “A Review on Royal Jelly Proteins and Peptides.” Journal of Functional Foods 44: 255–264. 10.1016/j.jff.2018.03.008.

[fsn371645-bib-0291] Ramírez Miranda, I. , J. Acevedo‐Fernandez , E. Negrete‐Leon , D. Abram Betancur‐Ancona , and Y. Beatriz Moguel‐Ordoñez . 2024. “In Vivo Wound‐Healing and Anti‐Inflammatory Activities of Honey Produced by *Melipona beecheii* Bees.” Jundishapur Journal of Natural Pharmaceutical Products 19: e143682. 10.5812/jjnpp-143682.

[fsn371645-bib-0292] Ramli, N. Z. , K.‐Y. Chin , K. A. Zarkasi , and F. Ahmad . 2018. “A Review on the Protective Effects of Honey Against Metabolic Syndrome.” Nutrients 10: 1009. 10.3390/nu10081009. 30072671 PMC6115915

[fsn371645-bib-0293] Ranneh, Y. , A. M. Akim , H. A. Hamid , H. Khazaai , A. Fadel , and A. M. Mahmoud . 2019. “Stingless Bee Honey Protects Against Lipopolysaccharide Induced‐Chronic Subclinical Systemic Inflammation and Oxidative Stress by Modulating Nrf2, NF‐ΚB and P38 MAPK.” Nutrition & Metabolism (London) 16: 15. 10.1186/s12986-019-0341-z. PMC6391794 30858869

[fsn371645-bib-0294] Ranneh, Y. , A. M. Akim , H. A. Hamid , et al. 2021. “Honey and Its Nutritional and Anti‐Inflammatory Value.” BMC Complementary Medicine and Therapies 21: 30. 10.1186/s12906-020-03170-5. 33441127 PMC7807510

[fsn371645-bib-0295] Ranneh, Y. , F. Ali , A. M. Akim , H. A. Hamid , H. Khazaai , and A. Fadel . 2017. “Crosstalk Between Reactive Oxygen Species and Pro‐Inflammatory Markers in Developing Various Chronic Diseases: A Review.” Applied Biological Chemistry 60: 327–338. 10.1007/s13765-017-0285-9.

[fsn371645-bib-0296] Ranneh, Y. , A. M. Mahmoud , A. Fadel , et al. 2021. “Acute Inflammation and Oxidative Stress Induced by Lipopolysaccharide and the Ameliorative Effect of Stingless Bee Honey.” Combinatorial Chemistry & High Throughput Screening 24: 744–757. 10.2174/1386207323999200918152111. 32957878

[fsn371645-bib-0297] Rao, P. V. , K. T. Krishnan , N. Salleh , and S. H. Gan . 2016. “Biological and Therapeutic Effects of Honey Produced by Honey Bees and Stingless Bees: A Comparative Review.” Revista Brasileira de Farmacognosia 26: 657–664. 10.1016/j.bjp.2016.01.012.

[fsn371645-bib-0298] Rasad, H. , M. H. Entezari , E. Ghadiri , B. Mahaki , and N. Pahlavani . 2018. “The Effect of Honey Consumption Compared With Sucrose on Lipid Profile in Young Healthy Subjects (Randomized Clinical Trial).” Clin. Nutr. ESPEN 26: 8–12. 10.1016/j.clnesp.2018.04.016. 29908688

[fsn371645-bib-0299] Rawlings, N. D. 2020. “Twenty‐Five Years of Nomenclature and Classification of Proteolytic Enzymes.” Biochimica et Biophysica Acta, Proteins and Proteomics 1868: 140345. 10.1016/j.bbapap.2019.140345. 31838087

[fsn371645-bib-0300] Razif, R. , N. I. M. Fadilah , M. Maarof , D. Looi Qi Hao , A. P. Y. Wen , and M. B. Fauzi . 2025. “Physicochemical Characterization of Injectable Genipin‐Crosslinked Gelatin–Kelulut Honey Hydrogels for Future Cutaneous Tissue Loss.” Polymers (Basel) 17: 1129. 10.3390/polym17091129. 40362913 PMC12073527

[fsn371645-bib-0301] Reaven, G. M. 1988. “Role of Insulin Resistance in Human Disease.” Diabetes 37: 1595–1607. 10.2337/diab.37.12.1595. 3056758

[fsn371645-bib-0302] Ren, C. , Q. Li , T. Luo , et al. 2023. “Antioxidant Polyphenols From Lespedeza Bicolor Turcz. Honey: Anti‐Inflammatory Effects on Lipopolysaccharide‐Treated RAW 264.7 Macrophages.” Antioxidants 12: 1809. 10.3390/antiox12101809. 37891888 PMC10604429

[fsn371645-bib-0303] Reuter, S. , S. C. Gupta , M. M. Chaturvedi , and B. B. Aggarwal . 2010. “Oxidative Stress, Inflammation, and Cancer: How Are They Linked?” Free Radical Biology & Medicine 49: 1603–1616. 10.1016/j.freeradbiomed.2010.09.006. 20840865 PMC2990475

[fsn371645-bib-0304] Ribeiro, M. , M. Simões , C. Vitorino , and F. Mascarenhas‐Melo . 2024. “Hydrogels in Cutaneous Wound Healing: Insights Into Characterization, Properties, Formulation and Therapeutic Potential.” Gels 10: 188. 10.3390/gels10030188. 38534606 PMC10970251

[fsn371645-bib-0305] Rice‐Evans, C. A. , and N. J. Miller . 1996. “Antioxidant Activities of Flavonoids as Bioactive Components of Food.” Biochemical Society Transactions 24: 790–795. 10.1042/bst0240790. 8878849

[fsn371645-bib-0306] Rohlfing, C. L. , H.‐M. Wiedmeyer , R. R. Little , J. D. England , A. Tennill , and D. E. Goldstein . 2002. “Defining the Relationship Between Plasma Glucose and HbA1c.” Diabetes Care 25: 275–278. 10.2337/diacare.25.2.275. 11815495

[fsn371645-bib-0307] Romário‐Silva, D. , M. Franchin , S. M. Alencar , et al. 2024. “Antimicrobial and Antibiofilm Activities of Brazilian Organic Honey Against Oral Microorganisms.” Brazilian Journal of Microbiology 55: 2285–2292. 10.1007/s42770-024-01343-9. 38744770 PMC11405583

[fsn371645-bib-0308] Romero‐Márquez, J. M. , M. D. Navarro‐Hortal , F. J. Orantes , et al. 2023. “In Vivo Anti‐Alzheimer and Antioxidant Properties of Avocado ( *Persea americana* Mill.) Honey From Southern Spain.” Antioxidants 12: 404. 10.3390/antiox12020404. 36829962 PMC9952156

[fsn371645-bib-0309] Rosendale, D. I. , I. S. Maddox , M. C. Miles , M. Rodier , M. Skinner , and J. Sutherland . 2008. “High‐Throughput Microbial Bioassays to Screen Potential New Zealand Functional Food Ingredients Intended to Manage the Growth of Probiotic and Pathogenic Gut Bacteria.” International Journal of Food Science and Technology 43: 2257–2267. 10.1111/j.1365-2621.2008.01863.x.

[fsn371645-bib-0310] Rossano, R. , M. Larocca , T. Polito , et al. 2012. “What Are the Proteolytic Enzymes of Honey and What They Do Tell us? A Fingerprint Analysis by 2‐D Zymography of Unifloral Honeys.” PLoS One 7: e49164. 10.1371/journal.pone.0049164. 23145107 PMC3492327

[fsn371645-bib-0311] Ruslee, S. S. , S. S. M. Zaid , I. H. Bakrin , Y. M. Goh , and N. M. Mustapha . 2020. “Protective Effect of Tualang Honey Against Cadmium‐Induced Morphological Abnormalities and Oxidative Stress in the Ovary of Rats.” BMC Complementary Medicine and Therapies 20: 160. 10.1186/s12906-020-02960-1. 32471398 PMC7260854

[fsn371645-bib-0312] Saad, B. 2025. “Immunomodulatory and Anti‐Inflammatory Properties of Honey and Bee Products.” Immuno 5: 19. 10.3390/immuno5020019.

[fsn371645-bib-0313] Saftić Martinović, L. , N. Birkic , T. Pavlešić , et al. 2024. “Chemical Characterization of Rare Unifloral Honeys of Ailanthus ( *Ailanthus altissima* ), Fennel (Foenicum Vulgare), and Raspberry ( *Rubus idaeus* ) and Their Antimicrobial and Antioxidant Activity.” Agricultural Research 14: 130–142. 10.1007/s40003-024-00754-2.

[fsn371645-bib-0314] Saha, A. , S. Chattopadhyay , M. Azam , and P. K. Sur . 2012. “The Role of Honey in Healing of Bedsores in Cancer Patients.” South Asian J. Cancer 1: 66–71. 10.4103/2278-330X.103714. 24455516 PMC3876612

[fsn371645-bib-0315] Şahutoğlu, A. S. , H. Duman , S. A. Frese , and S. Karav . 2020. “Structural Insights of Two Novel N‐Acetyl‐Glucosaminidase Enzymes Through In Silico Methods.” Turkish Journal of Chemistry 44: 1703–1712. 10.3906/kim-2006-19. 33488263 PMC7763110

[fsn371645-bib-0316] Salvo, J. , C. Sandoval , C. Schencke , F. Acevedo , and M. del Sol . 2023. “Healing Effect of a Nano‐Functionalized Medical‐Grade Honey for the Treatment of Infected Wounds.” Pharmaceutics 15: 2187. 10.3390/pharmaceutics15092187. 37765158 PMC10536296

[fsn371645-bib-0317] Samat, S. , F. Kanyan Enchang , F. Nor Hussein , and W. I. Wan Ismail . 2017. “Four‐Week Consumption of Malaysian Honey Reduces Excess Weight Gain and Improves Obesity‐Related Parameters in High Fat Diet Induced Obese Rats.” Evidence‐Based Complementary and Alternative Medicine 2017: 1342150. 10.1155/2017/1342150. 28246535 PMC5299215

[fsn371645-bib-0318] Samat, S. , M. A. M. Salleh , Z. Adam , W. Iryani , and W. Ismail . 2019. “Pineapple Honey Inhibits Adipocytes Proliferation and Reduces Lipid Droplet Accumulation in 3T3‐L1 Adipocytes.”

[fsn371645-bib-0319] Sánchez, M. , M. Galisteo , R. Vera , et al. 2006. “Quercetin Downregulates NADPH Oxidase, Increases ENOS Activity and Prevents Endothelial Dysfunction in Spontaneously Hypertensive Rats.” Journal of Hypertension 24: 75–84. 10.1097/01.hjh.0000198029.22472.d9. 16331104

[fsn371645-bib-0320] Sánchez‐Martín, V. , P. Morales , A. Iriondo‐DeHond , et al. 2023. “Differential Apoptotic Effects of Bee Product Mixtures on Normal and Cancer Hepatic Cells.” Antioxidants 12: 615. 10.3390/antiox12030615. 36978864 PMC10045410

[fsn371645-bib-0321] Santos Filipe, M. , T. Kowalczyk , W. Kukula‐Koch , et al. 2024. “Evaluating the Quality, Physicochemical Properties, and Biological Activities of Centauri Honey From Turkey.” Food Bioscience 62: 105028. 10.1016/j.fbio.2024.105028.

[fsn371645-bib-0322] Sanz, M. L. , N. Polemis , V. Morales , et al. 2005. “In Vitro Investigation Into the Potential Prebiotic Activity of Honey Oligosaccharides.” Journal of Agricultural and Food Chemistry 53: 2914–2921. 10.1021/jf0500684. 15826039

[fsn371645-bib-0323] Saputra, S. H. , B. Saragih , I. W. Kusuma , and E. T. Arung . 2021. “Antioxidant and Antibacterial Screening of Honey of Hiterotrogona Itama Collected From Differents Meliponiculture Areas in East Kalimantan, Indonesia.” Nusantara Bioscience 13: 232–237. 10.13057/nusbiosci/n130213.

[fsn371645-bib-0324] Sarıtaş, S. , H. Duman , B. Pekdemir , J. M. Rocha , F. Oz , and S. Karav . 2024. “Functional Chocolate: Exploring Advances in Production and Health Benefits.” International Journal of Food Science and Technology 59: 5303–5325. 10.1111/ijfs.17312.

[fsn371645-bib-0325] Sarıtaş, S. , A. C. M. Portocarrero , J. M. Miranda López , et al. 2024. “The Impact of Fermentation on the Antioxidant Activity of Food Products.” Molecules 29: 3941. 10.3390/molecules29163941. 39203019 PMC11357363

[fsn371645-bib-0326] Saroğlu, Ö. , N. Ecem Bayram , and B. Özçelik . 2023. “Comparison of Bioactive Constituents by HPLC–DAD–ESI‐MS and UFLC and In Vitro Antioxidant Activities of Blossom Honey, Bee Pollen, and Propolis.” European Food Research and Technology 249: 3085–3096. 10.1007/s00217-023-04350-6.

[fsn371645-bib-0327] Scepankova, H. , P. Combarros‐Fuertes , J. M. Fresno , et al. 2021. “Role of Honey in Advanced Wound Care.” Molecules 26: 4784. 10.3390/molecules26164784. 34443372 PMC8398244

[fsn371645-bib-0328] Schell, K. R. , K. E. Fernandes , E. Shanahan , et al. 2022. “The Potential of Honey as a Prebiotic Food to Re‐Engineer the Gut Microbiome Toward a Healthy State.” Frontiers in Nutrition 9: 957932. 10.3389/fnut.2022.957932. 35967810 PMC9367972

[fsn371645-bib-0329] Schmidlová, S. , Z. Javůrková , B. Tremlová , et al. 2006. “Exploring the Influence of Soil Types on the Mineral Profile of Honey: Implications for Geographical Origin Prediction.” Food 2024: 13. 10.3390/foods13132006. PMC11241210 38998511

[fsn371645-bib-0330] Sejbuk, M. , I. Mirończuk‐Chodakowska , S. Karav , and A. M. Witkowska . 2024. “Dietary Polyphenols, Food Processing and Gut Microbiome: Recent Findings on Bioavailability, Bioactivity, and Gut Microbiome Interplay.” Antioxidants 13: 1220. 10.3390/antiox13101220. 39456473 PMC11505337

[fsn371645-bib-0331] Shaheran, A. A. , N. A. A. Suhaimi , W. A. N. W. Ahmad , S. H. Shamsuddin , J. M. Abdullah , and M. Z. Mustafa . 2025. “Antidepressant and Neuroprotective Potential of Stingless Bee Honey in a Preclinical Stress Model.” Journal of Functional Foods 130: 106913. 10.1016/j.jff.2025.106913.

[fsn371645-bib-0332] Shahid, H. , A. A. Shah , S. N. U. Shah Bukhari , et al. 2023. “Synthesis, Characterization, and Biological Properties of Iron Oxide Nanoparticles Synthesized From *Apis mellifera* Honey.” Molecules 28: 6504. 10.3390/molecules28186504. 37764280 PMC10534332

[fsn371645-bib-0333] Shaikh, A. , F. Ahmad , S. L. Teoh , J. Kumar , and M. F. Yahaya . 2024. “Unveiling the Therapeutic Potential of Kelulut (Stingless Bee) Honey in Alzheimer's Disease: Findings From a Rat Model Study.” Antioxidants 13: 926. 10.3390/antiox13080926. 39199172 PMC11351951

[fsn371645-bib-0334] Shakoori, Z. , E. Salaseh , A. R. Mehrabian , D. M. Tehrani , N. F. Dardashti , and F. Salmanpour . 2024. “The Amount of Antioxidants in Honey Has a Strong Relationship With the Plants Selected by Honey Bees.” Scientific Reports 14: 351. 10.1038/s41598-023-51099-9. 38172229 PMC10764931

[fsn371645-bib-0335] Shapiro, H. , P. Singer , Z. Halpern , and R. Bruck . 2007. “Polyphenols in the Treatment of Inflammatory Bowel Disease and Acute Pancreatitis.” Gut 56: 426–436. 10.1136/gut.2006.094599. 16931577 PMC1856830

[fsn371645-bib-0336] Sharma, S. , A. Chauhan , and E. S. Okeke . 2024. “Honey as Potential Cosmeceutical Agent and Functional Food.” In Honey in Food Science and Physiology, 57–87. Singapore.

[fsn371645-bib-0337] Shi, H. , M. V. Kokoeva , K. Inouye , I. Tzameli , H. Yin , and J. S. Flier . 2006. “TLR4 Links Innate Immunity and Fatty Acid–Induced Insulin Resistance.” Journal of Clinical Investigation 116: 3015–3025. 10.1172/JCI28898. 17053832 PMC1616196

[fsn371645-bib-0338] Shin, H.‐S. , and Z. Ustunol . 2005. “Carbohydrate Composition of Honey From Different Floral Sources and Their Influence on Growth of Selected Intestinal Bacteria: An In Vitro Comparison.” Food Research International 38: 721–728. 10.1016/j.foodres.2005.01.007.

[fsn371645-bib-0339] Singh, S. , A. Young , and C.‐E. McNaught . 2017. “The Physiology of Wound Healing.” Surgery 35: 473–477. 10.1016/j.mpsur.2017.06.004.

[fsn371645-bib-0340] Sirhandi, B. R. , P. Goswami , S. Memon , and N. Qazi . 2024. “Barkha Goswami Histomorphological Alterations of Wound Healing by Honey in Comparison With Vitamin C in Male Albino Mice.” Rawal Medical Journal 48: 785.

[fsn371645-bib-0341] Soares, S. , J. S. Amaral , M. B. P. P. Oliveira , and I. Mafra . 2017. “A Comprehensive Review on the Main Honey Authentication Issues: Production and Origin.” Comprehensive Reviews in Food Science and Food Safety 16: 1072–1100. 10.1111/1541-4337.12278. 33371614

[fsn371645-bib-0342] Song, J. , X. Zhao , J. Bo , et al. 2024. “A Polysaccharide From Alhagi Honey Protects the Intestinal Barrier and Regulates the Nrf2/HO‐1‐TLR4/MAPK Signaling Pathway to Treat Alcoholic Liver Disease in Mice.” Journal of Ethnopharmacology 321: 117552. 10.1016/j.jep.2023.117552. 38072293

[fsn371645-bib-0343] Song, J. J. , and R. Salcido . 2011. “Use of Honey in Wound Care.” Advances in Skin & Wound Care 24: 40–44. 10.1097/01.ASW.0000392731.34723.06. 21150765

[fsn371645-bib-0344] Stavropoulou, E. , E. Ieronymaki , E. Dimitroulia , et al. 2022. “Anti‐Inflammatory and Antibacterial Effects and Mode of Action of Greek Arbutus, Chestnut, and Fir Honey in Mouse Models of Inflammation and Sepsis.” Microorganisms 10: 2374. 10.3390/microorganisms10122374. 36557628 PMC9784341

[fsn371645-bib-0345] Suhaimi, H. , N. Murdini , N. Shamsuddin , A. Ma'amor , and P. E. Abas . 2025. “Synthesis and Characterization of Honey‐Pectin Hydrogel: Exploring Potential Applications in Wound Healing.” Malaysian Journal of Fundamental and Applied Sciences 21: 1907–1927. 10.11113/mjfas.v21n2.3907.

[fsn371645-bib-0346] Sulaimon, F. A. , R. Y. Ibiyeye , A. Imam , et al. 2024a. “Honey and Levodopa Comparably Preserved Substantia Nigra Pars Compacta Neurons Through the Modulation of Nuclear Factor Erythroid 2‐Related Factor 2 Signaling Pathway in 1‐Methyl‐4‐Phenyl‐1,2,3,6‐Tetrahydropyridine‐Induced Parkinson's Disease Model.” Anatomy and Cell Biology 57: 431–445. 10.5115/acb.24.034. 38992924 PMC11424567

[fsn371645-bib-0347] Sulaimon, F. A. , R. Y. Ibiyeye , A. Imam , et al. 2024b. “Honey and Levodopa Comparably Prevented Oxidative Damage by Modulating ROS‐Nrf2‐GSH Pathway in MPTP‐Induced Parkinson's Disease.”

[fsn371645-bib-0348] Sultana, S. , K. Foster , T. Bates , et al. 2024. “Determination of Physicochemical Characteristics, Phytochemical Profile and Antioxidant Activity of Various Clover Honeys.” Chemistry & Biodiversity 21: e202301880. 10.1002/cbdv.202301880. 38494456

[fsn371645-bib-0349] Süntar, I. , S. Çetinkaya , E. Panieri , et al. 2021. “Regulatory Role of Nrf2 Signaling Pathway in Wound Healing Process.” Molecules 26: 2424. 10.3390/molecules26092424. 33919399 PMC8122529

[fsn371645-bib-0350] Suryadinata, K. L. , A. Basuki , A. Song , N. V. Yovita , A. P. Pakan , and A. E. Sagala . 2024. “Effect of Honey and Povidone‐Iodine on Acute Laceration Wound Healing: A Pilot Randomised Controlled Trial Study.” Journal of Wound Care 33: 570–576. 10.12968/jowc.2022.0020. 39137253

[fsn371645-bib-0351] Susilowati, F. , and M. N. Azkia . 2022. “Prebiotic Potential of Oligosaccharides: Vitro Study of Indonesian Local Honey From Apis spp. and *Trigona* spp. Bees.”

[fsn371645-bib-0352] Sutrisno, S. 2024. “Yuan Mandira Wicaksana; Atik Setiawan Wahyuningsih Analysis of Wound Treatment in Patients With Diabetic Foot Ulcer (DFU) Using Honey and Aloe Vera Extracts.” Journal of Scientific Research, Education, and Technology (JSRET) 3: 328–332. 10.58526/jsret.v3i1.365.

[fsn371645-bib-0353] Tahirovića, I. , H. Kurtagić , and N. Smječanin . 2023. “Correlations of Flavonoids Content and Antioxidant Activity in Bee Honey From Bosnia and Herzegovina.” Emirates Journal of Food and Agriculture 35: 262–270. 10.9755/ejfa.2023.v35.i4.3009.

[fsn371645-bib-0354] Talebi, M. , M. Talebi , T. Farkhondeh , and S. Samarghandian . 2020. “Molecular Mechanism‐Based Therapeutic Properties of Honey.” Biomedicine & Pharmacotherapy 130: 110590. 10.1016/j.biopha.2020.110590. 32768885

[fsn371645-bib-0355] Tananaki, C. , M.‐A. Rodopoulou , M. Dimou , D. Kanelis , and V. Liolios . 2024. “The Total Phenolic Content and Antioxidant Activity of Nine Monofloral Honey Types.” Applied Sciences 14: 4329. 10.3390/app14104329.

[fsn371645-bib-0356] Tang, Y. , L. Chen , and X. Ran . 2024. “Efficacy and Safety of Honey Dressings in the Management of Chronic Wounds: An Updated Systematic Review and Meta‐Analysis.” Nutrients 16: 2455. 10.3390/nu16152455. 39125335 PMC11314015

[fsn371645-bib-0357] Tao, M.‐H. 2019. “Epidemiology of Lung Cancer.” Lung Cancer and Imaging 25: 1–15.

[fsn371645-bib-0358] Tarapatskyy, M. , P. Sowa , G. Zaguła , M. Dżugan , and C. Puchalski . 2021. “Assessment of the Botanical Origin of Polish Honeys Based on Physicochemical Properties and Bioactive Components With Chemometric Analysis.” Molecules 26: 4801. 10.3390/molecules26164801. 34443389 PMC8398947

[fsn371645-bib-0359] Tashkandi, H. 2021. “Honey in Wound Healing: An Updated Review.” Open Life Sciences 16: 1091–1100. 10.1515/biol-2021-0084. 34708153 PMC8496555

[fsn371645-bib-0360] Tel‐Çayan, G. , B. H. Çiftçi , M. Taş‐Küçükaydın , et al. 2023. “Citrus Honeys From Three Different Regions of Turkey: HPLC‐DAD Profiling and In Vitro Enzyme Inhibition, Antioxidant, Anti‐Inflammatory and Antimicrobial Properties With Chemometric Study.” Chemistry & Biodiversity 20: e202300990. 10.1002/cbdv.202300990. 37548632

[fsn371645-bib-0361] Terzo, S. , P. Calvi , D. Nuzzo , et al. 2023. “Long‐Term Ingestion of Sicilian Black Bee Chestnut Honey and/or D‐Limonene Counteracts Brain Damage Induced by High Fat‐Diet in Obese Mice.” International Journal of Molecular Sciences 24: 3467. 10.3390/ijms24043467. 36834882 PMC9966634

[fsn371645-bib-0362] Tian, L. , S. Bilamjian , D. Cuthbertson , et al. 2024. “Impact of Processing Steps (Filtration, Creaming and Pasteurization) on the Botanical Classification of Honey Using LC‐QTOF‐MS.” Food Research International 194: 114841. 10.1016/j.foodres.2024.114841. 39232502

[fsn371645-bib-0363] Tiang, E. R. , L. Han , and F. Hu . 2025. “Physicochemical Characteristics, Antioxidant Capacity, and Antimicrobial Activity of Stingless Bee Honey From Malaysia: *Heterotrigona itama* , Lophotrigona Canifrons, and *Tetrigona binghami* .” Food 14: 995. 10.3390/foods14060995. PMC11941359 40232034

[fsn371645-bib-0364] Tlak Gajger, I. , S. A. Dar , M. M. M. Ahmed , M. M. Aly , and J. Vlainić . 2025. “Antioxidant Capacity and Therapeutic Applications of Honey: Health Benefits, Antimicrobial Activity and Food Processing Roles.” Antioxidants 14: 959. 10.3390/antiox14080959. 40867855 PMC12383161

[fsn371645-bib-0365] Tlak Gajger, I. , D. Pavliček , V. Oreščanin , I. Varenina , M. Sedak , and N. Bilandžić . 2024. “Mineral Concentrations in Different Types of Honey Originating From Three Regions of Continental Croatia.” Food 13: 2754. 10.3390/foods13172754. PMC11394878 39272517

[fsn371645-bib-0366] Tsiapara, A. V. , M. Jaakkola , I. Chinou , et al. 2009. “Bioactivity of Greek Honey Extracts on Breast Cancer (MCF‐7), Prostate Cancer (PC‐3) and Endometrial Cancer (Ishikawa) Cells: Profile Analysis of Extracts.” Food Chemistry 116: 702–708. 10.1016/j.foodchem.2009.03.024.

[fsn371645-bib-0367] Ünlü, A. E. , F. Altunkılıç , Y. Asgari , et al. 2024. “Property‐Wound Healing Relationship of Manuka‐, Anzer‐ and Chestnut‐Honey: Characterization, Antibacterial Properties and Cell Culture Applications.” Turkish Journal of Agriculture‐Food Science and Technology 12: 1826–1834. 10.24925/turjaf.v12i10.1826-1834.6869.

[fsn371645-bib-0368] van den Berg, A. J. J. , E. van den Worm , H. C. Quarles van Ufford , S. B. A. Halkes , M. J. Hoekstra , and C. J. Beukelman . 2008. “An In Vitro Examination of the Antioxidant and Anti‐Inflammatory Properties of Buckwheat Honey.” Journal of Wound Care 17: 172–178. 10.12968/jowc.2008.17.4.28839. 18494436

[fsn371645-bib-0369] Vanhanen, L. P. , A. Emmertz , and G. P. Savage . 2011. “Mineral Analysis of Mono‐Floral New Zealand Honey.” Food Chemistry 128: 236–240. 10.1016/j.foodchem.2011.02.064. 25214355

[fsn371645-bib-0370] Waheed, M. , M. B. Hussain , A. Javed , et al. 2019. “Honey and Cancer: A Mechanistic Review.” Clinical Nutrition 38: 2499–2503. 10.1016/j.clnu.2018.12.019. 30639116

[fsn371645-bib-0371] Wahyono, T. , A. M. Benita , I. M. Pratama , et al. 2024. “Effect of Gamma Irradiation and Evaporation on Physicochemical, Antibacterial, and Antioxidant Activity of Selected High‐Quality Herbal Honey.” Radiation Physics and Chemistry 214: 111263. 10.1016/j.radphyschem.2023.111263.

[fsn371645-bib-0372] Wang, H. , L. Li , X. Lin , W. Bai , G. Xiao , and G. Liu . 2023. “Composition, Functional Properties and Safety of Honey: A Review.” Journal of the Science of Food and Agriculture 103: 6767–6779. 10.1002/jsfa.12720. 37209396

[fsn371645-bib-0373] Wang, W. , X. Li , D. Li , et al. 2023. “Effects of Major Royal Jelly Proteins on the Immune Response and Gut Microbiota Composition in Cyclophosphamide‐Treated Mice.” Nutrients 15: 974. 10.3390/nu15040974. 36839331 PMC9967945

[fsn371645-bib-0374] Watanabe, J. , M. Nishimukai , H. Taguchi , et al. 2008. “Prebiotic Properties of Epilactose.” Journal of Dairy Science 91: 4518–4526. 10.3168/jds.2008-1367. 19038926

[fsn371645-bib-0375] Wilczyńska, A. , and N. Żak . 2024. “Polyphenols as the Main Compounds Influencing the Antioxidant Effect of Honey—A Review.” International Journal of Molecular Sciences 25: 10606. 10.3390/ijms251910606. 39408935 PMC11477350

[fsn371645-bib-0376] Wolde, R. , and S. A. Mahamed . 2024. “Antibacterial Activity of *Apis mellifera* Bees Honey, Garlic Extracts and Their Combinations Against Salmonella in Wolayta Sodo, Southern Ethiopia.” Svāsthya: Trends in General Medicine and Public Health 1: e25. 10.70347/svsthya.v1i2.25.

[fsn371645-bib-0377] Won, S.‐R. , C.‐Y. Li , J.‐W. Kim , and H.‐I. Rhee . 2009. “Immunological Characterization of Honey Major Protein and Its Application.” Food Chemistry 113: 1334–1338. 10.1016/j.foodchem.2008.08.082.

[fsn371645-bib-0378] Wu, J. , B. Han , X. Chen , et al. 2023. “Quantification of Bioactive Components and Evaluation of Microbial Community and Antibacterial Activity From Heterotrigona Itama and *Tetrigona binghami* Honeys.” International Journal of Food Science and Technology 58: 2247–2257. 10.1111/ijfs.16325.

[fsn371645-bib-0379] Wu, J. , S. Zhao , X. Chen , et al. 2023. “Physicochemical Properties, Multi‐Elemental Composition, and Antioxidant Activity of Five Unifloral Honeys From *Apis cerana* Cerana.” Food Science and Biotechnology 32: 1821–1829. 10.1007/s10068-023-01288-z. 37781061 PMC10541361

[fsn371645-bib-0380] Xin, L. Y. , H. Taib , Z. Berahim , A. Ahmad , and S. Lailatul Akmar Zainuddin . 2015. “The Effect of Tualang Honey on Human Periodontal Ligament Fibroblast Proliferation and Alkaline Phosphatase Level (Kesan Madu Tualang Ke Atas Percambahan Fibroblas Ligamen Periodontium Manusia Dan Paras Enzim Fosfatase Beralkali).” Sains Malaysiana 44: 1021–1025.

[fsn371645-bib-0381] Yaacob, M. , N. F. Rajab , S. Shahar , and R. Sharif . 2017. “Stingless Bee Honey and Its Potential Value: A Systematic Review.” Food Research 2: 124–133. 10.26656/fr.2017.2(2).212.

[fsn371645-bib-0382] Yaghoobi, N. , N. Al‐Waili , M. Ghayour‐Mobarhan , et al. 2008. “Natural Honey and Cardiovascular Risk Factors; Effects on Blood Glucose, Cholesterol, Triacylglycerole, CRP, and Body Weight Compared With Sucrose.” Scientific World Journal 8: 463–469. 10.1100/tsw.2008.64. 18454257 PMC5848643

[fsn371645-bib-0383] Yang, B. , Y. Lin , Y. Huang , N. Zhu , and Y.‐Q. Shen . 2023. “Extracellular Vesicles Modulate Key Signalling Pathways in Refractory Wound Healing.” Burns & Trauma 11: tkad039. 10.1093/burnst/tkad039. 38026441 PMC10654481

[fsn371645-bib-0384] Yaribeygi, H. , M. Maleki , B. Forouzanmehr , et al. 2024. “Exploring the Antioxidant Properties of Semaglutide: A Comprehensive Review.” Journal of Diabetes and Its Complications 38: 108906. 10.1016/j.jdiacomp.2024.108906. 39549371

[fsn371645-bib-0385] Yu, W. , Y. Zhang , J. Li , et al. 2025. “Physicochemical Properties, Chemical Composition, and Against *Klebsiella pneumoniae* Mechanism of Scrophularia Ningpoensis Honey.” LWT 228: 118157. 10.1016/j.lwt.2025.118157.

[fsn371645-bib-0386] Yupanqui Mieles, J. , C. Vyas , E. Aslan , G. Humphreys , C. Diver , and P. Bartolo . 2022. “Honey: An Advanced Antimicrobial and Wound Healing Biomaterial for Tissue Engineering Applications.” Pharmaceutics 14: 1663. 10.3390/pharmaceutics14081663. 36015289 PMC9414000

[fsn371645-bib-0387] Yusof, N. , A. H. Ainul Hafiza , R. M. Zohdi , and M. Z. A. Bakar . 2007. “Development of Honey Hydrogel Dressing for Enhanced Wound Healing.” Radiation Physics and Chemistry 76: 1767–1770. 10.1016/j.radphyschem.2007.02.107.

[fsn371645-bib-0388] Zainuddin, A. N. Z. , N. N. Mustakim , F. A. Rosemanzailani , N. I. M. Fadilah , M. Maarof , and M. B. Fauzi . 2025. “A Comprehensive Review of Honey‐Containing Hydrogel for Wound Healing Applications.” Gels 11: 194. 10.3390/gels11030194. 40136899 PMC11942582

[fsn371645-bib-0389] Zamri, N. A. , N. Ghani , C. A. N. Ismail , R. Zakaria , and N. Shafin . 2023. “Honey on Brain Health: A Promising Brain Booster.” Frontiers in Aging Neuroscience 14: 1092596. 10.3389/fnagi.2022.1092596. 36733498 PMC9887050

[fsn371645-bib-0390] Zapata‐Vahos, I. C. , J. C. Henao‐Rojas , D. P. Yepes‐Betancur , et al. 2023. “Physicochemical Parameters, Antioxidant Capacity, and Antimicrobial Activity of Honeys From Tropical Forests of Colombia: Apis Mellifera and *Melipona eburnea* .” Food 12: 1001. 10.3390/foods12051001. PMC10000824 36900518

[fsn371645-bib-0391] Zhang, W. , S. Chattrakarn , A. Sharrocks , and C. Tournier . 2023. “17P NRF2 Activation Promotes HER2‐Targeted Tolerance and Resistance in Oesophageal Adenocarcinoma Through Metabolic Reprogramming to Glutathione.” ESMO Open 8: 101663. 10.1016/j.esmoop.2023.101663.

[fsn371645-bib-0392] Zhao, H. , N. Cheng , W. Zhou , et al. 2019. “Honey Polyphenols Ameliorate DSS‐Induced Ulcerative Colitis via Modulating Gut Microbiota in Rats.” Molecular Nutrition & Food Research 63: 1900638. 10.1002/mnfr.201900638. 31533201

[fsn371645-bib-0393] Zhu, J. , H. Zhou , E. M. Gerhard , et al. 2023. “Smart Bioadhesives for Wound Healing and Closure.” Bioactive Materials 19: 360–375. 10.1016/j.bioactmat.2022.04.020. 35574051 PMC9062426

[fsn371645-bib-0394] Ziaei, R. , F. Shahdadian , M. Bagherniya , S. Karav , and A. Sahebkar . 2024. “Nutritional Factors and Physical Frailty: Highlighting the Role of Functional Nutrients in the Prevention and Treatment.” Ageing Research Reviews 101: 102532. 10.1016/j.arr.2024.102532. 39374829

[fsn371645-bib-0395] Zulkifli, N. A. , Z. Hassan , M. Z. Mustafa , et al. 2023. “The Potential Neuroprotective Effects of Stingless Bee Honey.” Frontiers in Aging Neuroscience 14: 1048028. 10.3389/fnagi.2022.1048028. 36846103 PMC9945235

